# GPCR systems coordinate cellular resilience against aging-associated stress

**DOI:** 10.3389/fmolb.2026.1771718

**Published:** 2026-04-07

**Authors:** Tabitha Boeringer, Mia Pardo, Carter J. Craig, Ainhoa Nieto Gutierrez, Derek R. Duckett, Patricia McDonald, Stuart Maudsley

**Affiliations:** 1 Receptor Biology Lab, Department of Drug Discovery, Moffitt Cancer Center, Tampa, FL, United States; 2 Discovery Sciences, Worldwide Research & Development, Pfizer Inc., Groton, CT, United States; 3 Department of Drug Discovery, Moffitt Cancer Center, Tampa, FL, United States; 4 Lexicon Pharmaceuticals Inc., Research & Development, The Woodlands, TX, United States

**Keywords:** adaptor, aging, allostasis, G protein-coupled receptor, resilience, stress, therapeutic

## Abstract

Aging-related diseases arise from cumulative cellular damage driven by diverse stressors. G protein-coupled receptors (GPCRs) play a critical role in coordinating cellular resilience to these stressors. We propose that stable multiprotein GPCR complexes—termed “receptorsomes”—function as adaptive hubs that sense, integrate, and mitigate stress across subcellular compartments. Receptorsomes comprise a GPCR core non-covalently associated with specific adaptors (e.g., β-arrestins, GIT2, RGS proteins, ASK1, YAP/TAZ), enabling pluridimensional, G protein-independent signaling that balances stress detection, damage repair, and homeostasis. This review synthesizes evidence linking receptosome composition to responses against oxidative, proteostatic, hypoxic, and other stressors, mapping these to aging hallmarks. We hypothesize that therapeutically tuning receptorsome adaptor engagement could enhance resilience, delay pathology, and extend health span, offering predictions for future testing.

## Introduction

Aging is a universal process that leads to cellular and tissue degradation across nearly all organisms. Multiple theories exist to explain the mechanisms that initiate and sustain aging, resulting in higher disease incidence risk and eventual mortality. Metabolically driven molecular stress, arising from diverse sources, is widely considered as one of the most powerful multidimensional triggers of aging (López-Otín et al., 2016; Golubev et al., 2017; Castañeda et al., 2022). Organisms strive to maintain physiological health to reach sexual maturity and reproduce, allowing a certain level of damage accumulation during this time. This damage arises both from internal factors, such as reactive oxygen species (ROS), and external factors, such as environmental radiation. This damage can affect nearly all types of molecules, including nucleic acids, lipids, and proteins. Damage accumulation links to metabolic/repair inefficiencies, creating feedback loops that accelerate aging (Kourtis and Tavernarakis, 2011). Aging is major risk factor for numerous disorders, including neurodegenerative diseases, dementia, diabetes, chronic kidney disease, vascular stiffness, cardiovascular disease, schizoaffective disorders, and cancer. Understanding how cells manage stress landscape is critical for developing therapies to slow aging and reduce age-related disease. Given the diverse nature of molecular stressors—such as ROS, altered pH, heat shock, and nutrient deprivation—effective therapeutic interventions likely require a highly adaptable response system. The G protein-coupled receptor (GPCR) system, known for its diversity and therapeutic potential, is one such system. We propose that specialized, interconnected GPCRs have evolved to detect, respond to, and mitigate the damage from these stressors, acting as a first line of defense against pathological aging and age-related diseases.

Building on this, we advance the concept of ‘stress resilience receptosomes’ as key organizers of GPCR-mediated cellular homeostasis control. Receptosomes are stable, pre-assembled multiprotein complexes where a GPCR transmembrane core is non-covalently linked to a repertoire of adaptor proteins, distinct from transient G protein ternary complexes in their relative stability, subcellular localization (e.g., plasma membrane, endosomes, Golgi, mitochondria, nucleus), and capacity for G protein-independent or biased signaling (Summarized in Table 1). This adaptor-centric architecture allows stress resilience receptosomes to function as modular “stress-management units,” with composition dictating specialization for specific stressors. Unlike standard GPCR-adaptor interactions, which are often transient and agonist-dependent, these stress-managing receptosomes enable proactive, pluripotent responses that predict and mitigate damage accumulation, promoting healthy aging.

**TABLE 1:** The receptosome model of stress resilience - key features and predictions.

Core Definition:
Receptorsomes are stable GPCR-adaptor superstructures that integrate stress sensing with adaptive signaling, differing from transient complexes by their pre-assembly and subcellular diversity
Key Features:
- Long-term physical complex stability
- Similar GPCRs can exist in multiple pre-formed receptosomes with functions based on adaptor stoichiometry
- Adaptor-driven specialization (e.g., GIT2 for DNA repair/oxidative stress; β-arrestins for ISR/mitophagy)
- Subcellular distribution enables localized responses (e.g., mitochondrial receptorsomes for ROS/hypoxia)
- Pluridimensional outputs balance immediate stress mitigation with long-term resilience
- Resilience receptosome dynamic ensemble may represent a prognostic indicator of cell health and disease
Testable predictions (Hypotheses):
- *Hypothesis 1*: Cells with diverse receptorsome repertoires (high adaptor variety) show superior multi-stressor resistance and slower aging phenotypes in models (testable via proteomics/single-cell sequencing)
- *Hypothesis 2*: Keystone adaptor disruption (e.g., GIT2 knockout) impairs multiplexed stress resilience more than single stressors (evidence from mouse models; testable in organoids)
- *Hypothesis 3*: Biased ligands favoring protective adaptors (e.g., β-arrestin over G proteins) extend health span in aging models (supported by preliminary pharmacology; testable in C. elegans/mice)
- *Hypothesis 4*: Receptorsome redistribution (e.g., to mitochondria/nucleus) correlates with aging progression (testable via imaging/subcellular fractionation)

## Signaling diversity in GPCRs

Following their discovery, GPCRs were considered as primarily G protein signaling entities. Recent work has evaluated that 516 FDA approved drugs directly target GPCRs. This scope represents approximately 36% of all FDA approved drugs (Caroli et al., 2025). This therapeutic dominance demonstrates the tremendous clinical importance of GPCR-based therapies. In the last decade effective therapeutic agents have been generated that modulate signaling preferences or ‘bias’ between different G protein signaling effects (Maudsley et al., 2004; Gesty-Palmer et al., 2006). In this context, it has been shown that receptor structural conformations for G protein activation are distinct between specific G protein expression pools, and that synthetic or naturally occurring ligand variants can selectively engender the formation of diverse receptor conformations (Maudsley et al., 2004; Kenakin, 2019). Multiple distinct forms of ‘agonist’ ligands for a single GPCR type have now been discovered that can selectively activate either a subset of G protein partners, a subset of downstream signaling effectors, or induce G protein coupling without initiating receptor internalization and desensitization (Maudsley et al., 2004; Kohout et al., 2004; Sagan et al., 1996).

Our understanding of GPCR signaling has grown through the illumination of diverse signaling functions emanating from GPCRs that can be G protein based, or through non-G protein signaling adaptors, e.g., β-arrestin (Donnelly et al., 1999; van Gastel et al., 2018a). The medicinal exploitation of GPCR-based G protein signaling has been tremendously successful, and it is likely that further investigation into non-G protein dependent signaling activity may result in even more profound results (Caroli et al., 2025; Maudsley et al., 2021; Ferguson, 2001; Coffa et al., 2011; Gurevich and Gurevich, 2018). The first non-G protein signaling function studied in depth was the β-arrestin-dependent activation of extracellular-signal regulated kinases 1/2 (ERK 1/2) (van Gastel et al., 2018a; Grundmann et al., 2018). This β-arrestin-linked ERK1/2 signaling pathway demonstrated a slower initial onset but eventually persisted over a longer period and also resulted in the creation of longer lasting cellular actions (Luttrell and Lefkowitz, 2002; Maudsley et al., 2005; Maudsley et al., 2015; Gesty-Palmer et al., 2013) compared to G protein signaling (Coffa et al., 2011; Maudsley et al., 2005). β-arrestin was initially considered simply as a negative regulatory protein for G protein coupling and receptor internalization (Gesty-Palmer et al., 2013; Pierce et al., 2000; Maudsley et al., 2013). Whether β-arrestin signaling represents an entirely distinct–and non-G protein dependent–phenomenon is a continued point of discussion. For example, definitive G protein independence failed to be shown in a serum starved *in cellula* G protein deletion model (Grundmann et al., 2018). In contrast, however, there is evidence that some orphan receptors (D6R and C5aR2) appear to only have the capacity to signal via β-arrestins and not via G proteins (Pandey et al., 2021). This result reinforces the proposal that a wide range of receptor-based protein superstructures (often termed ‘receptosomes,’ i.e., GPCRs coupled to a coterie of specific adaptor proteins (Maudsley et al., 2005)) are obligatorily coupled to specific, independent downstream signaling paths. We hypothesize that in any given circumstance a cell can create an effective collection of diverse receptosome superstructures that are designed to provide a pluripotent and adaptable cellular signaling output. The range and physico-chemical composition of these receptosome structures could potentially influence overall cell physiology/chemistry as well as vital stress response capacities.

## GPCR receptosome complexes

Following the first definitions of non-G protein-dependent signaling functions (Donnelly et al., 1999; Luttrell et al., 1999), it was evident that GPCR signaling was more complex than initially conceptualized by two-state or extended ternary complex models of GPCR function (Kenakin, 2019). A key factor that likely drives this complexity is the capacity of GPCRs to form stable, multiprotein signaling complexes, which are often referred to as ‘receptosomes.’ Receptosomes typically comprise the transmembrane helical core of the GPCR non-covalently connected to multiple interacting adaptor proteins. The number and type of these interacting adaptor partners engenders the creation of pre-formed receptor-based interactomic structures (Martin et al., 2009a). These pre-assembled receptosomes may be able to define a specific repertoire of signaling, subcellular trafficking/localization, desensitization/tachyphylaxis, and cell surface internalization features (Maudsley et al., 2005; Bockaert et al., 2004; Eo et al., 2007; Hanyaloglu and von Zastrow, 2008; Kreienkamp, 2002). While receptosomes exhibit relative stability compared to agonist-induced transient complexes, they are not static; adaptor exchange and subcellular trafficking allow dynamic adaptation to cellular conditions (Maudsley et al., 2005; Hanyaloglu and von Zastrow, 2008). This balance of stability and flexibility underpins their role in stress resilience, as evidenced by studies showing pre-formed complexes in non-stressed cells that reconfigure under oxidative or hypoxic cues (Maudsley et al., 2004; van Gastel et al., 2018a; Maudsley et al., 2015; Gesty-Palmer et al., 2013; Pandey et al., 2021; Martin et al., 2009a; Bockaert et al., 2004). This adaptor protein-encoded texturization of GPCR functionality–engendered by coordinated receptosome ensembles - is potentially affected by the relative variation of these adaptor proteins in distinct tissues and during contrasting times of cellular health/pathology, *e.g.,* between non-stressed and stressful cellular environments (Maudsley et al., 2004; Maudsley et al., 2021; Fagerberg et al., 2014).

## Subcellular localization of stress-sensitive receptosomes

With respect to the concept of differential compartmentalized signaling of GPCR systems it is prudent to distinguish established lines of evidence from those that are emerging with respect to this topic. It is likely that with further investigation into this aspect of GPCR biology further insights that may prove crucial for future drug discovery will be uncovered.

### Established GPCR signaling in endosomes

In well-established paradigms of GPCR signaling, agonist-activated receptors undergo phosphorylation by G protein-coupled receptor kinases (GRKs), leading to the recruitment of β-arrestins. These multifunctional adapter proteins not only uncouple receptors from heterotrimeric G proteins, thereby promoting desensitization, but also target the receptors to clathrin-coated pits for endocytosis, forming endosomal receptorsomes. Within these endosomal compartments, β-arrestins function as scaffolds, recruiting signaling proteins such as Src family tyrosine kinases and components of the ERK1/2 MAP kinase cascade. This sustains prolonged β-arrestin-dependent ERK signaling, which is critical for mediating extended cellular stress responses and represents a second wave of GPCR transduction independent of initial G protein activation (Luttrell and Lefkowitz, 2002). Recent updates in the field emphasize the role of biased signaling, where β-arrestin-mediated pathways, distinct from G protein signaling, contribute to unique biochemical and physiological outcomes, including modulation of endocytosis and targeted ERK activation in endosomes for processes like neutrophil degranulation and cellular adaptation to stress (Smith et al., 2018). Additional evidence supports the compartmentalization of this signaling, with non-canonical ERK activation occurring specifically at endosomes rather than the plasma membrane, dependent on β-arrestin recruitment and endosomal localization, which fine-tunes the duration and specificity of ERK dynamics in response to stressors (Kwon et al., 2022). Furthermore, MAPK-dependent regulation of endosomal GPCR/β-arrestin complexes influences receptor trafficking and downstream signaling, highlighting a feedback mechanism that prolongs ERK activity under stress conditions (Khoury et al., 2014).

### Emerging GPCR signaling in subcellular compartments

Emerging evidence points to GPCR signaling in non-traditional subcellular locales, such as mitochondria and the nucleus, though these mechanisms remain model-specific, context-dependent, and subject to ongoing debate, necessitating further validation through advanced imaging, proteomics, and functional assays. For mitochondrial signaling, GPR120 (also known as FFAR4), a free fatty acid receptor, responds to ligands like docosahexaenoic acid (DHA) to mitigate oxidative stress. Specifically, DHA activation of GPR120 triggers ERK1/2 signaling, which promotes PINK1/Parkin-mediated mitophagy, clearing damaged mitochondria and reducing reactive oxygen species (ROS) accumulation, thereby protecting hepatocytes from oxidative injury in models of liver stress (Zhang W. et al., 2021). This pathway is further supported in adipose tissue, where GPR120 regulates fatty acid oxidation and enhances mitochondrial function in brown adipocytes, contributing to metabolic homeostasis under stress, though its mitochondrial localization and direct effects require more confirmation (Ekechukwu and Christian, 2022). In nuclear signaling, β-arrestin-1 (βarr1) translocates from the cytoplasm to the nucleus upon GPCR activation (e.g., delta-opioid receptor stimulation), where it acts as a scaffold to recruit histone acetyltransferase p300 to specific gene promoters like those of p27 and c-fos. This facilitates enhanced histone H4 acetylation, promoting chromatin remodeling and increased transcription of genes involved in cellular responses to stress (Burns and Moniri, 2011). Subsequent studies have expanded this to other GPCRs, such as endothelin receptors, where nuclear βarr1 interacts with β-catenin and p300 to drive histone acetylation and Wnt pathway activation, influencing tumor progression in models like ovarian cancer, though these findings are debated due to cell-type specificity and potential off-target effects (Rosanò et al., 2013). Overall, while these emerging compartments offer novel insights into GPCR-mediated stress adaptation, their physiological relevance remains contentious and demands rigorous multi-omic validation to distinguish from canonical pathways.

## GPCR resilience systems

Dynamic organismal stress resistance is likely controlled by a tightly-regulated combination of multiple complex cellular systems. This resilience system has likely allowed for an average human life expectancy of about 80 years in industrialized countries. The organismal stress response network has probably evolved over billions of years through the interplay between nuanced stress sensory mechanisms, compensatory feedback loops, and stress-prediction systems involving GPCR-associated damage management programs (Chadwick and Maudsley, 2010).

The random and acute nature of stressful insults can often overcome cellular stress responsive networks. These response networks may only be able to respond at a much slower rate than the onset of damage and thus will likely result in persistence and exacerbation of damage when the next stressful event occurs (van Gastel et al., 2019a; van Gastel et al., 2018b; Chatzidoukaki et al., 2020; Yegorov et al., 2020; Murthy et al., 2020). This paradigm could be defined as a stress ‘tetanus’ and the prevention of this could significantly attenuate the rate of cellular damage accumulation. This potential damage tetanus through repetitive deleterious molecular insults is likely a key driver of disease and aging. The specific initiation of diseases therefore may potentially be engineered via a generic start point but then diversify over time to engender differential end-stage disease conditions (Maudsley et al., 2021; Belikov, 2019; Fransquet et al., 2020; Chen et al., 2022). GPCRs may represent a facile mechanism to control dynamic stress response networks given their well-characterized roles in a multitude of biological activities (Caengprasath and Hanyaloglu, 2019). Understanding how best to regulate and potentially reverse a deficit of systemic cellular/tissue homeostasis (linked to overwhelmed resilience mechanisms) is vital to the creation of the next-generation of anti-aging/damage therapeutics (Leysen et al., 2018).

Disease genesis and development probably occurs through a combination of intricate and complex signal transduction. In such scenarios the therapeutic effects of ‘monolithic target’ drugs may be potentially collateral to the observed changes in disease symptoms (Maudsley et al., 2021; Whitwell et al., 2020). Drug responses and molecular stressor states are complex multi-system processes; hence flexible investigation systems should be evaluated to allow a holistic appreciation of how both single index effects and multiple signaling cascades overlap to underpin the pluripotent stress response network. This intersection between different dimensions of responsive networks will likely create emergent signaling functions that could connect diverse elements of the nuanced response systems and potentially generate functional stress ‘predictive’ molecular programs (Azeloglu and Iyengar, 2015; Bertalanffy, 1972).

Aging is a multi-level somatic event that involves a wide range connected signaling actions. Even with this somatic level of complexity, investigations have identified more specific sub-processes that combine to generate the overall organismal aging event. These sub-processes have often been referred to as the ‘hallmarks’ of aging (López-Otín et al., 2016; López-Otín et al., 2013). With further advances in the understanding of dynamic processes in the aging paradigm, several modifications/adjustments to these classifications have been made (López-Otín et al., 2016; Harries, 2022; Blagosklonny, 2022). Recent refinements to the aging hallmarks incorporate disabled macroautophagy, chronic inflammation (inflammaging), and dysbiosis (López-Otín et al., 2023). Stress resilience GPCR receptosomes may intersect these via adaptor-mediated regulation: e.g., GIT2 promotes mitophagy/autophagy (Fu et al., 2022); β-arrestins modulate NF-κB/inflammaging (Li et al., 2023); nutrient-sensing GPCRs (e.g., GPR91/FFAR3) influence gut dysbiosis via microbial metabolite signaling (Burman and Kaji, 2021).

Hence, at the present time, it is often stated that one could group the various aspects of molecular aging into *Generative Mechanisms* (telomere attrition, genomic instability, epigenetic alterations, disrupted proteostasis), *Reactive Mechanisms* (defective nutrient sensing, mitochondrial dysregulation, cellular senescence/senescence-associated secretory phenotype), and *Gerophenotype Mechanisms* (disrupted cell-cell communication, stem cell depletion).

*Generative Mechanisms* involve molecular processes that instigate first actions of homeostatic instability. These can then drive the creation of either healthy or pathological aging phenotypes. *Reactive Mechanisms* comprise physiological processes that typically respond to perceived homeostatic instabilities and then attempt to reduce the frequency/longevity of the deleterious perturbations. In the presence of a prevailing loss of function/efficacy of the *Reactive* mechanisms, a resultant creation of the Gerophenotype (the overall state of aging physiology) will occur over time and will likely drive the development of specific aging-related diseases. A considerable degree of investigation has been undertaken with respect to the initial genesis of pathological aging; however, it is less clear how the convergence of multiple stress factors initiates the *Generative* mechanisms that relay these simple cellular actions into more complex biological processes such as telomere attrition and epigenetic programs.

The most likely experienced cellular stressors include oxidative damage; environmental radiation exposure; pathological protein modification (*e.g.,* acetylation/methylation); nucleic acid damage; nutrient sensation/usage dysfunction; mitochondrial dysfunction; molecular senescence drivers; hypoxia; pH imbalances; thermal stresses (heated or cooled); cytoskeletal or organelle-based stress (*e.g.,* endoplasmic reticulum stress (Chadwick et al., 2012a):). These stressful inputs can arise from both endogenous alterations in endocrine/neurological function as well as from external environmental sources. The impact of these stressors on cell biology is a common anticipated phenomenon for cells and therefore multiple stress response processes (e.g., the DDR (DNA damage response (Leysen et al., 2018)) or the ISR (integrated stress response (Costa-Mattioli and Walter, 2020; Nii et al., 2019))) have evolved over time to repair the potential damage uncontrolled stress effects can incur. While the impingement of these stressors is a daily expectation of cells, the frequency, magnitude, and temporal convergence of these stressful impacts could likely lead to pathological forms of concatenation (e.g., stress ‘tetanus’) and resultant amplification, which result in the initiation and augmentation of pro-aging molecular signaling paradigms.

Research has demonstrated that protective GPCR systems exist at a whole-body systemic level–these are likely created to contend with disease and aging actions (van Gastel et al., 2018b; Hendrickx et al., 2020; Geerts et al., 2016; Janssens et al., 2014; Leysen et al., 2021; Maudsley et al., 2012; Santos-Otte et al., 2019; Chapter et al., 2010; Brain Health Modeling Initiative BHMI, 2018). Protective GPCR signaling networks are also likely to exist *within* individual cells. GPCR receptosomes may be tasked with sensing, integrating, and responding to pro-aging/disease stressors. These pre-formed receptosomes are likely to be specifically distributed across a variety of cellular compartments including: at the cell surface; in subcellular organelles; or even within intracellular nanovesicles or non-membrane bound organelles (NMOs). These stress-sensitive receptosomes can then coordinate the cells’ ability to sense, respond to, ameliorate, and even predict the presence of new, stressful events. The variety, and number of stress-sensitive receptosomes structures that a cell can generate, maintain, and recreate in a regular basis is potentially one of the most important indicators of longitudinal stress resilience and cellular health. GPCRs for different orthosteric ligands may however subsequently be coupled to similar stress-associated adaptor proteins–these may then form a dedicated functional cluster that is not based on the identity of their orthosteric cognate ligand but on the type of stressor they deal with. Considerable research has already shown that multiple GPCR adaptors–that are associated with a broad range of GPCR types - can functionally converge with respect to the regulation of many stress-related activities, *e.g.,* β-arrestins (Nieto et al., 2020; Schattauer et al., 2019; Wang H. M. et al., 2014; Chen Y. et al., 2021; Theccanat et al., 2016), RGS proteins (Lee and Bou Dagher, 2016; Jung et al., 2016; Hwang et al., 2015), and GIT2 (Lu et al., 2015; Anckaerts et al., 2019; Silva et al., 2019; Seo SB. et al., 2018; Lee et al., 2014). Here we will delineate how complex GPCR systems can regulate stress response networks via their stable interactions with a diverse range of non-G protein signaling adaptors via the generation of stable superstructures.

### Nutrient sensing and management

Aging is widely attributed to progressive decline in glycometabolic function (Chadwick and Maudsley, 2010; Chatzidoukaki et al., 2020; Barabasi and Albert, 1999; van Gastel et al., 2021; Wang et al., 2021; Yegorov et al., 2020; Murthy et al., 2020), driven by accumulated molecular damage that impairs homeostatic energy control and elevates disease risk (Murthy et al., 2020). Metabolic homeostasis is a primary cellular goal to prevent stress-induced damage (Chen et al., 2022). Nutrient-sensing and management pathways are highly interconnected across tissues, forming a robust system for metabolic control under stress (López-Otín et al., 2016; Bettedi and Foukas, 2017; Efeyan et al., 2015). A complex neuroendocrine GPCR network regulates neurometabolic activity and contributes to neurodegenerative disorders (López-Otín et al., 2013), linking endocrine and neuronal systems in health and chronic disease (Chatzidoukaki et al., 2020; Wang et al., 2021; Yegorov et al., 2020; Murthy et al., 2020). These mechanisms likely operate at the single-cell level via coherent GPCR receptosome complexes sharing stress-responsive functions. Under nutrient sufficiency, conserved pathways promote anabolism and energy storage. During deficiency, cells mobilize internal stores via autophagy, while neuroendocrine axes assist in restoring balance (Martin et al., 2007; Martin et al., 2008; Martin et al., 2009b; Martin et al., 2009c; Cai et al., 2013). Nutrient-sensing dysregulation is common in aging-related metabolic conditions (Efeyan et al., 2015; Ondaro et al., 2022; Zhu et al., 2022; Corremans et al., 2022; Hinden et al., 2022; Green et al., 2022). Key systemic factors integrate signaling, including SIRT1, AMPK, mTORC1/2 (Torrence et al., 2021; Ricoult and Manning, 2013), and PI3K/AKT (Torrence et al., 2021; Ricoult and Manning, 2013). Multiple GPCR systems regulate these: sweet-taste (Martin et al., 2017; Cong et al., 2013; Cai et al., 2014a; Cai et al., 2014b) and bitter-taste receptors (Jaggupilli et al., 2019); gut luminal GPR91 and FFAR3 (Burman and Kaji, 2021; Shirazi-Beechey et al., 2014); cardiac Tas1r1/Tas2r108 (Foster et al., 2013); and fatty acid sensors GPR40 and GPR120 (Efeyan et al., 2015; Al et al., 2022). Beyond classical pathways (e.g., AMPK, mTOR (Hsu and Ip, 2011; Michel et al., 2014; Li et al., 2021)), GPCR activity also modulates downstream elements like YAP/TAZ-Hippo signaling (Zhou et al., 2015) and autophagy (Hsu and Ip, 2011).

### Cellular senescence

Repetitive cellular/tissue stress can activate senescence-related molecular programs. Senescent cells exhibit growth arrest and desensitization to extrinsic stimuli via GPCRs (Fransquet et al., 2020; Lipsitz and Goldberger, 1992; Sedivy, 1999; Sleimen-Malkoun et al., 2014). Cellular senescence represents a unique fate linking cell cycle cessation with resistance to apoptosis, resulting in distinct phenotypes involving chromatin remodeling and secretory activity compared to normal cells (Jia et al., 2017; Hu et al., 2016; Faner et al., 2014; Hayflick and Moorhead, 1961). The classic ‘replicative senescence’ phenotype, described by Hayflick and Moorhead (Hayflick and Moorhead, 1961), is associated with DNA instability and telomeric degradation (Gao et al., 2022). Recent evidence shows senescence is not always irreversible and contributes to wound healing/repair, embryonic development, and molecular aging (Santos-Otte et al., 2019; Di Micco et al., 2020). In addition to telomere attrition, stressors such as DNA lesions and ROS induce senescence (van Gastel et al., 2019a; Leysen et al., 2018). Senescence is regulated by ATM and ATR kinases, which stabilize p53 and mobilize the Cdk inhibitor p21 to block cell-cycle progression (Fielder et al., 2017). A hallmark of senescence is the senescence-associated secretory phenotype (SASP), characterized by pathological and pro-inflammatory changes in the cellular secretome (Leysen et al., 2018). Senescence involves significant chromatin remodeling that drives elevated transcription of pro-inflammatory cytokines, chemokines, growth factors, and proteases (Sikora et al., 2014). Both cell cycle arrest and SASP are closely tied to DNA damage and stress responses (van Gastel et al., 2019a), often perpetuated by feed-forward loops between ROS and DDR signaling (Leysen et al., 2018). Recent studies have explored how alterations in GPCR functionality and their adaptor associations regulate senescence and SASP processes (Leysen et al., 2018; Michna et al., 2016).

### Oxidative stressors

Oxidative stress is perhaps the most deleterious insult a cell can experience. Oxidative stress, which typically is induced by biologically generated reactive oxygen-containing molecular species, can be considered to function as both a detrimental cell stressor and a normal cell signaling modality (Brown and Griendling, 2015; Luo J. et al., 2020). Reactive oxygen species (ROS) generally include unstable oxygen radicals (*e.g.,* superoxide radicals or nonradicals such as hydrogen peroxide) that, at moderate concentrations, control physiological intracellular signaling functions, *e.g.,* neurotransmission (Beltrán González et al., 2020), receptor signal transduction (Chadwick et al., 2011a), and immune regulation (Dominic et al., 2022). To define matters, oxidative stress is a state that is caused by a temporary imbalance between ROS generation and the presence of available endogenous antioxidants such as glutathione, NADPH (reduced nicotinamide adenine dinucleotide phosphate), or ROS scavenging factors such as SOD (superoxide dismutase). Reductions in exogenous (*e.g.,* dietary) or endogenous antioxidants promote cellular ROS levels, leading to an increased propensity for oxidative radical interaction with cell/tissue lipids, proteins, and nucleotides (Luo J. et al., 2020; Chadwick et al., 2011a; Chadwick et al., 2010a; Liguori et al., 2018). As with most physiological stress systems, the phenomenon termed ‘hormesis’ (*i.e.,* stress being the cause of beneficial anti-stress responses (Chadwick and Maudsley, 2010)) can apply to ROS-based damage. Hence it has been shown that low ROS levels–causing a minimal degree of damage–appear to be beneficial to organisms and may even extend lifespan in *Caenorhabditis elegans* or yeast and experimental models. These data illustrate the protective role of ROS through controlling cell proliferation and survival responses during normal stress and/or physiological conditions (Lin L. et al., 2019). ROS are continually produced during normal aerobic metabolism via the mitochondrial electron transport chain in. Therefore, mitochondria hold a prominent position with respect to controlling the balance between health and disease/aging as they are simultaneously a source of both energy-regulating ATP as well as ROS (Del Valle, 2011). Data have revealed that ROS are not produced in a random or unregulated manner. In normal, healthy cells, ROS production rates are typically low with approximately 0.1 nM formed hydrogen peroxide min/mg mitochondrial protein (Buffenstein et al., 2008). Mitochondrial damage/dysfunction–a typical factor in the aging process–often results in the accumulation of ROS above healthy physiological levels. This excessive ROS can then overwhelm extant cellular antioxidant systems, resulting in irreversible molecular damage to cellular macromolecules that drives longitudinal damage. This accumulated damage can affect multiple interconnected cellular functions (proteasomal function, ATP levels, resting membrane potential, etc.), which if they persist, can drive cellular senescence, aging, and disease.

Data from multiple research groups has shown that many GPCR systems (across all major structural GPCR classes) demonstrate a tight signaling relationship with oxidative stress activity, *e.g.,* relaxin-3 receptors (van Gastel et al., 2019a), melatonin receptors (Basha and Hemalatha, 2022), adropin-sensitive receptors (Thapa et al., 2018), serotonin (5-HTR1E) receptors (Sharma et al., 2021), adhesion (ADGRG1) receptors (Chen Y. et al., 2021), Formyl-Peptide (FPR2) receptors (Caso et al., 2021), proton-sensing (GPR4) receptors (Haque et al., 2020), prosaptide (GPR37) and prosaposin (GPR37L1) receptors (Meyer et al., 2013), and succinate-sensitive (GPR91) receptors (Ariza et al., 2012). In the aging brain, in a system dominated by oxidative damage, microglial GPCRs (e.g., P2Y purinergic, adenosine A2A, chemokine CCR5) form receptosomes that modulate inflammaging and resilience (Aldossary et al., 2020; Gao et al., 2023). β-arrestin-biased signaling via these attenuates ROS/NF-κB, as evidenced in hypoxia models (Franco et al., 2018). Emerging studies link Gq/Gi-coupled receptors in anterior cingulate circuits to behavioral resilience, suggesting receptosome adaptors (e.g., RGS proteins) as hubs for neuroprotection (Lee and Bou Dagher, 2016).

Cellular oxidative attack can also disturb subcellular receptor trafficking, *e.g.,* human parathyroid hormone (PTH) receptor (Ardura et al., 2017), suggesting an impact of stress upon specific receptor adaptors linked to receptor endocytosis such as β-arrestin. In addition, there are several examples of GPCR systems that demonstrate a capacity to coherently integrate multiple stress responses, hence reinforcing our proposal that a subcellular network of interconnected GPCRs regulates stress resilience and potential cell fate decision. Illustrating this stress integration principle, melatonin receptors can integrate redox sensation, ER (endoplasmic reticulum) stress, and the UPR (unfolded protein response) (Basha and Hemalatha, 2022). A further demonstration of GPCR stress integration can be found with the cardiac beta-adrenergic receptor that functionally links the nutrient-sensing AMPK signaling cascade to the oxidative stress sensory pathway (Zhao et al., 2024). With respect to the additional effects of GPCR-associated adaptor proteins and their capacity to sense oxidative stress, multiple factors have been shown including GIT2 (van Gastel et al., 2019a), GRK2 (G protein-coupled receptor kinase 2 (Cobelens et al., 2006)), and ASK1 (Apoptosis signal-regulating kinase 1) (Matsukawa et al., 2004).

### Proteostatic stressors

Under random stress, cells strive to minimize proteostatic disturbances. Disruptions in proteostasis—arising from protein misfolding, mislocalization, or aggregation—generate functional cellular stress. The proteostasis network, comprising molecular chaperones, proteolytic enzymes, and their regulators, maintains proteostatic function to mitigate aging-related damage from improper protein processing (Hipp et al., 2019). This network also governs post-translational modifications such as acetylation, methylation, and nitrosoation. Aging-associated stress typically diminishes proteostasis capacity, leading to compromised proteome integrity and function. The resulting accumulation of misfolded and aggregated proteins, particularly in post-mitotic cells, is implicated in numerous age-related disorders, including Alzheimer’s and Parkinson’s disease. Alterations in the GPCR-associated kinase GRK2 can induce proteostatic stress, causing unregulated protein nitrosoation and aging-related inflammation (Kawakami et al., 2018). Dysregulated GRK2 coupling to GPCRs is connected to the nitric oxide system, potentially promoting aberrant vascular protein nitrosation and contributing to vascular aging. GPCR signaling also regulates protein deacetylation, as demonstrated with the alpha1-adrenergic receptor (Chiu et al., 2021), and can control histone acetylation linked to tumor generation (Zheng et al., 2020). Many GPCR-mediated effects on protein acetylation involve β-arrestin coupling (Kang et al., 2005). GPCRs themselves are subject to proteostasis-related modifications, such as S-sulfonation of the beta2-adrenergic receptor (Burns and Moniri, 2011). Therapeutic enhancement of proteostasis networks may delay the onset of age-related pathologies driven by proteome deterioration.

### Electromagnetic/radiation stressors

Ionizing radiation is a potent mechanism to induce multiple forms of cellular stress. Exposure to direct high-energy ionizing radiation is typically the result of close proximity to research sources or energy generation-linked radioisotopes. Environmental lifestyle exposure to ionizing radiation outside of these scenarios typically leads to low-level, chronic exposure. Along with cellular damage caused by ionizing radiation, lower energy electromagnetic (EM) radiation effects on cellular function and stress resistance have been studied recently (Schuermann and Mevissen, 2021). The role of GPCR systems in stress response networks has been shown through molecular manipulation studies. For example, the LRG4 receptor (Leucine-rich repeat-containing G protein-coupled receptor 4) appears to possess a positive stress resistance role in the face of ionizing radiation, as LGR4 gene deletion results in an increased radioisotope-sensitivity of prostate cancer cells (Liang et al., 2021). In addition, it has also been shown that the functionality of the LPA2 (Lysophosphatidic acid receptor 2) receptor is regulated by the cellular response to ionizing radiation exposure (Balogh et al., 2015). Aside from damage-induced changes to GPCR systems, it has also been demonstrated that *in vivo* deletion of β-arrestin-1 can alter the radiation sensitivity of experimental mouse models (Nieto et al., 2020).

### Nucleic acid attrition

DNA undergoes pathological alterations through mechanisms such as nucleotide mutations, substitutions, deletions, insertions, large adduct formation, single-strand breaks (SSBs), and double-strand breaks (DSBs) (Leysen et al., 2018; Rodriguez-Rocha et al., 2011). Cells counter this damage via the DNA Damage Response (DDR), a nuanced signaling network. The DDR process involves: (1) detection of damage sites, (2) signaling to coordinating transducers, (3) activation of cell-cycle checkpoints, and (4) recruitment of appropriate repair proteins (Ciccia and Elledge, 2010). Key DDR pathways in human cells include base excision repair (BER), mismatch repair (MMR), nucleotide excision repair (NER), homologous recombination (HR), and non-homologous end-joining (NHEJ) (Ambekar et al., 2017). BER corrects single-base lesions or small alterations, often induced by ROS from nutrient deprivation stress (Ambekar et al., 2017; David et al., 2007). It begins with DNA glycosylase recognition (Sancar et al., 2004), followed by AP endonuclease activation to generate a 3′OH terminus (Hegde et al., 2008), then DNA polymerase synthesis and ligase sealing. MMR corrects post-replication base mispairs and small insertion/deletion loops from replication errors (Modrich, 2016). NER removes helix-distorting lesions caused by chemicals, UV radiation, or protein-DNA adducts (Ambekar et al., 2017; Sancar et al., 2004), including transcription-coupled repair of blocking lesions. HR and NHEJ primarily repair DSBs (Leysen et al., 2018; Sancar et al., 2004; Shrivastav et al., 2008); replication fork-associated damage is repaired almost exclusively by HR (Shen and Nickoloff, 2007), initiated by 3′OH overhang formation, Rad52 association, and Rad51 polymerization (Jasin and Rothstein, 2013). NHEJ starts with Ku70/Ku80 heterodimer binding to DSBs (Mari et al., 2006; Davis and Chen, 2013), recruiting other factors including DNA-PKs. Stress-induced DNA damage occurs daily at chromosomal and nucleotide levels. Telomeres maintain chromosomal stability through repetitive sequences and the Shelterin complex, protecting exposed ends (Aubert and Lansdorp, 2008; de Lange, 2005). Telomeres progressively shorten with cell divisions (de Lange, 2005; Lu and Pickett, 2022), limiting replicative capacity. Shortened telomeres trigger senescence in normal cells or genomic instability in pre-malignant cells (Barnes et al., 2019). Oxidative stress accelerates telomere shortening and dysfunction (Reichert and Stier, 2017; Kliment and Oury, 2010; Graham and Meeker, 2017; Jurk et al., 2014; Cattan et al., 2008; Kaul et al., 2011; Lidzbarsky et al., 2018), potentially via compensatory cell divisions after oxidative loss (Barnes et al., 2019) or direct ROS-induced SSBs leading to replication fork collapse and telomere degradation (Jurk et al., 2014). The GPCR adaptor β-arrestin integrates DDR signaling. β-arrestin-1 links oxidative stress responses (via Thioredoxin-1) to γ-H2AX-p53 DDR pathways (Jia et al., 2014). Ionizing radiation upregulates CXCR4 through an ATM-HIF1α-dependent cascade, enhancing inflammatory responses upon CXCL12 activation and contributing to chronic aging-related inflammation (Nair et al., 2018; Di Giosia et al., 2022). The orphan GPCR GPR17 regulates DDR protein expression, influencing cell growth in cancers such as glioblastoma multiforme (Kaul et al., 2011; Doan et al., 2021). DDR-associated diseases (Hutchinson-Gilford Progeria Syndrome, Werner Syndrome, Cockayne Syndrome, Ataxia-Telangiectasia) link to dysglycemia and insulin resistance (Caux et al., 2003; Okamoto et al., 1992; Hayashi et al., 2015; Bar et al., 1978). Given the convergence of insulin decline, oxidative stress, DNA damage, and advanced aging, interventions targeting this signaling axis hold promise for treating age-related disorders.

### Hypoxic stress

Functional hypoxia, defined as deficits in cellular oxygen levels, is a major stressor and hallmark of pathophysiological states including inflammation, cancers, cardiovascular disorders, and premature aging (Lee et al., 2020). It commonly arises from inadequate perfusion with poorly oxygenated blood or dysfunctional lung ventilation, disrupting oxidative phosphorylation, generating ROS, and activating NFκB to drive pro-inflammatory cytokine release (e.g., IL-1, IL-6, TNFα) (Stelzner et al., 1988; Totzeck et al., 2014). GPCRs play pivotal roles in adaptive hypoxic responses, with hypoxia rapidly altering GPCR function via transcriptional regulation (often mediated by HIF-1α (Lee et al., 2013)) or post-translational modifications affecting localization, stability, adaptor scaffolding, or signaling (Kimura et al., 2001; Adams et al., 2008). Multiple GPCRs regulate HIF-1α activity under hypoxia (Recchia et al., 2011; Guo et al., 2014; Franco et al., 2019), inducing its expression and function (Lee et al., 2013; Hu et al., 2009) to mimic hypoxic conditions. Examples include activation by endothelin-1 (EDNRA), β-adrenoceptor agonists (ADRB receptors), or lysophosphatidic acid (LPAR1/2), which recruit transcription factors to the HIF-1α promoter and stabilize the protein (Lee et al., 2013; Hu et al., 2009; Lappano et al., 2016). The orphan receptor GPR41 acts as a hypoxia-induced GPCR, promoting p53-dependent apoptosis in ischemic/reoxygenated cardiomyocytes (Kimura et al., 2001). Oxygen and glucose deprivation enhances CB2R signaling in CNS neurons, elevating CB2R-5-HT1A heteroreceptor complexes proposed to mitigate ischemic brain injury (Franco et al., 2019). Genetic deletion of β-arrestin-2 (ARRB2) impairs cardiac progenitor resilience to hypoxia, highlighting ARRB2-containing receptosomes in cell survival (Seo SK. et al., 2018). Acute hypoxia modulates ADRB2 signaling via altered PKA expression (Tripathi et al., 2020), with ADRB2 functioning as a hypoxia sensor through GRK2-containing receptosome complexes (Nijboer et al., 2008). In cardiac tissues, 5-HTR3 and 5-HTR4 receptors modulate mitochondrial activity; their knockout exacerbates hypoxic damage in neonatal cardiomyocytes, suggesting roles in managing [Ca^2+^], ROS, and ATP (Guo et al., 2014). The bile acid receptor TGR5 regulates hypoxic hepatic stress responses via Nrf2-Keap1 expression and distribution (Zhuang et al., 2021). Genomic deletion of GPR22 attenuates cardiac resistance to hemodynamic hypoxia (Adams et al., 2008). Hypoxic microenvironments disrupt membrane protein localization, altering GPCR effector scaffolding and signaling stoichiometry. Hypoxia also affects GPCR post-translational modifications (phosphorylation, glycosylation, acylation, nitrosoation, prenylation), influencing receptosome composition, localization, and specificity (Chakraborty et al., 2017). Single-cell hypoxic adaptation involves suppressing ATP-consuming reactions and redirecting metabolism to restore oxygen homeostasis (Chadwick et al., 2011b; Burtscher et al., 2022). These responses interconnect genes regulating survival, angiogenesis, glycolysis, invasion, and metastasis (Chakraborty et al., 2017), enabling long-term adaptations in nutrient acquisition, protein synthesis, mitochondrial respiration, and lipid/carbon metabolism. Beyond single-cell sensing, GPCRs modulate global hypoxia sensitivity via carotid body actions (e.g., A2A and 5-HT2 receptors controlling ventilation to reduce hypoxic periods) (Aldossary et al., 2020; Moya and Powell, 2018).

### Organelle-based stress

Pathological convergence of multiple stressors, e.g., oxidative stress, hypoxia, depletion of growth factors and nutrients, and disturbances to proteostasis can cause the intracellular buildup of misfolded proteins. These poorly-processed and misfolded proteins tend to accumulate in the endoplasmic reticulum (ER) where they ultimately induce a cellular condition referred to as ER stress (ERS). In normal conditions, this stress is typically dealt with through a coherent cellular program called the unfolded protein response (UPR). Multiple GPCRs possess the capacity to regulate and manage both the ERS and UPR. These ERS/UPR-controlling receptors exert this function via multiple functional interactions with trophic regulating factors such as IRE1α (inositol-requiring enzyme 1 α), ATF6 (Activating transcription factor 6), and PERK (protein kinase R (PKR)-like endoplasmic reticulum kinase) (Chadwick et al., 2012a; Kumari et al., 2021).

Disruption of ER homeostasis promotes the accumulation of unfolded proteins/misfolded proteins in the lumen of the ER resulting in the generation of ERS (Kumari et al., 2021; Mesgarzadeh et al., 2022). To cope with the accumulation of unfolded proteins/misfolded proteins in the lumen of the ER (Kumari et al., 2021; Mesgarzadeh et al., 2022), cells activate the UPR. This involves a series of signaling mechanisms that aim to reduce ER load by temporarily inhibiting global translation and refolding or via degrading the accumulated misfolded proteins (Wodrich et al., 2022).

The three primary UPR sensors, ATF6, IRE1α, and PERK, are held in an inactive state by their association with ER membrane chaperone GRP78/BIP (glucose-regulated protein 78/binding immunoglobulin protein). Misfolded proteins in the ER induce the release of ATF6, IRE1, and PERK from GRP78, allowing their functional activation (Ibrahim et al., 2019).

ATF6 is a type II transmembrane protein and a member of the bZIP (basic leucine zipper) transcription factor family. IRE1α is a type I transmembrane protein with a serine/threonine kinase domain and an endoribonuclease domain located on the cytosolic side of the protein. PERK is a type I transmembrane protein with a serine/threonine kinase domain. To reduce the protein load in the ER during times of ERS, IRE1-dependent decay (RIDD) is activated (Hollien and Weissman, 2006). IRE1 can also induce the tumor necrosis factor receptor- (TNFR-) associated factor 2 (TRAF2)/apoptosis signal-regulating kinase 1 (ASK1)/JNK cascade, which also contributes to activation of cell death (Urano et al., 2000). With respect to the intersection of GPCR receptosome biology with the ERS, the ASK1/JNK cascade has been shown to be orchestrated and scaffolded by the GPCR adaptor β-arrestin-2 (McDonald et al., 2000; Miller et al., 2001).

Activated PERK kinase has been shown to increase levels of transcription factor ATF4, which induces amino acid biosynthesis and phosphorylates Nrf2 (Nuclear factor erythroid 2-related factor 2), thereby controlling antioxidant response. One of the direct targets of ATF4 is C/EBP homologous protein (CHOP), which stimulates genes responsible for mitochondrially-associated apoptosis. CHOP and ATF4 act together to control multiple autophagy-related genes, p62, Atg5, Atg7, and Atg10 (Novoa et al., 2001). CHOP also activates DNA damage-inducible protein (GADD34/PPP1R15A: Protein phosphatase 1 regulatory subunit 15A) to dephosphorylate eIF2α and resume protein translation to attenuate PERK signaling (Novoa et al., 2001; Lin Y. et al., 2019). In times of chronic ERS, activation of the UPR can eventually lead to lysosome-mediated autophagy through a variety of signaling mechanisms, e.g., the IRE1-JNK-Bcl-2, PERK-eIF2α-ATF4, or ATF6-XBP1-Atg axes (Kania et al., 2015; Senft and Ronai, 2015).

In recent times the ERS and UPR have been unified in principle under the umbrella of the newly codified ‘integrated stress response (ISR)’ (Pakos-Zebrucka et al., 2016). The ISR comprises coordinated cellular responses to viral infections, amino acid deficits, heme deprivation, and ERS. Response pathways in the ISR ultimately converge upon eIF2α and ATF4. Several GPCRs have recently been shown to control the functionality of this multivalent stress response programs like the ISR, e.g., the orphan GPR132 receptor (Nii et al., 2019), the dopamine receptor D2 (DRD2) (Prabhu et al., 2020) and the leucine-rich-repeat-containing GPCR 5 (LGR5) (Theccanat et al., 2016).

### Mitophagy

Mitochondria are currently considered to possess a pivotal role in the aging process (Amorim et al., 2022). A cell’s ability to regulate stress-induced damage of these organelles is crucial for promoting longevity. Mitochondria are not only vital for ATP synthesis but at the same time are controllers of apoptosis and ROS production (Zhang B. et al., 2022; Baev et al., 2022). Excessive mitochondrial activity and energetic burden is likely to result in mitochondrial damage and degradation. The clearance of damaged or unwanted mitochondria by autophagy (also known as mitophagy) is a quality control mechanism thought to play an essential role in cellular homeostasis, metabolism, and development. Effectively managed mitophagy can engender a protective state against long term cellular damage (Guo and Chiang, 2021). Removal of damaged or unwanted mitochondria is therefore essential for cellular stress resilience. It is likely that mitochondria regulate cellular longevity through multiple pathways, including cellular senescence, stem cell function, bioenergetics, inflammation, and the mitochondrial unfolded protein response (mtUPR).

Hypoxic stressors at the cellular level can induce a considerable degree of ROS production that can result in mitochondrial damage (Premont et al., 2000). In this scenario mitophagy serves as the primary mechanism for removing damaged mitochondria to preserve post-mitotic cell viability. The GPCR kinase 2-interacting protein-1 (GIT1) has been shown to be a potent regulator of mitophagy both *in vivo* and *in cellula*. Mitophagy levels in the post-mitotic GIT1 knockout mice neurons are significantly diminished following ischemia and reperfusion cycles. The (virus-mediated) overexpression, however, of GIT1 in this paradigm augmented mitophagy and prevented neuronal apoptosis (Premont et al., 2000). Mitophagic activity stimulated by the fatty acid receptor, GPR120, has been shown to be the mechanism through which DHA (docosahexaenoic acid) exerts protective antioxidant activity in hepatocytes (Chen J. et al., 2021). Neuromedin U (NMU) and its cognate receptor (NMUR2) has been shown to promote the dysfunction of β-cells via induction of mitochondrial failure and ER stress. NMU appears to regulate this insulin-decreasing action through the coherent regulation of multiple proteins involved in mitochondrial fission (Fis1, Drp1), fusion (Mfn1, Mfn2), mitochondrial dynamics (Pgc-1α, Nrf1, Tfam), ER stress (Chop, Atp2a3, Ryr2, Itpr2), and mitophagy (Pink1 - PTEN-induced kinase 1; Park 2 - Parkinson disease 2) (Zhang W. et al., 2021). Mitophagic regulation in a comparable manner has been observed for multiple receptors as well including: the δ-opioid receptor (Xu et al., 2020); the cannabinoid 1 (CB1) receptor (Kataoka et al., 2020); the glucagon-like peptide 1 (GLP-1) receptor (Germano et al., 2020), GPR30 (Wang Y. et al., 2020), and the LRG5 (leucine-rich repeat-containing G protein-coupled receptor 5) receptor (Levy et al., 2020).

### Thermal stress

Cells can experience changes in temperature conditions that can result in either cold or heat stress. Acute cold exposure (Xu et al., 2019) can cause stress and eventually lead to oxidative damage, apoptosis, and other pathophysiological responses (Xu et al., 2017; Tao et al., 2015). In contrast, a greater degree of investigation has been given to the role of heat stress at the cellular level in the context of pathological aging (Zhang H. et al., 2022; Kurop et al., 2021; Gallardo et al., 2021; Trivedi and Jurivich, 2020).

Heat perturbations cause the accumulation of damaged and unfolded proteins as well as aberrant apoptotic mechanisms (Verbeke et al., 2001). Heat stress has been implicated in age-dependent diseases such as neurodegeneration, diabetes, and cancer (Calderwood et al., 2009). One of the most important factors in heat-related stress seems to be transcription factor HSF1 (Heat Shock Factor 1). HSF1 activity appears to be tightly regulated during the aging process. Heat stress responses have been shown to be relatively transient, and one proposed theory is that chronic stress across the lifespan promotes inhibitory pathways designed to attenuate the stress response once the perturbagen diminishes. The generic heat shock response (Calderwood et al., 2009) comprises a conserved program of gene expression to attenuate damage caused by the elevated temperature. This program aims to preserve cytoplasmic proteostasis, protect plasma membranes, and maintain the integrity of organelles through transiently elevated heat shock protein expression (Santoro, 2000). There are four main heat shock transcription factors (HSF1 - 4) – they can potentially create a complex series of interactions with each other through their homo- and heterodimerization and interaction with other chaperoning factors (HSP90, HSP70, HSBP, HSP27) (Calderwood et al., 2009; Sistonen et al., 1994).

Multiple GPCRs have been shown to confer some degree of temperature stress response in cells. These include the: endothelin B (ETB) receptor (Li et al., 2008); neurokinin-3 receptor (Nakamura et al., 2021); lysophosphatidic acid (LPA) receptor (Chei et al., 2020); and PAR-1 receptor (Rada et al., 2021). These receptors demonstrate an intersection with thermal stress response programs via physical associations with some of the key molecular players in this system. In this scenario, GPCRs can regulate thermal stress via functional interactions with heat shock proteins (Rada et al., 2021; Heilman et al., 2020), G protein-coupled receptor kinases (Heilman et al., 2020), and members of the stress-activated protein kinase family (Rada et al., 2021).

## Stress resilience GPCR adaptors

GPCRs play a pivotal role in regulating both the classical hallmarks of aging but also in the creation of a stress resilience system to contend with the diverse stressors that drive the process of pathological aging. Here we hypothesize that part of this stress resilience network may be created by a coherent network of GPCR receptosomes that possess specific stress response management ‘specialties,’ which are encoded into these protein complexes by an associated coterie of specific GPCR adaptor proteins in a specific stoichiometry. In the following will investigate how different GPCR adaptors can entrain a specific spectrum of stress resilience through their dynamic interactions with GPCR signaling complexes.

### Arrestins

The non-visual β-arrestins, β-arrestin-1 (ARRB1) and β-arrestin-2 (ARRB2), are two of the most important and well characterized GPCR adaptors (Maudsley et al., 2015; Leysen et al., 2018; van Gastel et al., 2021; Bohn and McDonald, 2010). Initially, their role was primarily considered as signal attenuating factors for receptor-entrained G protein signals (Miller and Lefkowitz, 2001). Extensive research has demonstrated however that they actually possess a multifunction role in the functional ‘conditioning’ the signaling profile of GPCRs (Maudsley et al., 2013; Pydi et al., 2022; Fuentes et al., 2021).

With respect to aging and stress resilience, β-arrestins have been shown to possess a strong link to pro-aging stress phenotypes via their interaction with stress-activated protein kinases (SAPK) (Zhang W. et al., 2021), members of the DNA damage repair (DDR) processes (Hara et al., 2011), nutrient-sensing cascades (Wang et al., 2016), and signaling systems linked to premature pathological aging (Fu et al., 2019). Illustrating these connections, β-arrestins (perhaps considered the prototypical non-G protein GPCR adaptor) have been shown to control the generation of receptosome complexes (Fu and Xiang, 2015) associated with stress responsiveness, e.g., JIP1 (JNK interacting protein 1 (Musi et al., 2022)), GRP78-ATF4-CHOP of the ISR (Huang et al., 2021), 53BP1 (TP53-binding protein 1) of the DDR (Chadwick et al., 2012a), ASK1 (apoptosis signal regulating kinase 1 (Zhang et al., 2009)), eIF2α of the ERS (Liu et al., 2019) and Nox4 (NADPH oxidase 4 (Theccanat et al., 2016)). Given this extensive level of receptosome complex regulation, it is not surprising that β-arrestins are implicated in multiple forms of stress resilience including oxidative stress resistance (Sharma et al., 2021), thermal stress (Rojanathammanee et al., 2009), autophagic/hypoxic responses (Wang P. et al., 2014), ESR (Tan et al., 2015), and radiation stress/DDR (Nieto et al., 2020). In addition to the potent stress-regulatory role of β-arrestins, the associated α-arrestins are also receiving research interest with respect to their role in managing cellular oxidative stress (Wedegaertner et al., 2022) as well as stress-associated protein kinase (SAPK) pathways (Birch et al., 2021).

### Regulator of G protein signaling proteins

Regulator of G protein signaling (RGS) proteins were originally identified, like β-arrestins, as negative regulators of GPCR signaling via their GTPase-accelerating protein (GAP) activity (Ross and Wilkie, 2000). In this respect RGS proteins were initially defined as proteins that serve as functional conditioners of GPCR signaling at multiple sites within the cell to help add further levels of receptor signaling nuance and thus tailor GPCR signaling to enact a desired complex outcome. Reinforcing the potential of an intracellular sensory GPCR systems, it has been shown that intracellular pools of the heterotrimeric G protein α-subunit Gαi3 can inhibit the activation of JNK and autophagic signaling following nutrient starvation. In this specific context, JNK-mediated phosphorylation of Bcl-2 likely activates autophagic signaling during periods of cellular nutrient deprivation (Bastin et al., 2020). In this paradigm, it has been shown that RGS4 can inhibit Gαi3 activity, which results in the potentiation of Bcl-2 phosphorylation (Bastin et al., 2020). Changes in the subcellular localization of RGS4 (through altered palmitoylation) suggested that Gαi3-regulated Bcl-2 levels and autophagic activity were associated with specific TGN38 (Trans-Golgi network integral membrane protein 2)-labeled vesicle pools. In agreement with this, elevations in Gαi signaling within nutrient-starved adrenal glands from RGS4-knockout mice (compared to wild-type controls) have been shown to result in a dramatic loss of autophagic capacity–a function likely to impact the regulation of proteostasis in the cell.

Increases of RGS2 expression have also been shown to be sensitive to multiple forms of stress. Potentiation of RGS2 expression can enhance translational attenuation through the phosphorylation of the initiation factor eIF2. Under stress-induced translational inhibition, key factors, such as ATF4, can be selectively expressed through a range of alternative translation mechanisms. RGS2 has also been shown to be able to control the regulation of *de novo* protein synthesis through its interaction with the translation initiation factor, eIF2B (Wang and Chidiac, 2019). Hence, RGS2 can significantly increase levels of ATF4 and CHOP, both of which are linked to stress-induced apoptosis and coordination of the ISR (integrated stress response).

While carbohydrates and lipids are the most typical nutrient sources in cells, molecular iron can also be considered an essential nutrient for effective cellular growth and development. RGS19 has recently been shown to regulate cell sensitivity to molecular iron availability (Hwang et al., 2015). In this paradigm, RGS19 appears to be stabilized under iron-depleted conditions, resulting in the activation of growth-inhibitory signals and altered levels of oxidative stress.

Oncogenesis, with its concomitant effects on cellular respiration (i.e., the ‘Warburg Effect’ (Kaczanowski et al., 2018)), can potentially be considered a form of cellular stress. It has been found that RRGS6 can suppress Ras-induced cellular transformation through the Tip60-mediated degradation of Dmnt1 (DNA (cytosine-5)-methyltransferase 1) which results in the promotion of apoptosis (Huang et al., 2014). RGS6 was further demonstrated to diminish cellular transformation in response to oncogenic Ras by downregulating Dnmt1 protein expression. This action then leads to the inhibition of Dnmt1-mediated anti-apoptotic activity. These actions demonstrate that RGS6 acts as an essential cellular defender against oncogenic stress and a potential therapeutic target for developing new cancer treatments.

### GIT2

Mammalian GIT proteins, ARF GTPase-activating protein GIT1 (GIT1) and ARF GTPase-activating protein GIT2 (GIT2), were initially identified as interactors with G protein-coupled receptor kinases (GRKs). GIT1 and GIT2 constitute the GIT protein family. These proteins share a common enzymatic function like GTPase-activating proteins (GAPs) for ADP-ribosylation factor (ARF) small G proteins (Premont et al., 1998; Vitale et al., 2000). ARF proteins have no intrinsic GTPase activity and thus require GAPs to convert the GTP bound to active ARF to GDP causing deactivation. As a result, ArfGAP GIT proteins attenuate ARF protein signaling (Randazzo et al., 1994). Both GIT proteins were originally identified as regulators of GPCR internalization through the influence they exert on the ARF GTP-binding proteins (Vitale et al., 2000; Claing et al., 2000). Given their ability to regulate GPCR endocytosis, it is not surprising that GIT proteins function as active components of GPCR scaffolding complexes to coordinate signaling molecules to distinct subcellular sites of action. Over 100 GIT-associated proteins have been identified through a variety of protein-protein interaction techniques (van Gastel et al., 2018b) – these proteins include: liprin-α; piccolo; insulin receptor substrate 2 (IRS2); and huntingtin (Martin et al., 2016; Hoefen and Berk, 2006). With respect to the physiological roles of GIT proteins, they have been implicated in the regulation of cognitive function. Experimental deletion models have shown that loss of GIT1 induces severe learning and memory deficiencies (Hong and Mah, 2015; Won et al., 2011). In contrast, murine deletion models of GIT2 exhibit anxiety-like behavior and a premature ageing phenotype (Lu et al., 2015; Schmalzigaug et al., 2009). With respect to the pathological aging process both GIT1 (Huang et al., 2020) and GIT2 (van Gastel et al., 2018b) appear to facilitate an ability to connect GPCR receptosomes to stress response preparedness. Differences do however occur between the two GIT proteins with respect to their structural variation. GIT2 demonstrates considerably more structural diversity (due to differential splicing) compared to GIT1, suggesting that it underpins a potential multifunctional role in stress resilience (Premont et al., 2000; Chadwick et al., 2012b). GIT2 has been shown to be extremely sensitive to chronological aging, immunosenescence, DNA damage, pro-diabetic metabolic stressors, and oxidative damage through ROS (Lu et al., 2015; Liguori et al., 2018; Martin et al., 2016; Chadwick et al., 2012b; Siddiqui et al., 2017; van Gastel et al., 2019b). Therefore, GIT2 seems to function as a bridge between multiple forms of aging-related cellular damage and the support of DNA repair/stability, a process crucial to successful and healthy aging (van Gastel et al., 2018b; Lu et al., 2015; Martin et al., 2016; Madabhushi et al., 2014). With respect to DNA damage protection/repair, GIT2 can form micro-complexes with classical DDR proteins such as MRE11, γ-H2AX, and ATM. Through these interactions GIT2 helps coordinate DNA repair by facilitating the regulation and stabilization on the sites of DNA damage of reparative proteins, such as BRCA1, through a poly (ADP-ribose) (PADPR) polymerase (PARP)-linked process (Lu et al., 2015). Several stress-associated GIT2-interacting DDR proteins (ATM, BRCA1, p53) also play important roles in circadian clock regulation (Gery et al., 2006; Miki et al., 2013; Štorcelová et al., 2013). It has been shown by multiple researchers that cellular clock regulation plays a significant role in the aging process as it regulates chronological aging by conferring daily rhythmicity to physiological functions and stress resistance functionality (Takahashi et al., 2008; Bevinakoppamath et al., 2022). In this respect, it appears that an evolutionary synergy exists between clock genes and DDR proteins. Linking clock mechanisms to stress resistance may be one of the most facile mechanisms by which to ‘*educate*’ molecular resilience programs to deal with future expected stressors (Kurhaluk et al., 2021; Liu et al., 2021; Addison et al., 2021).

While circadian rhythms can control cell functionalities over short timescales, cellular functionality is also regulated by molecular programs that need to span years to decades in organisms. Patient-based blood profile analyses have demonstrated that a prominent inflexion in the aging rate occurs before estimated midlife in humans (Belsky et al., 2020). This inflexion point potentially signifies a temporal landmark that allows the differentiation between the patients who will age in a pathological manner or not. A key component of this inflexion is the initiation of the imbalance in efficient glucose usage by the individual. Hence the induction of pre-diabetic states at an early age is a strong predictor of pathological aging. The temporal estimation of this point for a cell/organism is a vital issue to engage effective molecular resilience programs to maximize the probability of healthy aging. Given the importance of this ‘healthspan’ metabolic sensing it is pertinent to note that GIT2 is linked to *i*) the insulinotropic control of energy metabolism and *ii*) its age-related dysfunction both functionally and physically (Martin et al., 2016). Genomic deletion of GIT2 in mice causes a reduction in total pancreatic beta cell mass, alpha cell involution, as well as reduced plasma insulin levels and insulin resistance (Martin et al., 2016). A significantly reduced respiratory exchange ratio (RER) is also observed in GIT2 deletion mice at a relatively youthful age (4 months) indicating that a somatic metabolic switch has occurred. This change in somatic RER is indicative of an organism-wide shift away from glucose as the primary metabolic source towards adipose usage. Hence, the transition from ‘healthy’ to ‘pathological’ aging may coordinate with a significant downshift in RER as nearly all the processes engendered by energy insufficiency result in pathological cell damaging loops (Belikov, 2019; Martin et al., 2016). At the cellular level, the physical association between GIT2 and proteins critical for insulinotropic signaling (e.g., IRS2 (Martin et al., 2016)) is also sensitive to intracellular conditions (generated through diet-induced obesity) indicative of nutrient deprivation and increased ROS levels.

More recently, GIT2 has also been linked with cellular senescence and the creation of the pro-inflammatory senescence-associated secretory phenotype (SASP) (Siddiqui et al., 2017). This study revealed an inter-related group of GIT2-associated factors connected to senescent/DNA stability functionality (Siddiqui et al., 2017). This group of proteins included various high mobility group (HMG) proteins (Lu et al., 2015; Siddiqui et al., 2017), which are stress-sensitive DNA-modulatory factors involved in transcription/translation and DNA repair activities. HMG proteins have also been shown to control GIT2 functions in DDR responses in addition to PARP activity modulation (Lu et al., 2015; Masaoka et al., 2012). For example, HMGB2 is critically involved in regulatory mechanisms involved in DNA damage (Syed et al., 2015) and senescence control (Biniossek et al., 2013), further strengthening this relationship between GIT2 activity and aging-associated cell senescence.

Cellular stress resilience strives to maintain healthy cellular activity in the face of stressors. It is crucial that this cellular activity is translated to eventual organismal health to pursue longevity. In this context, the cellular resilience functions of GIT2 appear to be potently translated to an effective longevity promoting action at the organismal level. In this regard, it has been shown that in female mice GIT2 activity is more biased (compared to males) to preserve healthy glycometabolic activity during lifespan, suggesting that this sex-based dimorphism could be linked with the greater longevity of females compared to males across a large number of species (van Gastel et al., 2019b; Austad and Bartke, 2015; Goyal et al., 2019).

### YAP/TAZ

Given the significant role of the YAP/TAZ system in regulating cellular stress, it is not surprising that these functional GPCR receptosome constituents are now the subject of therapeutic development interest (Strepkos et al., 2022). GPCR-regulation of YAP/TAZ complex activity is especially important with respect to aging and aging-related conditions such as cancer, chronic kidney disease, cardiovascular disease, as well as diabetes and related metabolic conditions (Yeung et al., 2019; Hou et al., 2017; Müller and Schermer, 2020; Ortillon et al., 2021). The YAP/TAZ system potentially defines, like GIT2, a multidimensional factor in the integration of multiple forms of stress sensitivity/response as YAP/TAZ activity has been implicated in autophagic stress responses (Maejima et al., 2022), hypoxia (Gupta and Storey, 2021), thermal stress (Luo M. et al., 2020), nutrient sensation (Kashihara and Sadoshima, 2019), and oxidative stress (Gandhirajan et al., 2016). GPCRs that have been experimentally demonstrated to productively engage the YAP/TAZ system include the PAR-1 (Protease-activated receptor) receptors, sphingosine receptors, and dopamine D1 receptors (Mo et al., 2012; Miller et al., 2012; Lee et al., 2022; Choi et al., 2021). With respect to the importance of stress signaling integration at GPCR receptosomes, recent data has shown that the YAP/TAZ system can be controlled through ET-1 receptors in a manner that is also linked to its ability to interact with the stress-regulating adaptor β-arrestin-1 (Tocci et al., 2021).

### ASK1

Apoptosis signal-regulating kinase 1 (ASK1) can form functional complexes with a variety of factors associated with GPCR receptosomes, e.g., including β-arrestins and multiple members of the stress-associated protein kinase (SAPK) family (*e.g.,* p38 MAPK and JNK) (McDonald et al., 2000; Papaconstantinou, 2019; Hsieh et al., 2010). This association allows for a direct physical interaction with GPCRs. It has been shown that ASK1 signaling complexes link ROS signals generated by dysfunctional mitochondrial electron transport chain complexes to the p38 MAPK stress response pathway (Matsukawa et al., 2004). Hence ASK1 can contribute to the sensitivity and vulnerability of cells to diseases of oxidative stress (Sturchler et al., 2010). Given this functional role of ASK1 complexes, it is unsurprising that they have been shown to associate with the promotion of cell senescence and premature aging in response to oxidative stress and protracted inflammation, especially in the context of age-associated cardiovascular disease.

As nearly all types of rhodopsin-like Class I GPCRs have shown a capacity to interact with β-arrestins, it is conceivable that many GPCR species likely possess a functional capacity to form ASK1-composed signaling complexes during periods of cell stress. Pharmacological intervention is, therefore, feasible through the continued identification of potent, non-toxic, small molecule inhibitors of either ASK1 or p38 MAPK activity (Sturchler et al., 2014; Willoughby and Collins, 2005). Hence, this ASK1 system may represent a near universal GPCR receptosome stress response strategy to mitigate the effects of ROS-mediated pro-aging activities and disorders (Brobey et al., 2015; Lee et al., 2014; Zhang et al., 2016).

## Resilience receptor ensembles

In the previous sections we have defined how multiple components of the aging-stress sensory network are interwoven with a broad variety of GPCR signaling adaptors. It is this linkage that we hypothesize generates a sensory and response molecular signaling lattice that encompasses multiple GPCRs across different sub-cellular compartments. The linkages between the critical factors in the resilience networks are summarized in Table 2.

**TABLE 2:** A broad range of GPCR adaptors are associated with diverse stress response networks. The stress-related functions of some of the major constituents of GPCR receptosomes are detailed, as well as their functional intersections with aging-related biology.

Adaptor	Category/Function	Associated stressors/Responses	Key mechanisms/Interactions	References
ARRESTINS (β-arrestins, ARRB1, ARRB2)	Non-visual arrestins (signal attenuators and adaptors)	Oxidative, thermal, autophagic/hypoxic, ER stress (ERS), radiation/DDR, nutrient-sensing, pro-aging stress phenotypes (e.g., senescence, inflammation)	Forms receptosomes with JIP1 (JNK pathway), GRP78-ATF4-CHOP (ISR), 53BP1 (DDR), ASK1 (apoptosis), eIF2α (translation), Nox4 (ROS); promotes prolonged ERK1/2 signaling, mitophagy, and biased agonism	Chadwick et al. (2012a), Chen et al. (2021a), Mesgarzadeh et al. (2022), Heilman et al. (2020), Fu et al. (2019), Liu et al. (2019)
RGS PROTEINS (RGS2, RGS4, RGS6, RGS19)	Regulators of G protein signaling (GAP activity)	Nutrient starvation/autophagy, translational stress, iron deprivation/oxidative stress, oncogenic/Ras-induced transformation	RGS4 inhibits Gαi3 for Bcl-2 phosphorylation/autophagy; RGS2 enhances eIF2 inhibition/ATF4-CHOP (apoptosis/ISR); RGS19 stabilizes under iron depletion for growth inhibition; RGS6 suppresses Dnmt1 for apoptosis	Ekechukwu and Christian (2022), Bastin et al. (2020), Wang and Chidiac (2019)
GIT2	ARF GTPase-activating protein (endocytosis regulator)	Oxidative/ROS, DNA damage, metabolic/pro-diabetic, immunosenescence, senescence/SASP, chronological aging	Coordinates DDR (MRE11, γ-H2AX, ATM, BRCA1, PARP); links to circadian clock (ATM, p53); insulin signaling (IRS2); HMG proteins for senescence; sex-dimorphic longevity effects; promotes mitophagy post-ischemia	van Gastel et al. (2019a), van Gastel et al. (2018b), Lu et al. (2015), Martin et al. (2016), Huang et al. (2020), Lee et al. (2014)
YAP/TAZ	Transcriptional co-activators (Hippo pathway effectors)	Autophagic, hypoxia, thermal, nutrient-sensing, oxidative stress; linked to cancer, CKD, CVD, diabetes	Regulated by GPCRs (PAR-1, sphingosine, dopamine D1, ET-1 via β-arrestin-1); induces heat shock transcriptome; promotes metabolic reprogramming and inflammation	Chiu et al. (2021), Doan et al. (2021), Mo et al. (2012), Leysen et al. (2022)
ASK1	Apoptosis signal-regulating kinase (MAP kinase activator)	Oxidative/ROS (mitochondrial), inflammation, senescence/premature aging (e.g., CVD)	Scaffolds with β-arrestins/SAPK (p38 MAPK, JNK); links ROS to stress responses; ubiquitin-dependent degradation via β-arrestins attenuates H2O2-apoptosis	Matsukawa et al. (2004), Kimura et al. (2001), Hu et al. (2009), Brobey et al. (2015)

**FIGURE 1:**
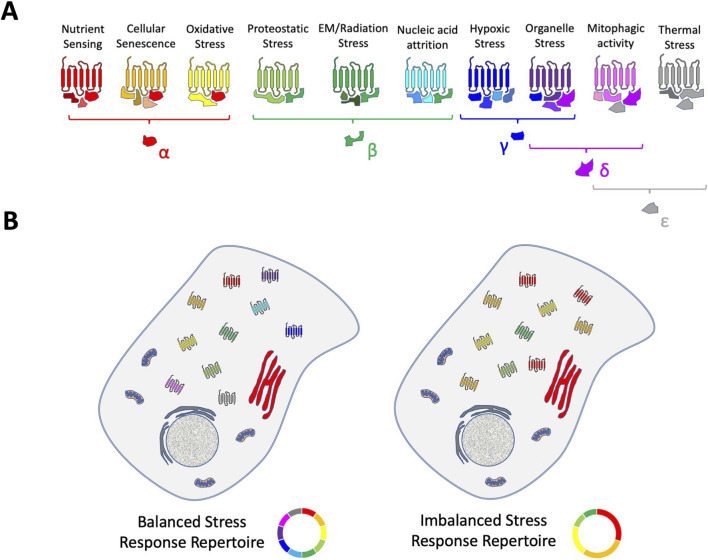
GPCR receptosome composition and repertoire balance can regulate stress resilience. **(A)** GPCR receptosomes can be created through the association of multiple adaptor proteins to engender the creation of distinct GPCR complexes that demonstrate a preference for different forms of stress management activity (coded by different colors in the representation). There are likely to be protein adaptors that can span different stress responsive receptosome structures and these factors (α, β, γ, δ, ε) may represent important keystone factors (e.g., β-arrestin or GIT2) for multidimensional stress resilience. **(B)** The relative levels of intracellular stress resilience GPCR receptosomes in cells can engender either a balanced (left panel) or imbalanced (right panel) stress resistance portfolio. The associated pie charts indicate the relative color-coded stress receptosome balances present in the cells depicted.

**FIGURE 2:**
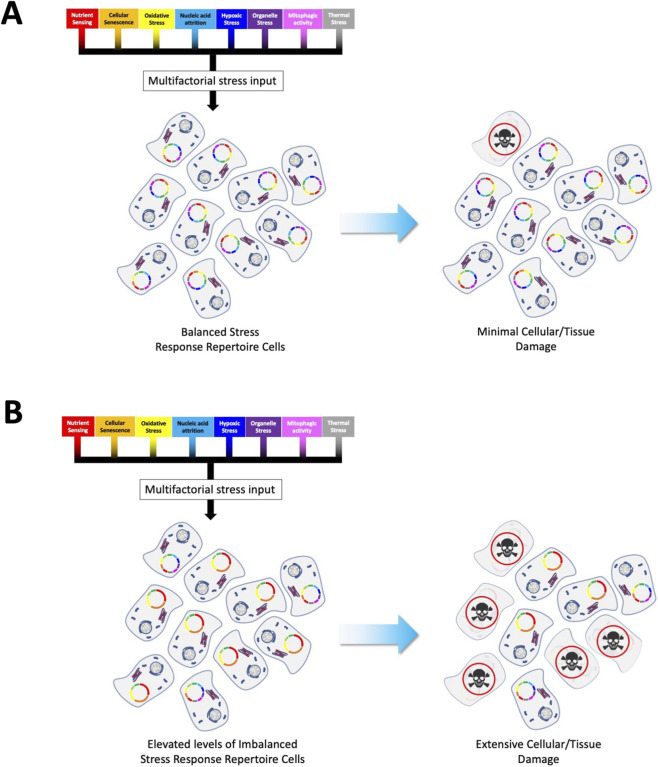
Cellular resilience and survival are a factor of GPCR receptosome expression balance. **(A)** For cells experiencing a variety of stressful insults (color coded as in Figure 1) the prevalence of balanced GPCR resilience receptosomes (indicated by the balanced stress-resistance pie chart depictions in each cell) can allow cells to survive and manage the resultant cellular damage induced by the different input stressors. **(B)** Tissues comprising cells with both balanced and imbalanced repertoires of GPCR resilience (containing either balanced or imbalanced pie chart representations) are likely to suffer more damage when challenged with a variety of stressful inputs as some required stress responsive GPCR receptosomes may be missing in a large number of cells. These cells therefore are more likely to suffer damage and experience death compared to cells possessing a broad spectrum of stress responsive GPCR receptosomes.

**FIGURE 3:**
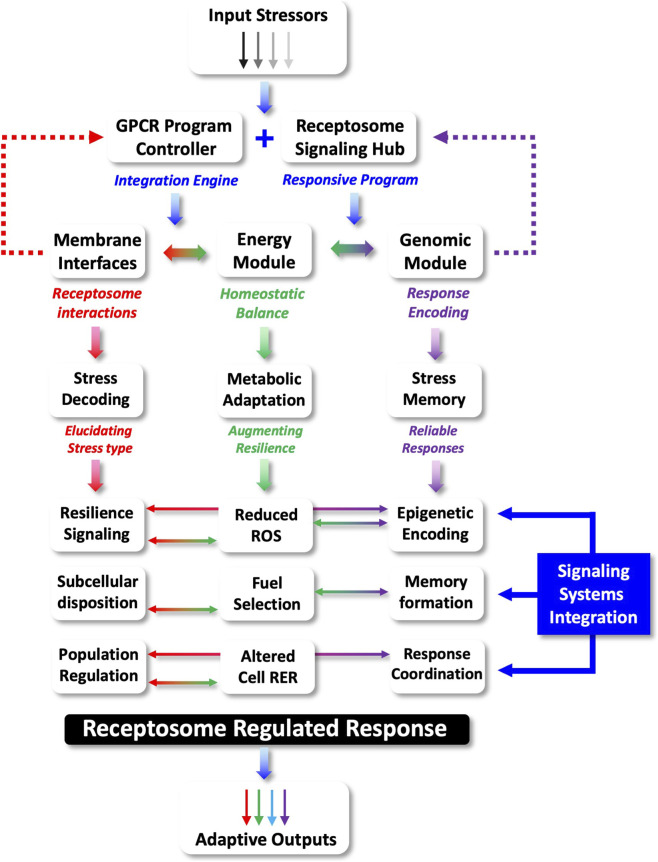
Schematic overview of GPCR receptosome-based signaling network mediating stress decoding, resilience augmentation, and adaptive metabolic outputs. Input stressors activate the GPCR Program Controller and Receptosome Signaling Hub, with the former acting as an effective molecular signaling ‘Integration Engine’ to coordinate responsive programming in concert with the latter that represents the ‘Responsive Program’ element of this system. This stress input drives bidirectional interactions across top-level hierarchical structures including those that control, Membrane Interfaces (GPCR receptosome-mediated), as well as Energy Module (for homeostatic balance and metabolic adaptation), and Genomic Module (for stress memory and response encoding). Downstream outcomes of the processing of the stress inputs include: stress decoding (elucidating stressor type); metabolic adaptation (augmenting resilience via reduced ROS and optimized fuel selection, with resultant changes in cell respiratory exchange ratio [RER]); and stress memory (reliable response formation). These processes feed into resilience signaling (reduced ROS), modified subcellular disposition of receptosomes, and GPCR receptosome population regulation, and coordinated response mechanisms, ultimately generating adaptive outputs through receptosome-regulated integration of signaling systems. Arrows linking factors across the integration networks indicate feed forward as well as feedback (dotted lines) mechanisms. arrows The model illustrates how multidimensional GPCR-receptosome ensembles enable cellular and organismal resilience to diverse stressors.

Based on such accumulated data it is also evident from empirical data that demonstrates that GPCRs can have a critical role in stress response networks (Chadwick and Maudsley, 2010; Leysen et al., 2018; Santos-Otte et al., 2019; Zhou et al., 2015; Naderi Yeganeh et al., 2020), *e.g*., the Relaxin family peptide receptor 3 (RXFP3) (Leysen et al., 2022). As sensors of external cell-to-cell stimuli, it is not surprising that an intracellular sensory/communication network linking stressful stimuli to cellular functions also involves GPCRs. Such a subcellular signaling network likely comprises multiple GPCR receptosomes fine-tuned to detect (on a millisecond-to-millisecond basis) deleterious stressful stimuli, *e.g.,* ROS, nutrient deprivation, protein acetylation/nitrosoation, or temperature stressors. This GPCR stress network may then assist recovery through the deployment of responsive damage limitation and repair mechanisms. The receptor systems involved in this network represent an ensemble of subcellular GPCR receptosomes that strive to maintain a flexible and optimal range of sensitivities to a wide variety of stressors. This GPCR network is challenged with the need to regulate and control cell damage as the presence of molecular stress is a constant–but random–fact of lifespan. Hence, this network is required to maintain an effective stress vigilance from milliseconds to years. In this context, cells that maintain the deployment of a broad spectrum of stress-resistant GPCR complexes are the most likely to survive these perturbations over both short and extended periods of time (Figures 1, 2). To reinforce continued healthy functionality, cells potentially have prioritized a capacity to predict the arrival of rapid stressful insults. It is feasible that this stress-predictive process may be controlled through GPCR systems that can sense the earliest signs (from multiple input pathways) of stress. In response to this, they then may coordinate rapid response transcriptional/translational networks to ensure that further specific sensory and reactive GPCR receptosome complexes are created to content with this pattern of stressful input (Figure 3). In this respect, it is therefore critical that a cell maintains a potent capacity to generate and coordinate the synthesis of proteins needed to construct the most effective receptor ensembles. The most effective stress-response GPCR ensembles will then facilitate rapid and reversible responses to the most likely range of insults that a specific cell receives. It is conceivable that these GPCR adaptors may be the most critical features of the cellular resilience network as they help condition the sensitivity to and capacity to respond to the cell stress events. With this proposal, a dilemma exists for GPCR sensory systems within a cell. Hence, how best to deploy the energetic and proteomic resources it possesses. There are several theoretical considerations that a stress sensory network requires: *a*) which type of stressful perturbations are most likely to occur? *b*) Are there adaptor protein-GPCR relationships that create a more efficient spectrum of resistance capacities compared to others? *c*) Are there GPCR-interacting factors that can be employed to bridge multiple stress response pathways? Given these issues, it is important to appreciate that both GPCR sensory network and stressors exist in two distinct temporal realms, *i.e.,* stressors may appear at a micro/milli-second time frame while reactive sensation and response of GPCR resilience ensembles may take hours/days to respond to the stressor. Given this disparity it is evident that effective stressor prediction (potentially informed by stress monitoring systems that identify features of failing energy metabolism or nutrient uptake) would enable the cell to not squander precious resources, and create receptor ensembles unsuited to the impinging stressors (Liu et al., 2015). In addition to this, if a cell experiences a range of stressful inputs, then prioritizing the generation and distribution of protein factors that facilitate responses between one stressor, or another may represent an important pro-survival locus for future investigation (Figure 2). Our current research with the GPCR adaptor GIT2 potentially underlines this issue as GIT2 appears to possess a bridging capacity between diverse stressful stimuli, *e.g.,* metabolic dysfunction (Martin et al., 2016; Chadwick et al., 2012b), ROS (Martin et al., 2016), and DNA damage (van Gastel et al., 2019a; Lu et al., 2015). Cells may therefore prioritize the creation of GIT2-associated receptosomes in times of multiplexed stress input to prevent accumulated cellular damage that may promote age-related disease signature generation and pathological maturation (van Gastel et al., 2018b).

The relationship between stressors and the GPCR-based response network could represent a classical ‘Game’ in Game Theory, which studies strategic interactions between rational decision-makers (Myerson, 1991). Game Theory models have analyzed biological phenomena from species competition (Smith and Price, 1973) to neural network communication (Christodoulou et al., 2010). Originally developed for zero-sum competitions where one factor succeeds at another’s expense (Smith, 1981), Game Theory simplifies dynamic equilibria in complex systems like GPCR-ligand and sensory networks (Del Castillo and Katz, 1957), including protein-protein interactome flexibility, ion release, or receptor state conversion (Maudsley et al., 2005; Martin et al., 2009a; Maudsley et al., 2012). In stress protection, a key molecular game pits the cell’s GPCR stress receptosome ensemble against damaging stressors, requiring predictive ‘moves’ from both sides. Analogous to adaptable chess openings (e.g., Reti Opening (Davies, 2004)), receptosome flexibility compensates for time disadvantages against rapid stressors, emphasizing early stressor sensing. Transcriptional machinery, linked to non-G protein GPCR signaling (Gesty-Palmer et al., 2013; Luttrell et al., 2015), may enhance this rapid response over protein turnover. This creates a ‘receptosome dilemma’ akin to the Prisoner’s Dilemma, where receptosome gambits must favor cell survival. We hypothesize stress resilience as a ‘game’ between stressors and receptosomes, with keystone adaptors like GIT2 enabling flexible ‘moves’ (e.g., bridging ROS/DDR) for supremacy in multiplexed stress. Cell survival primacy has refined this receptosome network (Chadwick and Maudsley, 2010), as failed stress-response gambits lead to viability loss. In multicellular evolution, pre-pathology stress responses are highly successful (Belsky et al., 2020), but aging alters GIT2 expression/function (van Gastel et al., 2018b), potentially suboptimal post-metabolic aging inflexion due to complex stressor ‘attacks’ (van Gastel et al., 2019a; Martin et al., 2016; van Gastel et al., 2019b; Chadwick et al., 2010b). GPCR systems compete to maintain neurotransmission, endocrine axes, and sensory mechanisms, evoking persistent homology in biological networks (Hendrickx et al., 2020). Thus, GPCR control over homeostasis/allostasis is innate and vital for longevity, suggesting therapies mimic endogenous multilayered patterns. Recent research applies Game Theory to network theory for ‘Precision Medicines’ (Biane et al., 2016). Biane et al. combined Game Theory and Boolean networks in a ‘network action game’ to optimize drug selection for breast cancer, modeling disease-drug interplay and matching curated literature. Disease-drug strategies often target graph network edges in protein interactomes, relying on refined signaling paradigms for tractable remediation. Farahmand et al. used a Game Theoretic Approach (GTA) with expression profiles and interaction networks to identify breast cancer sub-networks, yielding novel metastatic markers, susceptibility genes, and superior classification (Farahmand et al., 2016). Game Theory also aids peptidergic anti-cancer drug identification (Ge et al., 2019). Chaos Game Representation (CGR), converting sequences to graphical forms via iterated function systems (Farahmand et al., 2016), combined with machine learning (e.g., support vector machines, deep learning), analyzes peptidergic sequences (Ge et al., 2019; Löchel et al., 2020). Ge et al. (2019) advanced CGR for superior anti-cancer peptide prediction using chemoinformatic databases (Ge et al., 2019). These Game Theory advances hold emerging relevance to GPCR biology.

## Homeostasis and allostasis within networks–the role of GPCRs

GPCR sensory networks can potentially be controlled with respect to both long and short-term stressful events at a single cell level. Complex biological events, at the network level, demonstrate two distinct types of *gestalt* activity, *i.e.,* homeostasis (long term global regulation) and allostasis (short term local network maintenance, *e.g.,* ‘*dynamic vertex’* re-arrangement in a standard graph network). Multiple concepts of allostasis are derived from the work of Sterling and Eyer (1988). They defined allostasis using the following description: ‘*an organism must vary all the parameters of its internal milieu and match them appropriately to environmental demands*.’ In the context of health network disruption, characterized by the persistence of perceived (*e.g.,* disruption of effective glucose metabolism) or actual (oxygen radical molecular damage) insults, the cellular stress response network needs to maintain a consistent vigilance and cell health ‘self-checking’ activity. This ongoing allostatic activity may involve considerable energetic (*i.e*., ATP-consuming) behavior and thus presents a significant metabolic burden itself. For example, such elevated cellular activity can be analogized with the observed elevations of default network activity in the brain that is linked to increased risks for neurodegeneration (Grieder et al., 2018). Hence, the dynamic plasticity of allostatic physiological signaling networks through a rapid response to stressors may likely facilitate the maintenance of global longer-term homeostasis. Potential failures in this multilayered (allostasis and homeostasis) paradigm may indeed represent the first foothold of disease signatures within the network (Leslie and Vartak, 2020; Jestin et al., 2020). Hence loss of this damage-sensing/responding system may also be considered one of the major triggers of disease phenotype generation.

In situations of cellular stress, molecular allostasis could ensure stability through perturbagen-induced change by modifying the setpoints and parameters of feedback control systems (McEwen, 1998). Predictive, anticipatory, stress response behavior has recently been shown with respect to lifespan regulation and oxidative burdens in a gender-distinct manner (van Gastel et al., 2019b). Despite being a basically beneficial systemic process, allostasis may also expose the cell to a new kind of frailty, often referred to as ‘*allostatic load.’* This allostatic load can represent such a significant energetic burden so that there may result in a critical loss of cellular viability. To help understand how such burdens of stress-and anticipatory allostasis can disturb stress response networks, a recent modification to Sterling’s original concepts has been introduced by Lee (2019), *i.e.,* the Paradigm of Allostatic Orchestration (PAO). The PAO represents a conceptual understanding of how neural (brain) inputs into the homeostatic network can facilitate the creation of an active allostatic state. Similarly, the concept of ‘interoception’ conceptualizes the sequelae of the reverse direction of this information stream, *i.e.,* the psychosocial/cognitive effects of physiological homeostasis (Yoris et al., 2020). Lee proposed that an ‘*allostatic state*’ represents a neutrally focused monitoring mechanism that upon global network integration generates the entity of somatic homeostasis (Lee, 2019). The nature and efficiency of somatic allostasis has been proposed to underpin multiple deleterious conditions associated with ongoing stressful perturbations/insults such as: chronic pain (Burke et al., 2017); immune dysfunction (Glaser and Kiecolt-Glaser, 2005); irritable bowel syndrome (Mayer et al., 2001); and stimulant addiction (Koob and Schulkin, 2019). The allostatic state proposed in the PAO does not apply to pathology or disease alone, and neither does it suggest that controlling neural effects (or stressors) are the sole cause of any disorder. Instead, the PAO serves to reinforce (like interoception) that there is always bidirectional influence between any given system expression and the functionality of network controlling features. The PAO also addresses the generation of the potential damage-generating loci within systemic networks, *i.e*., allostatic load. The need to maintain this network surveillance is likely to generate significant energetic stress upon any cellular network and thus could be key to its gradual dysfunction over lifespan. This concept within PAO has been referred to as ‘optimal anticipatory oscillations.’ This aspect of the PAO is similar in intent and meaning to Sterling’s definition of health as ‘optimal predictive fluctuation’ (Lee, 2019). Optimal anticipatory oscillation builds on an appreciation of network controlling systems as “*prediction machines*” (Engel et al., 2001), and reflects the capacity for a matching between operations in the cellular network with the typically oscillatory molecular features of cells. Thus, in the context of the whole organism, these prediction machines reduce limitations on cell activity, enhance resilience, and also expand the range of possible functionalities or opportunities in which cell physiology can expand to contend with the impinging stress. For example, in respect of cardiovascular activity, optimal anticipatory oscillation may be demonstrated as heart rate variability. High heart rate variability is indicative of a capacity for rapid recalibrations of cardiac output in context-sensitive ways that result in eventual decreased risk of morbidity or mortality (Dekker et al., 1997). In a similar manner, research has demonstrated that continuous glucose monitoring (CGM) humans often display considerable diversity (often referred to as patient-specific ‘*glucotypes*’) in their ability to modulate elevations in post-prandial glucose levels (Hall et al., 2018). These diverse *glucotypes* may also represent distinct protective or resilient states of the patients to the systemic glucose perturbations. Innate optimal anticipatory oscillation may also be associated with characteristic sleep patterns (Buysse, 2014), motor behaviors or sensory acuity (McClintock et al., 2016), or positive cognitive appraisals (Kalisch et al., 2015). It is likely that circadian clock regulation underpins many of these network influencers, which again reinforces the importance of molecular aging pathways in disease etiology as both circadian clock proteins and DNA damage repair (DDR) proteins have co-evolved in a coherent manner (Hendrickx et al., 2020; Mazzoccoli et al., 2016). The synergy between these two short-loop allostatic systems - circadian clock and DDR - provides a form of cellular damage feedback within the lens of time perception that can then further inform our allostatic appreciation of the human health network. In this specific example, a molecular characterization of the allostatic state components of an optimal anticipatory oscillation may allow the creation of novel therapeutics that reduce the prevalence and incidence of morbidity through the augmentation of systemic allostatic resilience. Our recent research linking the aging controller, GIT2, to systemic cellular resilience via its coordination of DDR, oxidative stress resistance, and circadian clock control via its specific functional engagement of the RXFP3 receptor, provides a compelling novel route of systems-level drug development (Fagerberg et al., 2014; Leysen et al., 2018). With regards to this RXFP3-GIT2 system, it appears that this small-scale allostatic sensory network maintains a functional surveillance of potential stress/damage through a process balancing energy source usage, oxidative radical management, and DNA repair (van Gastel et al., 2019a; Lu et al., 2015; Martin et al., 2016). This and similar investigations in the future will likely demonstrate the benefit of therapeutics targeted towards the points of allostatic control and integration within the health network as opposed to traditional therapeutics that aim to primarily attenuate disease symptoms.

## Disease signatures at the subcellular level

The ability to identify and classify disease at a molecular/cellular level before the development of perceptible symptoms will offer an important capacity for novel prophylactics. Cellular health and dysfunction can be considered as simply different protein network states. Hence an agent, which could alter a disease network signature at an early stage, may potentially be described as a ‘*trajectory modulator,’ (in the context of disease)* as opposed to a remedial therapeutic*.* To develop these trajectory modulators, it is important that effective technologies for the identification and characterization of such molecular disease signatures at the single cell level are developed. Early-stage identification of characteristic disease signatures is vital because in these initial stages there will probably be minimal cellular pathology masking this early disease molecular signature–hence making it more clearly defined. In contrasts, in the later stages of the disorder there will likely be many other pathophysiological network perturbations - due to cellular degradation - rather than the specific disease etiology. In such cases, the accumulated pathophysiological changes may diminish the clarity and ability to define the extant early disease signature. In addition to this, the network perturbations at an early stage of disease etiology are likely to be of a smaller magnitude, thus making their reversal possible with perhaps only a modest efficacy of a targeted therapeutic agent. The *persistent homology* of the stress response protein network structure is likely to be preserved across multiple magnitude scales. In this regard, techniques such as Topological Data Analysis (TDA) (Hendrickx et al., 2020; Brain Health Modeling Initiative BHMI, 2018; Martin et al., 2013) can be deployed to reveal the crucial characteristics of a well-developed and complex disease at an early temporal stage when only small magnitude protein signaling perturbations may exist. The exploitation of accurate and reliable high-dimensionality data is crucial for dimensional condensation approaches such as TDA (van Gastel et al., 2019b). The effective integration of this data from various experimental streams (RNA sequencing, proteomic, metabolomic) will be necessary as both cellular dysfunction and disease signatures likely exist at the multiscale level (van Gastel et al., 2019a). Diagnostic and therapeutic molecular disease signatures for many different conditions have been proposed, *e.g.,* diabetes mellitus (Mazzoccoli et al., 2016), sporadic inclusion body myositis (De Ridder et al., 2020), breast and prostate cancer (Bahmad et al., 2020), pathological aging (Bakula et al., 2018) and immunosenescence (Siddiqui et al., 2017). The ability to accurately measure defining cellular dysfunctions and predict the future phenotype and outcomes of various treatment options is necessary for the future success of tailored therapies for the unique molecular disease networks of individual patients (Hendrickx et al., 2020). Studies have recently reported important developments in informatic deconvolution approaches, related to the derivation of multifactorial disease signatures (Ching et al., 2018; Smith et al., 2020). Given that individual cellular responses to impinging stressors will subsequently spread to neighboring cells (Olsson et al., 2010) – further increasing the potential complexity of molecular models needed - it is evident that a more nuanced understanding of how disease signatures are created from single cellular perturbation responses will be vital for the future development of ‘*trajectory modifiers’* for aging-associated disorders.

## Therapeutic design for stress resilience

Traditionally, pharmacological compounds are identified and prioritized based on their biological effect through modulation of the activity of a unitary target, *e.g.,* a specific enzyme, the gating of an ion channel, or the stimulation of a certain receptor. This unitary target focusing does not consider the likelihood that cellular and somatic functions are the result of a myriad of interconnected signaling systems. The routine implementation of high-dimensionality data acquisition and analysis has now made it possible to not only understand the complexity of systemic diseases, but also to understand the possibility of inter-individual variation in etiology of diseases. This diversity in disease mechanisms can be specifically addressed with what is termed ‘precision,’ ‘personalized,’ or ‘individualized’ medicine. Precision medicines (Sankar and Parker, 2017; Allegaert et al., 2020) possess tailored efficacy profiles, which are selected to match best with the specific disease profile of the patient. In this developing clinical model, computational pharmacology plays an important role for assisting physicians in their decision making (Wang JX. et al., 2020) and stratification (De Ridder et al., 2020) by combining data analysis and systems biology modeling. In the field of precision medicine, the assessment of drug efficiency necessitates an ability to identify the various disturbances in a healthy signaling system network, at both the somatic and single cellular level, which are caused by a disease process. In previous sections, we have discussed that with respect to subcellular stress responses, GPCRs are among the most tractable therapeutic systems to control individual variations in these resilience networks. Therefore, in the context of precision medicine, we should consider which strategies to implement for drug development. Here there is a clear dichotomy, either the filed can adopt the highly inefficient drug design for individual patients or enable and support drug design for groups of patients rationally clustered together in a specific manner that is already tractable to GPCR therapy (Bilkey et al., 2019). The curation of known signaling paradigms and their enrichment across the specific networks of diverse patient groups can help to achieve this second option. First, the rapid and accurate high-dimensionality data profiling of individual patients is crucial. Specific GPCR-sensitive signaling cascades can then be extracted out of these datasets (Maudsley et al., 2016). As noted previously, we already mentioned a problem with such a strategy, namely, the diversity of signaling at the level of a single GPCR unit due to their pluridimensional signaling profiles. Therefore, it can be argued that distinct patterns of GPCR adaptor coupling - engineered to create a diverse response range to cellular stressors - could be associated with different sub-forms of diseases or even different populations of patients. Extensive acquisition of high-dimensionality data from numerous patients will be necessary to confirm this. Existing GPCR-based therapies have already shown a clear capacity to facilitate multiple drug interventions for distinct patient populations, via their ability to differentially control distinct patterns of downstream signaling. GPCR-based agents exhibiting selectivity for signaling via GIT2 (van Gastel et al., 2019a), β-arrestin (van Gastel et al., 2018a; Maudsley et al., 2015; Maudsley et al., 2016), or NHERF (Saponaro et al., 2018), may prove to be differentially effective in various patient groups of which the pathology is related to such signaling dissimilarities. A single GPCR target can functionally interact with multiple downstream signaling adaptors in many distinct cell background situations within an individual. Therefore, these transmembrane receptors provide a unique opportunity to produce highly tailored efficacy profiles, specific for biological processes, tissues, patients, and disease clusters (Maudsley et al., 2004; Maudsley et al., 2005; Ilter et al., 2019). Recently it has also been shown that the regions encoding GPCRs are often specific loci for disease initiation via somatic mutations that will also likely exist in a patient cluster-based manner (Fukami et al., 2018; Zhao et al., 2019). These features of GPCR biology should therefore drive the pioneering of GPCR-based therapies with pluridimensional efficacy, both within a classical cell surface and subcellular stress responsive manner, as perhaps one of the most convenient and potentially effective forms of precision medicine (Maudsley et al., 2004; Maudsley et al., 2015; Gesty-Palmer et al., 2013).

Given the GPCR signaling complexity associated with the generation of distinct receptosome species, multiple receptor targets are created *de facto* through the structural modification of the receptor’s heptahelical core by its association with adaptors. In this scenario, the appreciation of ligand cognicity is a crucial issue. Cognate (orthosteric) GPCR ligands attempt to impact every consequence of receptor activation in the same manner, whether desensitization, internalization, trafficking, or G protein coupling (Maudsley et al., 2005; Maudsley et al., 2012). Hence, cognate ligands strive to engender an omnipotent and finely balanced efficacy profile. The ability of a ligand to achieve this ‘optimal balanced’ role at a systemic, multi-tissue level is highly unlikely due to tissue-to-tissue variation in receptor and signaling adaptor expression across the diverse cellular conditions (Maudsley et al., 2004; Marti-Solano et al., 2020; Stoeber et al., 2018).

An effective definition of a cognate ligand for a specific GPCR could be represented by the ability of the specific ligand to ‘*most equally stimulate the greatest number of potential signaling outcomes for the specific GPCR across a diverse series of cellular settings.’* In this paradigm, receptors and their cognate ligands would have co-evolved to generate the most physiologically adaptable and effective responses in target cells. With respect to the concept of functional relationships between ‘cognate’ ligands and their preferred receptors, the aging/stress response paradigm presents an important pathophysiological process that represents perhaps the largest systemic and coordinated alteration of cellular signaling in physiology. In addition to redefining ‘cognicity,’ it is vital to redefine the ‘specificity’ of ligand-induced efficacy profiles (Stoeber et al., 2018; Alewijnse et al., 2000; Pauwels et al., 2000). Within the framework of multiple active and signaling states, the unlikely nature of perfectly balanced agonism leads to the near ubiquity of the concept of ‘biased agonism.’ Appreciating the likelihood that nearly all GPCR ligands are pluridimensionally biased will help to develop functionally selective drugs, which activate beneficial downstream pathways and suppress adverse side effects (Anckaerts et al., 2019). While this discovery represented a paradigm shift in GPCR signaling, it is now to be placed in the context of the pluridimensional spectrum of GPCR signaling (Silva et al., 2019). Recent advances in biased agonism highlight ligands and molecular signaling systems favoring adaptor paths for stress resilience, maintenance of anti-inflammatory pathways, psychological stress, and modulation of glutamatergic hyperactivity pathways (Mantas et al., 2022; Zheng and Xu, 2025; Tiwari et al., 2021; Nestler and Russo, 2024). It is therefore evident that the exploitation of GPCR signaling diversity is revealing further benefits of selectively exploiting signaling bias.

An in-depth understanding of the G protein-coupling capacity of GPCRs has been transformative for the creation of GPCR-based therapeutics. This G protein-centric focus of GPCR signaling was expanded by the discovery that β-arrestins–originally conceptualized as simple terminators of G protein signaling (Ferguson et al., 1996) – can also act as productive signaling adaptors (Luttrell et al., 1999). Subsequent studies from many different researchers have demonstrated that the domain of GPCR signaling is significantly more complex and diverse than initially imagined (Maudsley et al., 2021; Maudsley et al., 2005; van Gastel et al., 2021). In new multistate signaling GPCR models, specific agonists possess the capacity to interact with and stabilize distinct active receptosomes by revealing different intracellular regions involved in coupling separate G protein types, initially demonstrated by the β2-adrenergic receptor antagonist ICI-118-551 (Gong et al., 2002) and β-arrestin signaling (Gesty-Palmer et al., 2013; Cong et al., 2013). It is becoming increasingly clear that agonist-selective receptor signaling that targets a discrete subset of the possible response profiles presents an opportunity to develop precision therapeutics with selective enhanced efficacy profiles.

## Conclusion

GPCRs were traditionally viewed as signal transducers that mediate communication by transferring molecular signals between cells or tissues via external ligands. However, emerging evidence suggests that these versatile transmembrane proteins also play a crucial role in sensing and responding to intracellular stress. The remarkable molecular diversity of GPCRs enables the formation of complex receptor networks, or “receptosomes,” that help maintain healthy cellular function even when faced with pathological disruptions, many of which contribute to the aging process. New insights into endogenous stress-memory mechanisms, particularly those related to energy metabolism and mitochondrial function, highlight protective strategies that mitigateaging-related pathology (Zhang Q. et al., 2021). The intersection of GPCR networks with mitochondrial processes offers deeper understanding of stress-sensitive GPCR functions. Considering the significant impact pathological aging has on disease development, it is critical to conceptualize and develop therapeutic strategies targeting this stress-response network. Strengthening these networks may slow the accumulation of damage over a lifetime, potentially reducing the prevalence and severity of aging-related diseases.

## References

[B1] Adams J. W. Wang J. Davis J. R. Liaw C. Gaidarov I. Gatlin J. (2008). Myocardial expression, signaling, and function of GPR22: a protective role for an orphan G protein-coupled receptor. Am. J. Physiol. Heart Circ. Physiol. 295 (2), H509–521. 10.1152/ajpheart.00368.2008 18539757

[B2] Addison R. Weatherhead S. C. Pawitri A. Smith G. R. Rider A. Grantham H. J. (2021). Therapeutic wavelengths of ultraviolet B radiation activate apoptotic, circadian rhythm, redox signalling and key canonical pathways in psoriatic epidermis. Redox Biol. 41, 101924. 10.1016/j.redox.2021.101924 33812333 PMC8050411

[B3] Al M. S. Malik S. S. Al I. M. Haji E. Dairi G. Mohammad S. (2022). Free Fatty Acid Receptors (FFARs) in Adipose: Physiological Role and Therapeutic Outlook. Cells 11 (4), 750. 10.3390/cells11040750 35203397 PMC8870169

[B4] Aldossary H. S. Alzahrani A. A. Nathanael D. Alhuthail E. A. Ray C. J. Batis N. (2020). G-Protein-Coupled Receptor (GPCR) Signaling in the Carotid Body: Roles in Hypoxia and Cardiovascular and Respiratory Disease. Int. J. Mol. Sci. 21 (17), 6012. 10.3390/ijms21176012 32825527 PMC7503665

[B5] Alewijnse A. E. Timmerman H. Jacobs E. H. Smit M. J. Roovers E. Cotecchia S. (2000). The effect of mutations in the DRY motif on the constitutive activity and structural instability of the histamine H(2) receptor. Mol. Pharmacol. 57 (5), 890. 10.1016/S0026-895X(24)26497-X 10779371

[B6] Allegaert K. Smits A. van Donge T. van den Anker J. Sarafidis K. Levtchenko E. (2020). Renal Precision Medicine in Neonates and Acute Kidney Injury: How to Convert a Cloud of Creatinine Observations to Support Clinical Decisions. Front. Pediatr. 8, 366. 10.3389/fped.2020.00366 32850523 PMC7399072

[B7] Ambekar S. S. Hattur S. S. Bule P. B. (2017). DNA: Damage and Repair Mechanisms in Humans. Glob. J. Pharm. Pharm. 3, 555613. 10.19080/GJPPS.2017.03.555613

[B8] Amorim J. A. Coppotelli G. Rolo A. P. Palmeira C. M. Ross J. M. Sinclair D. A. (2022). Mitochondrial and metabolic dysfunction in ageing and age-related diseases. Nat. Rev. Endocrinol. 18 (4), 243–258. 10.1038/s41574-021-00626-7 35145250 PMC9059418

[B9] Anckaerts C. van Gastel J. Leysen V. Hinz R. Azmi A. Simoens P. (2019). Image-guided phenotyping of ovariectomized mice: altered functional connectivity, cognition, myelination, and dopaminergic functionality. Neurobiol. Aging 74, 77–89. 10.1016/j.neurobiolaging.2018.10.012 30439596

[B10] Ardura J. A. Alonso V. Esbrit P. Friedman P. A. (2017). Oxidation inhibits PTH receptor signaling and trafficking. Biochem. Biophys. Res. Commun. 482 (4), 1019–1024. 10.1016/j.bbrc.2016.11.150 27908723 PMC5245921

[B11] Ariza A. C. Deen P. M. Robben J. H. (2012). The succinate receptor as a novel therapeutic target for oxidative and metabolic stress-related conditions. Front. Endocrinol. (Lausanne) 3, 22. 10.3389/fendo.2012.00022 22649411 PMC3355999

[B12] Aubert G. Lansdorp P. M. (2008). Telomeres and aging. Physiol. Rev. 88 (2), 557–579. 10.1152/physrev.00026.2007 18391173

[B13] Austad S. N. Bartke A. (2015). Sex Differences in Longevity and in Responses to Anti-Aging Interventions: A Mini-Review. Gerontology 62 (1), 40–46. 10.1159/000381472 25968226

[B14] Azeloglu E. U. Iyengar R. (2015). Signaling networks: information flow, computation, and decision making. Cold Spring Harb. Perspect. Biol. 7, a005934. 10.1101/cshperspect.a005934 25833842 PMC4382748

[B15] Baev A. Y. Vinokurov A. Y. Novikova I. N. Dremin V. V. Potapova E. V. Abramov A. Y. (2022). Interaction of Mitochondrial Calcium and ROS in Neurodegeneration. Cells 11 (4), 706. 10.3390/cells11040706 35203354 PMC8869783

[B16] Bahmad H. F. Peng W. Zhu R. Ballout F. Monzer A. Elajami M. K. (2020). Protein Expression Analysis of an *In Vitro* Murine Model of Prostate Cancer Progression: Towards Identification of High-Potential Therapeutic Targets. J. Pers. Med. 10 (3), 83. 10.3390/jpm10030083 32784957 PMC7565308

[B17] Bakula D. Aliper A. M. Mamoshina P. Petr M. A. Teklu A. Baur J. A. (2018). Aging and drug discovery. Aging (Albany NY) 10 (11), 3079–3088. 10.18632/aging.101646 30425188 PMC6286828

[B18] Balogh A. Shimizu Y. Lee S. C. Norman D. D. Gangwar R. Bavaria M. (2015). The autotaxin-LPA2 GPCR axis is modulated by γ-irradiation and facilitates DNA damage repair. Cell Signal 27 (9), 1751–1762. 10.1016/j.cellsig.2015.05.015 26027517 PMC4514920

[B19] Bar R. S. Levis W. R. Rechler M. M. Harrison L. C. Siebert C. Podskalny J. (1978). Extreme insulin resistance in ataxia telangiectasia: defect in affinity of insulin receptors. N. Engl. J. Med. 298 (21), 1164–1171. 10.1056/NEJM197805252982103 651946

[B20] Barabasi A. L. Albert R. (1999). Emergence of scaling in random networks. Science 286 (5439), 509–512. 10.1126/science.286.5439.509 10521342

[B21] Barnes R. P. Fouquerel E. Opresko P. L. (2019). The impact of oxidative DNA damage and stress on telomere homeostasis. Mech. Ageing Dev. 177, 37–45. 10.1016/j.mad.2018.03.013 29604323 PMC6162185

[B22] Basha F. H. Hemalatha S. (2022). Screening the Efficacy of Melatonin on Neurodegeneration Mediated by Endoplasmic Reticulum Stress, Inflammation, and Oxidative Damage. Appl. Biochem. Biotechnol. 194 (3), 1105–1119. 10.1007/s12010-022-03814-x 35015217

[B23] Bastin G. Dissanayake K. Langburt D. Tam A. L. C. Lee S. H. Lachhar K. (2020). RGS4 controls Gαi3-mediated regulation of Bcl-2 phosphorylation on TGN38-containing intracellular membranes. J. Cell Sci. 133 (12), jcs241034. 10.1242/jcs.241034 32501280

[B24] Belikov A. V. (2019). Age-related diseases as vicious cycles. Ageing Res. Rev. 49, 11–26. 10.1016/j.arr.2018.11.002 30458244

[B25] Belsky D. W. Caspi A. Arseneault L. Baccarelli A. Corcoran D. L. Gao X. (2020). Quantification of the pace of biological aging in humans through a blood test, the DunedinPoAm DNA methylation algorithm. Elife 9, e54870. 10.7554/eLife.54870 32367804 PMC7282814

[B26] Beltrán González A. N. López Pazos M. I. Calvo D. J. (2020). Reactive Oxygen Species in the Regulation of the GABA Mediated Inhibitory Neurotransmission. Neuroscience 439, 137–145. 10.1016/j.neuroscience.2019.05.064 31200105

[B27] Bertalanffy L. V. (1972). The History and Status of General Systems Theory. Acad. Manag. J. 15, 407–426. 10.5465/255139

[B28] Bettedi L. Foukas L. C. (2017). Growth factor, energy and nutrient sensing signalling pathways in metabolic ageing. Biogerontology 18 (6), 913–929. 10.1007/s10522-017-9724-6 28795262 PMC5684302

[B29] Bevinakoppamath S. Ramachandra S. C. Yadav A. K. Basavaraj V. Vishwanath P. Prashant A. (2022). Understanding the Emerging Link Between Circadian Rhythm, Nrf2 Pathway, and Breast Cancer to Overcome Drug Resistance. Front. Pharmacol. 12, 719631. 10.3389/fphar.2021.719631 35126099 PMC8807567

[B30] Biane C. Delaplace F. Klaudel H. (2016). Networks and games for precision medicine. Biosystems 150, 52–60. 10.1016/j.biosystems.2016.08.006 27543134

[B31] Bilkey G. A. Burns B. L. Coles E. P. Mahede T. Baynam G. Nowak K. J. (2019). Optimizing Precision Medicine for Public Health. Front. Public Health 7, 42. 10.3389/fpubh.2019.00042 30899755 PMC6416195

[B32] Biniossek M. L. Lechel A. Rudolph K. L. Martens U. M. Zimmermann S. (2013). Quantitative proteomic profiling of tumor cell response to telomere dysfunction using isotope-coded protein labeling (ICPL) reveals interaction network of candidate senescence markers. J. Proteomics 91, 515–535. 10.1016/j.jprot.2013.08.007 23969227

[B33] Birch C. A. Molinar-Inglis O. Trejo J. (2021). Subcellular hot spots of GPCR signaling promote vascular inflammation. Curr. Opin. Endocr. Metab. Res. 16, 37–42. 10.1016/j.coemr.2020.07.011 32838054 PMC7431397

[B34] Blagosklonny M. V. (2022). Cell senescence, rapamycin and hyperfunction theory of aging. Cell Cycle 21 (14), 1456–1467. 10.1080/15384101.2022.2054636 35358003 PMC9278457

[B35] Bockaert J. Roussignol G. Bécamel C. Gavarini S. Joubert L. Dumuis A. (2004). GGPCR-interacting proteins (GIPs): nature and functions Biochem. Soc. Trans. 32 (pt 5), 851–855. 10.1042/BST0320851 15494032

[B36] Bohn L. M. McDonald P. H. (2010). Seeking Ligand Bias: Assessing GPCR Coupling to Beta-Arrestins for Drug Discovery. Drug Discov. Today Technol. 7 (1), e37–e42. 10.1016/j.ddtec.2010.06.005 21218149 PMC3014586

[B37] Brobey R. K. Dheghani M. Foster P. P. Rosenblatt K. P. (2015). Klotho Regulates 14-3-3ζ Monomerization and Binding to the ASK1 Signaling Complex in Response to Oxidative Stress. PLoS One 10 (10), e0141968. 10.1371/journal.pone.0141968 26517365 PMC4627807

[B38] Brown D. I. Griendling K. K. (2015). Regulation of signal transduction by reactive oxygen species in the cardiovascular system. Circ. Res. 116 (3), 531–549. 10.1161/CIRCRESAHA.116.303584 25634975 PMC4392388

[B39] Buffenstein R. Edrey Y. H. Yang T. Mele J. (2008). The oxidative stress theory of aging: embattled or invincible? Insights from non-traditional model organisms. Age (Dordr) 30 (2-3), 99–109. 10.1007/s11357-008-9058-z 19424860 PMC2527631

[B40] Burke N. N. Finn D. P. McGuire B. E. Roche M. (2017). Psychological stress in early life as a predisposing factor for the development of chronic pain: Clinical and preclinical evidence and neurobiological mechanisms. J. Neurosci. Res. 95 (6), 1257–1270. 10.1002/jnr.23802 27402412

[B41] Burman A. Kaji I. (2021). Luminal Chemosensory Cells in the Small Intestine. Nutrients 13 (11), 3712. 10.3390/nu13113712 34835968 PMC8620795

[B42] Burns R. N. Moniri N. H. (2011). Agonist- and hydrogen peroxide-mediated oxidation of the β2 adrenergic receptor: evidence of receptor s-sulfenation as detected by a modified biotin-switch assay. J. Pharmacol. Exp. Ther. 339 (3), 914–921. 10.1124/jpet.111.185975 21917560

[B43] Burtscher J. Mallet R. T. Pialoux V. Millet G. P. Burtscher M. (2022). Adaptive Responses to Hypoxia and/or Hyperoxia in Humans. Antioxid. Redox Signal 37, 887–912. 10.1089/ars.2021.0280 35102747

[B44] Buysse D. J. (2014). Sleep health: can we define it? Does it matter? Sleep 37 (1), 9–17. 10.5665/sleep.3298 24470692 PMC3902880

[B45] Caengprasath N. Hanyaloglu A. C. (2019). Hardwiring wire-less networks: spatially encoded GPCR signaling in endocrine systems. Curr. Opin. Cell Biol. 57, 77–82. 10.1016/j.ceb.2018.12.009 30682696

[B46] Cai H. Cong W. N. Daimon C. M. Wang R. Tschöp M. H. Sévigny J. (2013). Altered lipid and salt taste responsivity in ghrelin and GOAT null mice. PLoS One 8 (10), e76553. 10.1371/journal.pone.0076553 24124572 PMC3790684

[B47] Cai H. Maudsley S. Martin B. (2014a). What is the role of metabolic hormones in taste buds of the tongue. Front. Horm. Res. 42, 134–146. 10.1159/000358322 24732931

[B48] Cai H. Daimon C. M. Cong W. N. Wang R. Chirdon P. de Cabo R. (2014b). Longitudinal analysis of calorie restriction on rat taste bud morphology and expression of sweet taste modulators. J. Gerontol. A Biol. Sci. Med. Sci. 69 (5), 532–544. 10.1093/gerona/glt129 24077597 PMC3991138

[B49] Calderwood S. K. Murshid A. Prince T. (2009). The shock of aging: molecular chaperones and the heat shock response in longevity and aging--a mini-review. Gerontology 55 (5), 550–588. 10.1159/000225957 19546513 PMC2754743

[B50] Caroli J. Andreassen S. N. Lorente J. S. Xiao B. Pandy-Szekeres G. Gloriam D. E. (2025). An online GPCR drug discovery resource. Npj Drug Discov. 2, 17. 10.1038/s44386-025-00010-9 PMC13267056 42380595

[B51] Caso V. M. Manzo V. Pecchillo Cimmino T. Conti V. Caso P. Esposito G. (2021). Regulation of Inflammation and Oxidative Stress by Formyl Peptide Receptors in Cardiovascular Disease Progression. Life (Basel) 11 (3), 243. 10.3390/life11030243 33804219 PMC7998928

[B52] Castañeda V. Haro-Vinueza A. Salinas I. Caicedo A. Méndez M. Á. (2022). The MitoAging Project: Single nucleotide polymorphisms (SNPs) in mitochondrial genes and their association to longevity. Mitochondrion 66, 13–26. 10.1016/j.mito.2022.06.008 35817296

[B53] Cattan V. Mercier N. Gardner J. P. Regnault V. Labat C. Mäki-Jouppila J. (2008). Chronic oxidative stress induces a tissue-specific reduction in telomere length in CAST/Ei mice. Free Radic. Biol. Med. 44 (8), 1592–1598. 10.1016/j.freeradbiomed.2008.01.007 18249196

[B54] Caux F. Dubosclard E. Lascols O. Buendia B. Chazouillères O. Cohen A. (2003). A new clinical condition linked to a novel mutation in lamins A and C with generalized lipoatrophy, insulin-resistant diabetes, disseminated leukomelanodermic papules, liver steatosis, and cardiomyopathy. J. Clin. Endocrinol. Metab. 88 (3), 1006–1113. 10.1210/jc.2002-021506 12629077

[B55] Chadwick W. Maudsley S. (2010). “The devil is in the dose: complexity of receptor systems and responses,” in Hormesis (Springer), 95–108.

[B56] Chadwick W. Zhou Y. Park S. S. Wang L. Mitchell N. Stone M. D. (2010a). Minimal peroxide exposure of neuronal cells induces multifaceted adaptive responses. PLoS One 5 (12), e14352. 10.1371/journal.pone.0014352 21179406 PMC3003681

[B57] Chadwick W. Brenneman R. Martin B. Maudsley S. (2010b). Complex and multidimensional lipid raft alterations in a murine model of Alzheimer's disease. Int. J. Alzheimers Dis. 2010, 604792. 10.4061/2010/604792 21151659 PMC2997345

[B58] Chadwick W. Keselman A. Park S. S. Zhou Y. Wang L. Brenneman R. (2011a). Repetitive peroxide exposure reveals pleiotropic mitogen-activated protein kinase signaling mechanisms. J. Signal Transduct. 2011, 636951. 10.1155/2011/636951 21258655 PMC3023409

[B59] Chadwick W. Boyle J. P. Zhou Y. Wang L. Park S. S. Martin B. (2011b). Multiple oxygen tension environments reveal diverse patterns of transcriptional regulation in primary astrocytes. PLoS One 6 (6), e21638. 10.1371/journal.pone.0021638 21738745 PMC3124552

[B60] Chadwick W. Mitchell N. Martin B. Maudsley S. (2012a). Therapeutic targeting of the endoplasmic reticulum in Alzheimer's disease. Curr. Alzheimer Res. 9 (1), 110–119. 10.2174/156720512799015055 22329655 PMC4682200

[B61] Chadwick W. Martin B. Chapter M. C. Park S. S. Wang L. Daimon C. M. (2012b). GIT2 acts as a potential keystone protein in functional hypothalamic networks associated with age-related phenotypic changes in rats. PLoS One 7 (5), e36975. 10.1371/journal.pone.0036975 22606319 PMC3351446

[B62] Chakraborty R. Sikarwar A. S. Hinton M. Dakshinamurti S. Chelikani P. (2017). Characterization of GPCR signaling in hypoxia. Methods Cell Biol. 142, 101–110. 10.1016/bs.mcb.2017.07.005 28964329

[B63] Chapter M. C. White C. M. DeRidder A. Chadwick W. Martin B. Maudsley S. (2010). Chemical modification of class II G protein-coupled receptor ligands: frontiers in the development of peptide analogs as neuroendocrine pharmacological therapies. Pharmacol. Ther. 125 (1), 39–54. 10.1016/j.pharmthera.2009.07.006 19686775 PMC2815023

[B64] Chatzidoukaki O. Goulielmaki E. Schumacher B. Garinis G. A. (2020). DNA Damage Response and Metabolic Reprogramming in Health and Disease. Trends Genet. 36 (10), 777–791. 10.1016/j.tig.2020.06.018 32684438

[B65] Chei S. Song J. H. Oh H. J. Lee K. Jin H. Choi S. H. (2020). Gintonin-Enriched Fraction Suppresses Heat Stress-Induced Inflammation Through LPA Receptor. Molecules 25 (5), 1019. 10.3390/molecules25051019 32106493 PMC7179209

[B66] Chen Y. Fang S. Ding Q. Jiang R. He J. Wang Q. (2021a). ADGRG1 enriches for functional human hematopoietic stem cells following *ex vivo* expansion-induced mitochondrial oxidative stress. J. Clin. Invest 131 (20), e148329. 10.1172/JCI148329 34464351 PMC8516455

[B67] Chen J. Wang D. Zong Y. Yang X. (2021b). DHA Protects Hepatocytes from Oxidative Injury through GPR120/ERK-Mediated Mitophagy. Int. J. Mol. Sci. 22 (11), 5675. 10.3390/ijms22115675 34073582 PMC8198367

[B68] Chen S. Gan D. Lin S. Zhong Y. Chen M. Zou X. (2022). Metformin in aging and aging-related diseases: clinical applications and relevant mechanisms. Theranostics 12 (6), 2722–2740. 10.7150/thno.71360 35401820 PMC8965502

[B69] Ching T. Himmelstein D. S. Beaulieu-Jones B. K. Kalinin A. A. Do B. T. Way G. P. (2018). Opportunities and obstacles for deep learning in biology and medicine. J. R. Soc. Interface 15 (141), 20170387. 10.1098/rsif.2017.0387 29618526 PMC5938574

[B70] Chiu Y. H. Medina C. B. Doyle C. A. Zhou M. Narahari A. K. Sandilos J. K. (2021). Deacetylation as a receptor-regulated direct activation switch for pannexin channels. Nat. Commun. 12 (1), 4482. 10.1038/s41467-021-24825-y 34301959 PMC8302610

[B71] Choi K. M. Haak A. J. Diaz Espinosa A. M. Cummins K. A. Link P. A. Aravamudhan A. (2021). GPCR-mediated YAP/TAZ inactivation in fibroblasts via EPAC1/2, RAP2C, and MAP4K7. J. Cell Physiol. 236 (11), 7759–7774. 10.1002/jcp.30459 34046891 PMC8530868

[B72] Christodoulou C. Banfield G. Cleanthous A. (2010). Self-control with spiking and non-spiking neural networks playing games. J. Physiol. Paris 104 (3-4), 108–117. 10.1016/j.jphysparis.2009.11.013 19944157

[B73] Ciccia A. Elledge S. J. (2010). The DNA damage response: making it safe to play with knives. Mol. Cell 40 (2), 179–204. 10.1016/j.molcel.2010.09.019 20965415 PMC2988877

[B74] Claing A. Perry S. J. Achiriloaie M. Walker J. K. Albanesi J. P. Lefkowitz R. J. (2000). Multiple endocytic pathways of G protein-coupled receptors delineated by GIT1 sensitivity. Proc. Natl. Acad. Sci. U. S. A. 97 (3), 1119–1124. 10.1073/pnas.97.3.1119 10655494 PMC15541

[B75] Cobelens P. M. Kavelaars A. Heijnen C. J. Ribas C. Mayor F. Jr Penela P. (2006). Hydrogen peroxide impairs GRK2 translation via a calpain-dependent and cdk1-mediated pathway. Cell Signal 19 (2), 269–277. 10.1016/j.cellsig.2006.06.009 16963227

[B76] Coffa S. Breitman M. Spiller B. W. Gurevich V. V. (2011). A single mutation in arrestin-2 prevents ERK1/2 activation by reducing c-Raf1 binding. Biochemistry 50 (32), 6951–6958. 10.1021/bi200745k 21732673 PMC3153575

[B77] Cong W. N. Wang R. Cai H. Daimon C. M. Scheibye-Knudsen M. Bohr V. A. (2013). Long-term artificial sweetener acesulfame potassium treatment alters neurometabolic functions in C57BL/6J mice. PLoS One 8 (8), e70257. 10.1371/journal.pone.0070257 23950916 PMC3737213

[B78] Corremans R. Neven E. Maudsley S. Leysen H. De Broe M. E. D'Haese P. C. (2022). Progression of established non-diabetic chronic kidney disease is halted by metformin treatment in rats. Kidney Int. 101 (5), 929–944. 10.1016/j.kint.2022.01.037 35271933

[B79] Costa-Mattioli M. Walter P. (2020). The integrated stress response: From mechanism to disease. Science 368 (6489), eaat5314. 10.1126/science.aat5314 32327570 PMC8997189

[B80] David S. S. O'Shea V. L. Kundu S. (2007). Base-excision repair of oxidative DNA damage. Nature 447 (7147), 941–950. 10.1038/nature05978 17581577 PMC2896554

[B81] Davies N. (2004). “The Dynamic Reti,” in Everyman Chess. 1st edition.

[B82] Davis A. J. Chen D. J. (2013). DNA double strand break repair via non-homologous end-joining. Transl. Cancer Res. 2 (3), 130–143. 10.3978/j.issn.2218-676X.2013.04.02 24000320 PMC3758668

[B83] de Lange T. (2005). Shelterin: the protein complex that shapes and safeguards human telomeres. Genes Dev. 19 (18), 2100–2110. 10.1101/gad.1346005 16166375

[B84] De Ridder W. Azmi A. Clemen C. S. Eichinger L. Hofmann A. Schröder R. (2020). Multisystem proteinopathy due to a homozygous p.Arg159His VCP mutation: A tale of the unexpected. Neurology 94 (8), e785–e796. 10.1212/WNL.0000000000008763 31848255

[B85] Dekker J. M. Schouten E. G. Klootwijk P. Pool J. Swenne C. A. Kromhout D. (1997). Heart rate variability from short electrocardiographic recordings predicts mortality from all causes in middle-aged and elderly men. The Zutphen Study. Am. J. Epidemiol. 145 (10), 899–908. 10.1093/oxfordjournals.aje.a009049 9149661

[B86] Del Castillo J. Katz B. (1957). Interaction at end-plate receptors between different choline derivatives. Proc. R. Soc. Lond B Biol. Sci. 146 (924), 369–381. 10.1098/rspb.1957.0018 13431862

[B87] Del Valle L. G. (2011). Oxidative stress in aging: Theoretical outcomes and clinical evidences in humans. Biomed. Aging Pathol. 1, 1–7. 10.1016/j.biomag.2011.03.001 20950991

[B88] Di Giosia P. Stamerra C. A. Giorgini P. Jamialahamdi T. Butler A. E. Sahebkar A. (2022). The role of nutrition in inflammaging. Ageing Res. Rev. 77, 101596. 10.1016/j.arr.2022.101596 35219904

[B89] Di Micco R. Krizhanovsky V. Baker D. d'Adda di Fagagna F. (2020). Cellular senescence in ageing: from mechanisms to therapeutic opportunities. Nat. Rev. Mol. Cell Biol. 22 (2), 75–95. 10.1038/s41580-020-00314-w 33328614 PMC8344376

[B90] Doan P. Nguyen P. Murugesan A. Subramanian K. Konda M. S. Kalimuthu V. (2021). Targeting Orphan G Protein-Coupled Receptor 17 with T0 Ligand Impairs Glioblastoma Growth. Cancers (Basel) 13 (15), 3773. 10.3390/cancers13153773 34359676 PMC8345100

[B91] Dominic A. Le N. T. Takahashi M. (2022). Loop Between NLRP3 Inflammasome and Reactive Oxygen Species. Antioxid. Redox Signal 36 (10-12), 784–796. 10.1089/ars.2020.8257 34538111

[B92] Donnelly D. Maudsley S. Gent J. P. Moser R. N. Hurrell C. R. Findlay J. B. (1999). Conserved polar residues in the transmembrane domain of the human tachykinin NK2 receptor: functional roles and structural implications. Biochem. J. 339 (1), 55–61. 10.1042/bj3390055 10085227 PMC1220127

[B93] Efeyan A. Comb W. C. Sabatini D. M. (2015). Nutrient-sensing mechanisms and pathways. Nature 517 (7534), 302–310. 10.1038/nature14190 25592535 PMC4313349

[B94] Ekechukwu O. N. Christian M. (2022). Metabolic responses of light and taste receptors - unexpected actions of GPCRs in adipocytes. Rev. Endocr. Metab. Disord. 23 (1), 111–120. 10.1007/s11154-021-09667-9 34195966 PMC8873064

[B95] Engel A. K. Fries P. Singer W. (2001). Dynamic predictions: oscillations and synchrony in top-down processing. Nat. Rev. Neurosci. 2 (10), 704–771. 10.1038/35094565 11584308

[B96] Eo H. S. Choi J. P. Noh S. J. Hur C. G. Kim W. (2007). A combined approach for the classification of G protein-coupled receptors and its application to detect GPCR splice variants. Comput. Biol. Chem. 31 (4), 246–256. 10.1016/j.compbiolchem.2007.05.002 17631418

[B97] Fagerberg L. Hallström B. M. Oksvold P. Kampf C. Djureinovic D. Odeberg J. (2014). Analysis of the human tissue-specific expression by genome-wide integration of transcriptomics and antibody-based proteomics. Mol. Cell Proteomics 13 (2), 397–406. 10.1074/mcp.M113.035600 24309898 PMC3916642

[B98] Faner R. Cruz T. López-Giraldo A. Agustí A. (2014). Network medicine, multimorbidity and the lung in the elderly. Eur. Respir. J. 44 (3), 775–788. 10.1183/09031936.00078714 25063242

[B99] Farahmand S. Goliaei S. Ansari-Pour N. Razaghi-Moghadam Z. (2016). GTA: a game theoretic approach to identifying cancer subnetwork markers. Mol. Biosyst. 12 (3), 818–825. 10.1039/c5mb00684h 26750920

[B100] Ferguson S. S. (2001). Evolving concepts in G protein-coupled receptor endocytosis: the role in receptor desensitization and signaling. Pharmacol. Rev. 53 (1), 1–24. 10.1016/S0031-6997(24)01478-9 11171937

[B101] Ferguson S. S. Downey W. E. Colapietro A. M. Barak L. S. Ménard L. Caron M. G. (1996). Role of beta-arrestin in mediating agonist-promoted G protein-coupled receptor internalization. Science 271 (5247), 363–366. 10.1126/science.271.5247.363 8553074

[B102] Fielder E. von Zglinicki T. Jurk D. (2017). The DNA Damage Response in Neurons: Die by Apoptosis or Survive in a Senescence-Like State? J. Alzheimers Dis. 60 (s1), S107–S131. 10.3233/JAD-161221 28436392

[B103] Foster S. R. Porrello E. R. Purdue B. Chan H. W. Voigt A. Frenzel S. (2013). Expression, regulation and putative nutrient-sensing function of taste GPCRs in the heart. PLoS One 8 (5), e64579. 10.1371/journal.pone.0064579 23696900 PMC3655793

[B104] Franco R. Aguinaga D. Jiménez J. Lillo J. Martínez-Pinilla E. Navarro G. (2018). Biased receptor functionality versus biased agonism in G-protein-coupled receptors. Biomol. Concepts 9 (1), 143–154. 10.1515/bmc-2018-0013 30864350

[B105] Franco R. Villa M. Morales P. Reyes-Resina I. Gutiérrez-Rodríguez A. Jiménez J. (2019). Increased expression of cannabinoid CB2 and serotonin 5-HT1A heteroreceptor complexes in a model of newborn hypoxic-ischemic brain damage. Neuropharmacology 152, 58–66. 10.1016/j.neuropharm.2019.02.004 30738036

[B106] Fransquet P. D. Lacaze P. Saffery R. Phung J. Parker E. Shah R. (2020). Blood DNA methylation signatures to detect dementia prior to overt clinical symptoms. Alzheimers Dement. (Amst) 12 (1), e12056. 10.1002/dad2.12056 32671182 PMC7346866

[B107] Fu Q. Xiang Y. K. (2015). Trafficking of β-Adrenergic Receptors: Implications in Intracellular Receptor Signaling. Prog. Mol. Biol. Transl. Sci. 132, 151–188. 10.1016/bs.pmbts.2015.03.008 26055058 PMC7328190

[B108] Fu D. Li P. Sheng Q. Lv Z. (2019). β-arrestin-2 enhances intestinal epithelial apoptosis in necrotizing enterocolitis. Aging (Albany NY) 11 (19), 8294–8312. 10.18632/aging.102320 31612867 PMC6814604

[B109] Fu R. Jiang X. Yang Y. Wang C. Zhang Y. Zhu Y. (2022). Bidirectional regulation of structural damage on autophagy in the *C. elegans* epidermis. Autophagy 18 (11), 2731–2745. 10.1080/15548627.2022.2047345 35311461 PMC9629849

[B110] Fuentes N. McCullough M. Panettieri R. A. Jr Druey K. M. (2021). RGS proteins, GRKs, and beta-arrestins modulate G protein-mediated signaling pathways in asthma. Pharmacol. Ther. 223, 107818. 10.1016/j.pharmthera.2021.107818 33600853 PMC8192426

[B111] Fukami M. Suzuki E. Igarashi M. Miyado M. Ogata T. (2018). Gain-of-function mutations in G-protein-coupled receptor genes associated with human endocrine disorders. Clin. Endocrinol. (Oxf) 88 (3), 351–359. 10.1111/cen.13496 29029377

[B112] Gallardo P. Salas-Pino S. Daga R. R. (2021). Reversible protein aggregation as cytoprotective mechanism against heat stress. Curr. Genet. 67 (6), 849–855. 10.1007/s00294-021-01191-2 34091720 PMC8592950

[B113] Gandhirajan R. K. Jain M. Walla B. Johnsen M. Bartram M. P. Huynh Anh M. (2016). Cysteine S-Glutathionylation Promotes Stability and Activation of the Hippo Downstream Effector Transcriptional Co-activator with PDZ-binding Motif (TAZ). J. Biol. Chem. 291 (22), 11596–11607. 10.1074/jbc.M115.712539 27048650 PMC4882430

[B114] Gao X. Yu X. Zhang C. Wang Y. Sun Y. Sun H. (2022). Telomeres and Mitochondrial Metabolism: Implications for Cellular Senescence and Age-related Diseases. Stem Cell Rev. Rep. 18 (7), 2315–2327. 10.1007/s12015-022-10370-8 35460064 PMC9033418

[B115] Gao C. Jiang J. Tan Y. Chen S. (2023). Microglia in neurodegenerative diseases: mechanism and potential therapeutic targets. Signal Transduct. Target Ther. 8 (1), 359. 10.1038/s41392-023-01588-0 37735487 PMC10514343

[B116] Ge L. Liu J. Zhang Y. Dehmer M. (2019). Identifying anticancer peptides by using a generalized chaos game representation. J. Math. Biol. 78 (1-2), 441–463. 10.1007/s00285-018-1279-x 30291366

[B117] Geerts H. Dacks P. A. Devanarayan V. Haas M. Khachaturian Z. S. Gordon M. F. (2016). Big data to smart data in Alzheimer's disease: The brain health modeling initiative to foster actionable knowledge. Alzheimers Dement. 12 (9), 1014–1021. 10.1016/j.jalz.2016.04.008 27238630

[B118] Germano J. F. Huang C. Sin J. Song Y. Tucker K. C. Taylor D. J. R. (2020). Intermittent Use of a Short-Course Glucagon-like Peptide-1 Receptor Agonist Therapy Limits Adverse Cardiac Remodeling via Parkin-dependent Mitochondrial Turnover. Sci. Rep. 10 (1), 8284. 10.1038/s41598-020-64924-2 32427925 PMC7237417

[B119] Gery S. Komatsu N. Baldjyan L. Yu A. Koo D. Koeffler H. P. (2006). The circadian gene per1 plays an important role in cell growth and DNA damage control in human cancer cells. Mol. Cell 22 (3), 375–382. 10.1016/j.molcel.2006.03.038 16678109

[B120] Gesty-Palmer D. Chen M. Reiter E. Ahn S. Nelson C. D. Wang S. (2006). Distinct beta-arrestin- and G protein-dependent pathways for parathyroid hormone receptor-stimulated ERK1/2 activation. J. Biol. Chem. 281 (16), 10856–10864. 10.1074/jbc.M513380200 16492667

[B121] Gesty-Palmer D. Yuan L. Martin B. Wood W. H. Lee M. H. Janech M. G. (2013). β-arrestin-selective G protein-coupled receptor agonists engender unique biological efficacy *in vivo* . Mol. Endocrinol. 27 (2), 296–314. 10.1210/me.2012-1091 23315939 PMC3683806

[B122] Glaser R. Kiecolt-Glaser J. K. (2005). Stress-induced immune dysfunction: implications for health. Nat. Rev. Immunol. 5 (3), 243–251. 10.1038/nri1571 15738954

[B123] Golubev A. Hanson A. D. Gladyshev V. N. (2017). Non-enzymatic molecular damage as a prototypic driver of aging. J. Biol. Chem. 292 (15), 6029–6038. 10.1074/jbc.R116.751164 28264930 PMC5391736

[B124] Gong H. Sun H. Koch W. J. Rau T. Eschenhagen T. Ravens U. (2002). Specific beta(2)AR blocker ICI 118,551 actively decreases contraction through a G(i)-coupled form of the beta(2)AR in myocytes from failing human heart. Circulation 105 (21), 2497–2503. 10.1161/01.cir.0000017187.61348.95 12034656

[B125] Goyal M. S. Blazey T. M. Su Y. Couture L. E. Durbin T. J. Bateman R. J. (2019). Persistent metabolic youth in the aging female brain. Proc. Natl. Acad. Sci. U. S. A. 116 (8), 3251–3255. 10.1073/pnas.1815917116 30718410 PMC6386682

[B126] Graham M. K. Meeker A. (2017). Telomeres and telomerase in prostate cancer development and therapy. Nat. Rev. Urol. 14 (10), 607–619. 10.1038/nrurol.2017.104 28675175 PMC5626660

[B127] Green C. L. Lamming D. W. Fontana L. (2022). Molecular mechanisms of dietary restriction promoting health and longevity. Nat. Rev. Mol. Cell Biol. 23 (1), 56–73. 10.1038/s41580-021-00411-4 34518687 PMC8692439

[B128] Grieder M. Wang D. J. J. Dierks T. Wahlund L. O. Jann K. (2018). Default Mode Network Complexity and Cognitive Decline in Mild Alzheimer's Disease. Front. Neurosci. 12, 770. 10.3389/fnins.2018.00770 30405347 PMC6206840

[B129] Grundmann M. Merten N. Malfacini D. Inoue A. Preis P. Simon K. (2018). Lack of beta-arrestin signaling in the absence of active G proteins. Nat. Commun. 9 (1), 341. 10.1038/s41467-017-02661-3 29362459 PMC5780443

[B130] Guo J. Chiang W. C. (2021). Mitophagy in aging and longevity. IUBMB Life 74 (4), 296–316. 10.1002/iub.2585 34889504

[B131] Guo M. Cai C. Zhao G. Qiu X. Zhao H. Ma Q. (2014). Hypoxia promotes migration and induces CXCR4 expression via HIF-1α activation in human osteosarcoma. PLoS One 9 (3), e90518. 10.1371/journal.pone.0090518 24618817 PMC3949690

[B132] Gupta A. Storey K. B. (2021). Activation of the Hippo Pathway in *Rana sylvatica*: Yapping Stops in Response to Anoxia. Life (Basel) 11 (12), 1422. 10.3390/life11121422 34947952 PMC8708225

[B133] Gurevich V. V. Gurevich E. V. (2018). Arrestins and G proteins in cellular signaling: The coin has two sides. Sci. Signal 11 (549), eaav1646. 10.1126/scisignal.aav1646 30254054 PMC6390952

[B134] Hall H. Perelman D. Breschi A. Limcaoco P. Kellogg R. McLaughlin T. (2018). Glucotypes reveal new patterns of glucose dysregulation. PLoS Biol. 16 (7), e2005143. 10.1371/journal.pbio.2005143 30040822 PMC6057684

[B135] Hanyaloglu A. C. von Zastrow M. (2008). Regulation of GPCRs by endocytic membrane trafficking and its potential implications. Annu. Rev. Pharmacol. Toxicol. 48, 537–668. 10.1146/annurev.pharmtox.48.113006.094830 18184106

[B136] Haque M. E. Akther M. Azam S. Choi D. K. Kim I. S. (2020). GPR4 Knockout Improves the Neurotoxin-Induced, Caspase-Dependent Mitochondrial Apoptosis of the Dopaminergic Neuronal Cell. Int. J. Mol. Sci. 21 (20), 7517. 10.3390/ijms21207517 33053856 PMC7589616

[B137] Hara M. R. Kovacs J. J. Whalen E. J. Rajagopal S. Strachan R. T. Grant W. (2011). A stress response pathway regulates DNA damage through β2-adrenoreceptors and β-arrestin-1. Nature 477 (7364), 349–353. 10.1038/nature10368 21857681 PMC3628753

[B138] Harries L. W. (2022). Dysregulated RNA processing and metabolism: a new hallmark of ageing and provocation for cellular senescence. FEBS J. 290, 1221–1234. 10.1111/febs.16462 35460337

[B139] Hayashi A. Takemoto M. Shoji M. Hattori A. Sugita K. Yokote K. (2015). Pioglitazone improves fat tissue distribution and hyperglycemia in a case of cockayne syndrome with diabetes. Diabetes Care 38 (5), e76. 10.2337/dc14-2944 25908161

[B140] Hayflick L. Moorhead P. S. (1961). The serial cultivation of human diploid cell strains. Exp. Cell Res. 25, 585–621. 10.1016/0014-4827(61)90192-6 13905658

[B141] Hegde M. L. Hazra T. K. Mitra S. (2008). Early steps in the DNA base excision/single-strand interruption repair pathway in mammalian cells. Cell Res. 18 (1), 27–47. 10.1038/cr.2008.8 18166975 PMC2692221

[B142] Heilman C. Ibarreta J. Salonga G. A. Hon M. C. Caracci A. M. Badial T. (2020). G protein coupled receptor kinases modulate *Caenorhabditis elegans* reactions to heat stresses. Biochem. Biophys. Res. Commun. 530 (4), 692–698. 10.1016/j.bbrc.2020.07.121 32768194

[B143] Hendrickx J. O. van Gastel J. Leysen H. Martin B. Maudsley S. (2020). High-dimensionality Data Analysis of Pharmacological Systems Associated with Complex Diseases. Pharmacol. Rev. 72, 191–217. 10.1124/pr.119.017921 31843941

[B144] Hinden L. Kogot-Levin A. Tam J. Leibowitz G. (2022). Pathogenesis of diabesity-induced kidney disease: role of kidney nutrient sensing. FEBS J. 289 (4), 901–921. 10.1111/febs.15790 33630415

[B145] Hipp M. S. Kasturi P. Hartl F. U. (2019). The proteostasis network and its decline in ageing. Nat. Rev. Mol. Cell Biol. 20 (7), 421–435. 10.1038/s41580-019-0101-y 30733602

[B146] Hoefen R. J. Berk B. C. (2006). The multifunctional GIT family of proteins. J. Cell Sci. 119 (Pt 8), 1469–1475. 10.1242/jcs.02925 16598076

[B147] Hollien J. Weissman J. S. (2006). Decay of endoplasmic reticulum-localized mRNAs during the unfolded protein response. Science 313 (5783), 104–117. 10.1126/science.1129631 16825573

[B148] Hong S. T. Mah W. (2015). A Critical Role of GIT1 in Vertebrate and Invertebrate Brain Development. Exp. Neurobiol. 24 (1), 8–16. 10.5607/en.2015.24.1.8 25792865 PMC4363336

[B149] Hou N. Wen Y. Yuan X. Xu H. Wang X. Li F. (2017). Activation of Yap1/Taz signaling in ischemic heart disease and dilated cardiomyopathy. Exp. Mol. Pathol. 103 (3), 267–275. 10.1016/j.yexmp.2017.11.006 29154888 PMC5988229

[B150] Hsieh C. C. Kuro-o M. Rosenblatt K. P. Brobey R. Papaconstantinou J. (2010). The ASK1-Signalosome regulates p38 MAPK activity in response to levels of endogenous oxidative stress in the Klotho mouse models of aging. Aging (Albany NY) 2 (9), 597–611. 10.18632/aging.100194 20844314 PMC2984608

[B151] Hsu Y. C. Ip M. M. (2011). Conjugated linoleic acid-induced apoptosis in mouse mammary tumor cells is mediated by both G protein coupled receptor-dependent activation of the AMP-activated protein kinase pathway and by oxidative stress. Cell Signal 23 (12), 2013–2220. 10.1016/j.cellsig.2011.07.015 21821121 PMC3265966

[B152] Hu H. T. Ma Q. Y. Zhang D. Shen S. G. Han L. Ma Y. D. (2009). HIF-1alpha links beta-adrenoceptor agonists and pancreatic cancer cells under normoxic condition. Acta Pharmacol. Sin. 31 (1), 102–110. 10.1038/aps.2009.181 20037603 PMC4002695

[B153] Hu J. X. Thomas C. E. Brunak S. (2016). Network biology concepts in complex disease comorbidities. Nat. Rev. Genet. 17 (10), 615–629. 10.1038/nrg.2016.87 27498692

[B154] Huang J. Stewart A. Maity B. Hagen J. Fagan R. L. Yang J. (2014). RGS6 suppresses Ras-induced cellular transformation by facilitating Tip60-mediated Dnmt1 degradation and promoting apoptosis. Oncogene 33 (27), 3604–3611. 10.1038/onc.2013.324 23995786 PMC4232305

[B155] Huang Y. F. Gu C. J. Wang Q. Xu L. Chen J. Zhou W. (2020). The protective effort of GPCR kinase 2-interacting protein-1 in neurons via promoting Beclin1-Parkin induced mitophagy at the early stage of spinal cord ischemia-reperfusion injury. FASEB J. 34 (2), 2055–2074. 10.1096/fj.201902047R 31908016

[B156] Huang L. Liu Q. Zhou T. Zhang J. Tian Q. Zhang Q. (2021). Deficiency of β-arrestin2 alleviates apoptosis through GRP78-ATF6-CHOP signaling pathway in primary Sjögren's syndrome. Int. Immunopharmacol. 101 (Pt A), 108281. 10.1016/j.intimp.2021.108281 34710848

[B157] Hwang J. Kim H. S. Kang B. S. Kim D. H. Ryoo Z. Y. Choi S. U. (2015). RGS19 converts iron deprivation stress into a growth-inhibitory signal. Biochem. Biophys. Res. Commun. 464 (1), 168–175. 10.1016/j.bbrc.2015.06.109 26116529

[B158] Ibrahim I. M. Abdelmalek D. H. Elfiky A. A. (2019). GRP78: A cell's response to stress. Life Sci. 226, 156–163. 10.1016/j.lfs.2019.04.022 30978349 PMC7094232

[B159] Ilter M. Mansoor S. Sensoy O. (2019). Utilization of Biased G Protein-Coupled Receptor Signaling towards Development of Safer and Personalized Therapeutics. Molecules 24 (11), 2052. 10.3390/molecules24112052 31146474 PMC6600667

[B160] Jaggupilli A. Howard R. Aluko R. E. Chelikani P. (2019). Advanced Glycation End-Products Can Activate or Block Bitter Taste Receptors. Nutrients 11 (6), 1317. 10.3390/nu11061317 31212814 PMC6628017

[B161] Janssens J. Etienne H. Idriss S. Azmi A. Martin B. Maudsley S. (2014). Systems-Level G Protein-Coupled Receptor Therapy Across a Neurodegenerative Continuum by the GLP-1 Receptor System. Front. Endocrinol. (Lausanne) 5, 142. 10.3389/fendo.2014.00142 25225492 PMC4150252

[B162] Jasin M. Rothstein R. (2013). Repair of strand breaks by homologous recombination. Cold Spring Harb. Perspect. Biol. 5, a012740. 10.1101/cshperspect.a012740 24097900 PMC3809576

[B163] Jestin M. Kapnick S. M. Tarasenko T. N. Burke C. T. Zerfas P. M. Diaz F. (2020). Mitochondrial disease disrupts hepatic allostasis and lowers the threshold for immune-mediated liver toxicity. Mol. Metab. 37, 100981. 10.1016/j.molmet.2020.100981 32283081 PMC7167504

[B164] Jia J. J. Zeng X. S. Zhou X. S. Li Y. Bai J. (2014). The induction of thioredoxin-1 by epinephrine withdraws stress via interaction with β-arrestin-1. Cell Cycle 13 (19), 3121–3321. 10.4161/15384101.2014.949214 25486571 PMC4614835

[B165] Jia Y. Gu H. Luo Q. (2017). Sample entropy reveals an age-related reduction in the complexity of dynamic brain. Sci. Rep. 7 (1), 7990. 10.1038/s41598-017-08565-y 28801672 PMC5554148

[B166] Jung S. Son H. Lee D. H. Roh G. S. Kang S. S. Cho G. J. (2016). Decreased levels of RGS4 in the paraventricular nucleus facilitate GABAergic inhibition during the acute stress response. Biochem. Biophys. Res. Commun. 472 (1), 276–280. 10.1016/j.bbrc.2016.02.108 26926565

[B167] Jurk D. Wilson C. Passos J. F. Oakley F. Correia-Melo C. Greaves L. (2014). Chronic inflammation induces telomere dysfunction and accelerates ageing in mice. Nat. Commun. 2, 4172. 10.1038/ncomms5172 24960204 PMC4090717

[B168] Kaczanowski S. Klim J. Zielenkiewicz U. (2018). An Apoptotic and Endosymbiotic Explanation of the Warburg and the Inverse Warburg Hypotheses. Int. J. Mol. Sci. 19 (10), 3100. 10.3390/ijms19103100 30308966 PMC6213112

[B169] Kalisch R. Müller M. B. Tüscher O. (2015). A conceptual framework for the neurobiological study of resilience. Behav. Brain Sci. 38, e92. 10.1017/S0140525X1400082X 25158686

[B170] Kang J. Shi Y. Xiang B. Qu B. Su W. Zhu M. (2005). A nuclear function of beta-arrestin1 in GPCR signaling: regulation of histone acetylation and gene transcription. Cell 123 (5), 833–847. 10.1016/j.cell.2005.09.011 16325578

[B171] Kania E. Pająk B. Orzechowski A. (2015). Calcium homeostasis and ER stress in control of autophagy in cancer cells. Biomed. Res. Int. 2015, 352794. 10.1155/2015/352794 25821797 PMC4363509

[B172] Kashihara T. Sadoshima J. (2019). Role of YAP/TAZ in Energy Metabolism in the Heart. J. Cardiovasc Pharmacol. 74 (6), 483–490. 10.1097/FJC.0000000000000736 31815864 PMC6905197

[B173] Kataoka K. Bilkei-Gorzo A. Nozaki C. Togo A. Nakamura K. Ohta K. (2020). Age-dependent Alteration in Mitochondrial Dynamics and Autophagy in Hippocampal Neuron of Cannabinoid CB1 Receptor-deficient Mice. Brain Res. Bull. 160, 40–49. 10.1016/j.brainresbull.2020.03.014 32294520

[B174] Kaul Z. Cesare A. J. Huschtscha L. I. Neumann A. A. Reddel R. R. (2011). Five dysfunctional telomeres predict onset of senescence in human cells. EMBO Rep. 13 (1), 52–59. 10.1038/embor.2011.227 22157895 PMC3246253

[B175] Kawakami M. Hattori M. Ohashi W. Fujimori T. Hattori K. Takebe M. (2018). Role of G protein-coupled receptor kinase 2 in oxidative and nitrosative stress-related neurohistopathological changes in a mouse model of sepsis-associated encephalopathy. J. Neurochem. 145 (6), 474–488. 10.1111/jnc.14329 29500815

[B176] Kenakin T. (2019). Biased Receptor Signaling in Drug Discovery. Pharmacol. Rev. 71 (2), 267–315. 10.1124/pr.118.016790 30914442

[B177] Khoury E. Nikolajev L. Simaan M. Namkung Y. Laporte S. A. (2014). Differential regulation of endosomal GPCR/β-arrestin complexes and trafficking by MAPK. J. Biol. Chem. 289 (34), 23302–23317. 10.1074/jbc.M114.568147 25016018 PMC4156072

[B178] Kimura M. Mizukami Y. Miura T. Fujimoto K. Kobayashi S. Matsuzaki M. (2001). Orphan G protein-coupled receptor, GPR41, induces apoptosis via a p53/Bax pathway during ischemic hypoxia and reoxygenation. J. Biol. Chem. 276 (28), 26453–26460. 10.1074/jbc.M101289200 11335718

[B179] Kliment C. R. Oury T. D. (2010). Oxidative stress, extracellular matrix targets, and idiopathic pulmonary fibrosis. Free Radic. Biol. Med. 49 (5), 707–717. 10.1016/j.freeradbiomed.2010.04.036 20452419 PMC13063126

[B180] Kohout T. A. Nicholas S. L. Perry S. J. Reinhart G. Junger S. Struthers R. S. (2004). Differential desensitization, receptor phosphorylation, beta-arrestin recruitment, and ERK1/2 activation by the two endogenous ligands for the CC chemokine receptor 7. J. Biol. Chem. 279 (22), 23214–23222. 10.1074/jbc.M402125200 15054093

[B181] Koob G. F. Schulkin J. (2019). Addiction and stress: An allostatic view. Neurosci. Biobehav Rev. 106, 245–262. 10.1016/j.neubiorev.2018.09.008 30227143

[B182] Kourtis N. Tavernarakis N. (2011). Cellular stress response pathways and ageing: intricate molecular relationships. EMBO J. 30 (13), 2520–2531. 10.1038/emboj.2011.162 21587205 PMC3155297

[B183] Kreienkamp H. J. (2002). Organisation of G-protein-coupled receptor signalling complexes by scaffolding proteins. Curr. Opin. Pharmacol. 2 (5), 581–586. 10.1016/s1471-4892(02)00203-5 12324263

[B184] Kumari N. Reabroi S. North B. J. (2021). Unraveling the Molecular Nexus between GPCRs, ERS, and EMT. Mediat. Inflamm. 2021, 6655417. 10.1155/2021/6655417 33746610 PMC7943314

[B185] Kurhaluk N. Tkachenko H. Lukash O. (2021). Photoperiod-induced alterations in biomarkers of oxidative stress and biochemical pathways in rats of different ages: Focus on individual physiological reactivity. Chronobiol Int. 38 (12), 1673–1691. 10.1080/07420528.2021.1939364 34121553

[B186] Kurop M. K. Huyen C. M. Kelly J. H. Blagg B. S. J. (2021). The heat shock response and small molecule regulators. Eur. J. Med. Chem. 226, 113846. 10.1016/j.ejmech.2021.113846 34563965 PMC8608735

[B187] Kwon Y. Mehta S. Clark M. Walters G. Zhong Y. Lee H. N. (2022). Non-canonical β-adrenergic activation of ERK at endosomes. Nature 611 (7934), 173–179. 10.1038/s41586-022-05343-3 36289326 PMC10031817

[B188] Lappano R. Rigiracciolo D. De Marco P. Avino S. Cappello A. R. Rosano C. (2016). Recent Advances on the Role of G Protein-Coupled Receptors in Hypoxia-Mediated Signaling. AAPS J. 18 (2), 305–310. 10.1208/s12248-016-9881-6 26865461 PMC4779097

[B189] Lee S. W. (2019). A Copernican Approach to Brain Advancement: The Paradigm of Allostatic Orchestration. Front. Hum. Neurosci. 13, 129. 10.3389/fnhum.2019.00129 31105539 PMC6499026

[B190] Lee J. K. Bou Dagher J. (2016). Regulator of G-protein Signaling (RGS)1 and RGS10 Proteins as Potential Drug Targets for Neuroinflammatory and Neurodegenerative Diseases. AAPS J. 18 (3), 545–549. 10.1208/s12248-016-9883-4 26902301 PMC5256602

[B191] Lee S. J. No Y. R. Dang D. T. Dang L. H. Yang V. W. Shim H. (2013). Regulation of hypoxia-inducible factor 1α (HIF-1α) by lysophosphatidic acid is dependent on interplay between p53 and Krüppel-like factor 5. J. Biol. Chem. 288 (35), 25244–25253. 10.1074/jbc.M113.489708 23880760 PMC3757187

[B192] Lee K. W. Woo J. M. Im J. Y. Park E. S. He L. Ichijo H. (2014). Apoptosis signal-regulating kinase 1 modulates the phenotype of α-synuclein transgenic mice. Neurobiol. Aging 36 (1), 519–526. 10.1016/j.neurobiolaging.2014.07.034 25219466 PMC4268347

[B193] Lee P. Chandel N. S. Simon M. C. (2020). Cellular adaptation to hypoxia through hypoxia inducible factors and beyond. Nat. Rev. Mol. Cell Biol. 21 (5), 268–283. 10.1038/s41580-020-0227-y 32144406 PMC7222024

[B194] Lee G. J. Kim Y. J. Park B. Yim S. Park C. Roh H. (2022). YAP-dependent Wnt5a induction in hypertrophic adipocytes restrains adiposity. Cell Death Dis. 13 (4), 407. 10.1038/s41419-022-04847-0 35478181 PMC9046197

[B195] Leslie R. D. Vartak T. (2020). Allostasis and the origins of adult-onset diabetes. Diabetologia 63 (2), 261–265. 10.1007/s00125-019-05048-9 31813006 PMC6946720

[B196] Levy A. Stedman A. Deutsch E. Donnadieu F. Virgin H. W. Sansonetti P. J. (2020). Innate immune receptor NOD2 mediates LGR5+ intestinal stem cell protection against ROS cytotoxicity via mitophagy stimulation. Proc. Natl. Acad. Sci. U. S. A. 117 (4), 1994–2003. 10.1073/pnas.1902788117 31919280 PMC6994981

[B197] Leysen H. van Gastel J. Hendrickx J. O. Santos-Otte P. Martin B. Maudsley S. (2018). G Protein-Coupled Receptor Systems as Crucial Regulators of DNA Damage Response Processes. Int. J. Mol. Sci. 19 (10), 2919. 10.3390/ijms19102919 30261591 PMC6213947

[B198] Leysen H. Walter D. Christiaenssen B. Vandoren R. Harputluoğlu İ. Van Loon N. (2021). GPCRs Are Optimal Regulators of Complex Biological Systems and Orchestrate the Interface between Health and Disease. Int. J. Mol. Sci. 22 (24), 13387. 10.3390/ijms222413387 34948182 PMC8708147

[B199] Leysen H. Walter D. Clauwaert L. Hellemans L. van Gastel J. Vasudevan L. (2022). The Relaxin-3 Receptor, RXFP3, Is a Modulator of Aging-Related Disease. Int. J. Mol. Sci. 23 (8), 4387. 10.3390/ijms23084387 35457203 PMC9027355

[B200] Li J. Cao Y. X. Cao L. Liu Y. Xu C. B. (2008). Heat stress alters G-protein coupled receptor-mediated function and endothelium-dependent relaxation in rat mesenteric artery. Eur. J. Pharmacol. 588 (2-3), 280–285. 10.1016/j.ejphar.2008.04.038 18511037

[B201] Li H. Lou R. Xu X. Xu C. Yu Y. Xu Y. (2021). The variations in human orphan G protein-coupled receptor QRFPR affect PI3K-AKT-mTOR signaling. J. Clin. Lab. Anal. 35 (7), e23822. 10.1002/jcla.23822 34018631 PMC8275006

[B202] Li L. Liu H. Yu J. Sun Z. Jiang M. Yu H. (2023). Intestinal Microbiota and Metabolomics Reveal the Role of *Auricularia delicate* in Regulating Colitis-Associated Colorectal Cancer. Nutrients 15 (23), 5011. 10.3390/nu15235011 38068869 PMC10708550

[B203] Liang F. Zhang H. Cheng D. Gao H. Wang J. Yue J. (2021). Ablation of LGR4 signaling enhances radiation sensitivity of prostate cancer cells. Life Sci. 265, 118737. 10.1016/j.lfs.2020.118737 33171177

[B204] Lidzbarsky G. Gutman D. Shekhidem H. A. Sharvit L. Atzmon G. (2018). Genomic Instabilities, Cellular Senescence, and Aging: *In Vitro*, *In Vivo* and Aging-Like Human Syndromes. Front. Med. (Lausanne) 5, 104. 10.3389/fmed.2018.00104 29719834 PMC5913290

[B205] Liguori I. Russo G. Curcio F. Bulli G. Aran L. Della-Morte D. (2018). Oxidative stress, aging, and diseases. Clin. Interv. Aging 13, 757–772. 10.2147/CIA.S158513 29731617 PMC5927356

[B206] Lin L. Cao L. Liu Y. Wang K. Zhang X. Qin X. (2019a). B7-H3 promotes multiple myeloma cell survival and proliferation by ROS-dependent activation of Src/STAT3 and c-Cbl-mediated degradation of SOCS3. Leukemia 33 (6), 1475–1486. 10.1038/s41375-018-0331-6 30573782

[B207] Lin Y. Jiang M. Chen W. Zhao T. Wei Y. (2019b). Cancer and ER stress: Mutual crosstalk between autophagy, oxidative stress and inflammatory response. Biomed. Pharmacother. 118, 109249. 10.1016/j.biopha.2019.109249 31351428

[B208] Lipsitz L. A. Goldberger A. L. (1992). Loss of 'complexity' and aging. Potential applications of fractals and chaos theory to senescence. JAMA 267 (13), 1806–1809. 10.1001/jama.1992.03480130122036 1482430

[B209] Liu Y. Yang Y. Ward R. An S. Guo X. X. Li W. (2015). Biased signalling: the instinctive skill of the cell in the selection of appropriate signalling pathways. Biochem. J. 470 (2), 155–167. 10.1042/BJ20150358 26348905

[B210] Liu Z. Jiang J. He Q. Liu Z. Yang Z. Xu J. (2019). β-Arrestin1-mediated decrease in endoplasmic reticulum stress impairs intestinal stem cell proliferation following radiation. FASEB J. 33 (9), 10165–10176. 10.1096/fj.201900376RRR 31207192

[B211] Liu X. Xiao W. Jiang Y. Zou L. Chen F. Xiao W. (2021). Bmal1 Regulates the Redox Rhythm of HSPB1, and Homooxidized HSPB1 Attenuates the Oxidative Stress Injury of Cardiomyocytes. Oxid. Med. Cell Longev. 2021, 5542815. 10.1155/2021/5542815 34239687 PMC8238613

[B212] Löchel H. F. Eger D. Sperlea T. Heider D. (2020). Deep learning on chaos game representation for proteins. Bioinformatics 36 (1), 272–279. 10.1093/bioinformatics/btz493 31225868

[B213] López-Otín C. Blasco M. A. Partridge L. Serrano M. Kroemer G. (2013). The hallmarks of aging. Cell 153 (6), 1194–1217. 10.1016/j.cell.2013.05.039 23746838 PMC3836174

[B214] López-Otín C. Galluzzi L. Freije J. M. P. Madeo F. Kroemer G. (2016). Metabolic Control of Longevity. Cell 166 (4), 802–821. 10.1016/j.cell.2016.07.031 27518560

[B215] López-Otín C. Blasco M. A. Partridge L. Serrano M. Kroemer G. (2023). Hallmarks of aging: An expanding universe. Cell 186 (2), 243–278. 10.1016/j.cell.2022.11.001 36599349

[B216] Lu R. Pickett H. A. (2022). Telomeric replication stress: the beginning and the end for alternative lengthening of telomeres cancers. Open Biol. 12 (3), 220011. 10.1098/rsob.220011 35259951 PMC8905155

[B217] Lu D. Cai H. Park S. S. Siddiqui S. Premont R. T. Schmalzigaug R. (2015). Nuclear GIT2 is an ATM substrate and promotes DNA repair. Mol. Cell Biol. 35 (7), 1081–1096. 10.1128/MCB.01432-14 25605334 PMC4355538

[B218] Luo J. Mills K. le Cessie S. Noordam R. van Heemst D. (2020a). Ageing, age-related diseases and oxidative stress: What to do next? Ageing Res. Rev. 57, 100982. 10.1016/j.arr.2019.100982 31733333

[B219] Luo M. Meng Z. Moroishi T. Lin K. C. Shen G. Mo F. (2020b). Heat stress activates YAP/TAZ to induce the heat shock transcriptome. Nat. Cell Biol. 22 (12), 1447–1459. 10.1038/s41556-020-00602-9 33199845 PMC7757600

[B220] Luttrell L. M. Lefkowitz R. J. (2002). The role of beta-arrestins in the termination and transduction of G-protein-coupled receptor signals. J. Cell Sci. 115 (3), 455–465. 10.1242/jcs.115.3.455 11861753

[B221] Luttrell L. M. Ferguson S. S. Daaka Y. Miller W. E. Maudsley S. Della Rocca G. J. (1999). Beta-arrestin-dependent formation of beta2 adrenergic receptor-Src protein kinase complexes. Science 283 (5402), 655–661. 10.1126/science.283.5402.655 9924018

[B222] Luttrell L. M. Maudsley S. Bohn L. M. (2015). Fulfilling the Promise of “Biased” G Protein-Coupled Receptor Agonism. Mol. Pharmacol. 88 (3), 579–588. 10.1124/mol.115.099630 26134495 PMC4551052

[B223] Madabhushi R. Pan L. Tsai L. H. (2014). DNA damage and its links to neurodegeneration. Neuron 83 (2), 266–282. 10.1016/j.neuron.2014.06.034 25033177 PMC5564444

[B224] Maejima Y. Zablocki D. Nah J. Sadoshima J. (2022). The role of the Hippo pathway in autophagy in the heart. Cardiovasc Res. 12, cvac014–3330. 10.1093/cvr/cvac014 35150237 PMC10060713

[B225] Mantas I. Saarinen M. Xu Z. D. Svenningsson P. (2022). Update on GPCR-based targets for the development of novel antidepressants. Mol. Psychiatry 27 (1), 534–558. 10.1038/s41380-021-01040-1 33589739 PMC8960420

[B226] Mari P. O. Florea B. I. Persengiev S. P. Verkaik N. S. Brüggenwirth H. T. Modesti M. (2006). Dynamic assembly of end-joining complexes requires interaction between Ku70/80 and XRCC4. Proc. Natl. Acad. Sci. U. S. A. 103 (49), 18597–18602. 10.1073/pnas.0609061103 17124166 PMC1693708

[B227] Marti-Solano M. Crilly S. E. Malinverni D. Munk C. Harris M. Pearce A. (2020). Combinatorial expression of GPCR isoforms affects signalling and drug responses. Nature 587 (7835), 650–656. 10.1038/s41586-020-2888-2 33149304 PMC7611127

[B228] Martin B. Pearson M. Kebejian L. Golden E. Keselman A. Bender M. (2007). Sex-dependent metabolic, neuroendocrine, and cognitive responses to dietary energy restriction and excess. Endocrinology 148 (9), 4318–4333. 10.1210/en.2007-0161 17569758 PMC2622430

[B229] Martin B. Pearson M. Brenneman R. Golden E. Keselman A. Iyun T. (2008). Conserved and differential effects of dietary energy intake on the hippocampal transcriptomes of females and males. PLoS One 3 (6), e2398. 10.1371/journal.pone.0002398 18545695 PMC2405949

[B230] Martin B. Brenneman R. Golden E. Walent T. Becker K. G. Prabhu V. V. (2009a). Growth factor signals in neural cells: coherent patterns of interaction control multiple levels of molecular and phenotypic responses. J. Biol. Chem. 284 (4), 2493–2511. 10.1074/jbc.M804545200 19038969 PMC2629109

[B231] Martin B. Pearson M. Brenneman R. Golden E. Wood W. Prabhu V. (2009b). Gonadal transcriptome alterations in response to dietary energy intake: sensing the reproductive environment. PLoS One 4 (1), e4146. 10.1371/journal.pone.0004146 19127293 PMC2607546

[B232] Martin B. Maudsley S. White C. M. Egan J. M. (2009c). Hormones in the naso-oropharynx: endocrine modulation of taste and smell. Trends Endocrinol. Metab. 20 (4), 163–170. 10.1016/j.tem.2009.01.006 19359194 PMC2732121

[B233] Martin B. Chen H. Daimon C. M. Chadwick W. Siddiqui S. Maudsley S. (2013). Plurigon: three dimensional visualization and classification of high-dimensionality data. Front. Physiol. 4, 190. 10.3389/fphys.2013.00190 23885241 PMC3717481

[B234] Martin B. Chadwick W. Janssens J. Premont R. T. Schmalzigaug R. Becker K. G. (2016). GIT2 Acts as a Systems-Level Coordinator of Neurometabolic Activity and Pathophysiological Aging. Front. Endocrinol. (Lausanne) 6, 191. 10.3389/fendo.2015.00191 26834700 PMC4716144

[B235] Martin B. Wang R. Cong W. N. Daimon C. M. Wu W. W. Ni B. (2017). Altered learning, memory, and social behavior in type 1 taste receptor subunit 3 knock-out mice are associated with neuronal dysfunction. J. Biol. Chem. 292 (27), 11508–11530. 10.1074/jbc.M116.773820 28522608 PMC5500814

[B236] Masaoka A. Gassman N. R. Kedar P. S. Prasad R. Hou E. W. Horton J. K. (2012). HMGN1 protein regulates poly(ADP-ribose) polymerase-1 (PARP-1) self-PARylation in mouse fibroblasts. J. Biol. Chem. 287 (33), 27648–27668. 10.1074/jbc.M112.370759 22736760 PMC3431713

[B237] Matsukawa J. Matsuzawa A. Takeda K. Ichijo H. (2004). The ASK1-MAP kinase cascades in mammalian stress response. J. Biochem. 136 (3), 261–266. 10.1093/jb/mvh134 15598880

[B238] Maudsley S. Davidson L. Pawson A. J. Chan R. López de Maturana R. Millar R. P. (2004). Gonadotropin-releasing hormone (GnRH) antagonists promote proapoptotic signaling in peripheral reproductive tumor cells by activating a Galphai-coupling state of the type I GnRH receptor. Cancer Res. 64 (20), 7533–7544. 10.1158/0008-5472.CAN-04-1360 15492280

[B239] Maudsley S. Martin B. Luttrell L. M. (2005). The origins of diversity and specificity in g protein-coupled receptor signaling. J. Pharmacol. Exp. Ther. 314 (2), 485–494. 10.1124/jpet.105.083121 15805429 PMC2656918

[B240] Maudsley S. Patel S. A. Park S. S. Luttrell L. M. Martin B. (2012). Functional signaling biases in G protein-coupled receptors: Game Theory and receptor dynamics. Mini Rev. Med. Chem. 12 (9), 831–840. 10.2174/138955712800959071 22681251 PMC6013268

[B241] Maudsley S. Siddiqui S. Martin B. (2013). Systems analysis of arrestin pathway functions. Prog. Mol. Biol. Transl. Sci. 118, 431–467. 10.1016/B978-0-12-394440-5.00017-6 23764064

[B242] Maudsley S. Martin B. Gesty-Palmer D. Cheung H. Johnson C. Patel S. (2015). Delineation of a conserved arrestin-biased signaling repertoire *in vivo* . Mol. Pharmacol. 87 (4), 706–717. 10.1124/mol.114.095224 25637603 PMC4366796

[B243] Maudsley S. Martin B. Janssens J. Etienne H. Jushaj A. van Gastel J. (2016). Informatic deconvolution of biased GPCR signaling mechanisms from *in vivo* pharmacological experimentation. Methods 92, 51–63. 10.1016/j.ymeth.2015.05.013 25986936 PMC4646739

[B244] Maudsley S. Devanarayan V. Martin B. Geerts H. Brain Health Modeling Initiative BHMI (2018). Intelligent and effective informatic deconvolution of “Big Data” and its future impact on the quantitative nature of neurodegenerative disease therapy. Alzheimers Dement. 14, 961–975. 10.1016/j.jalz.2018.01.014 29551332

[B245] Maudsley S. Leysen H. van Gastel J. Martin B. (2021). Systems Pharmacology: Enabling Multidimensional Therapeutics. Compr. Pharmacol. Hardcover. 10.1016/b978-0-12-820472-6.00017-7

[B246] Mayer E. A. Naliboff B. D. Chang L. Coutinho S. V. (2001). V. Stress and irritable bowel syndrome. Am. J. Physiol. Gastrointest. Liver Physiol. 280 (4), G519–524. 10.1152/ajpgi.2001.280.4.G519 11254476

[B247] Mazzoccoli G. Laukkanen M. O. Vinciguerra M. Colangelo T. Colantuoni V. (2016). A Timeless Link Between Circadian Patterns and Disease. Trends Mol. Med. 22 (1), 68–81. 10.1016/j.molmed.2015.11.007 26691298

[B248] McClintock M. K. Dale W. Laumann E. O. Waite L. (2016). Empirical redefinition of comprehensive health and well-being in the older adults of the United States. Proc. Natl. Acad. Sci. U. S. A. 113 (22), E3071–3080. 10.1073/pnas.1514968113 27185911 PMC4896706

[B249] McDonald P. H. Chow C. W. Miller W. E. Laporte S. A. Field M. E. Lin F. T. (2000). Beta-arrestin 2: a receptor-regulated MAPK scaffold for the activation of JNK3. Science 290 (5496), 1574–1577. 10.1126/science.290.5496.1574 11090355

[B250] McEwen B. S. (1998). Stress, adaptation, and disease. Allostasis and allostatic load. Ann. N. Y. Acad. Sci. 840, 33–44. 10.1111/j.1749-6632.1998.tb09546.x 9629234

[B251] Mesgarzadeh J. S. Buxbaum J. N. Wiseman R. L. (2022). Stress-responsive regulation of extracellular proteostasis. J. Cell Biol. 221 (4), e202112104. 10.1083/jcb.202112104 35191945 PMC8868021

[B252] Meyer R. C. Giddens M. M. Schaefer S. A. Hall R. A. (2013). GPR37 and GPR37L1 are receptors for the neuroprotective and glioprotective factors prosaptide and prosaposin. Proc. Natl. Acad. Sci. U. S. A. 110 (23), 9529–9534. 10.1073/pnas.1219004110 23690594 PMC3677493

[B253] Michel G. Matthes H. W. Hachet-Haas M. El Baghdadi K. de Mey J. Pepperkok R. (2014). Plasma membrane translocation of REDD1 governed by GPCRs contributes to mTORC1 activation. J. Cell Sci. 127 (Pt 4), 773–787. 10.1242/jcs.136432 24338366

[B254] Michna A. Schötz U. Selmansberger M. Zitzelsberger H. Lauber K. Unger K. (2016). Transcriptomic analyses of the radiation response in head and neck squamous cell carcinoma subclones with different radiation sensitivity: time-course gene expression profiles and gene association networks. Radiat. Oncol. 11, 94. 10.1186/s13014-016-0672-0 27455841 PMC4960706

[B255] Miki T. Matsumoto T. Zhao Z. Lee C. C. (2013). p53 regulates Period2 expression and the circadian clock. Nat. Commun. 4, 2444. 10.1038/ncomms3444 24051492 PMC3798035

[B256] Miller W. E. Lefkowitz R. J. (2001). Expanding roles for beta-arrestins as scaffolds and adapters in GPCR signaling and trafficking. Curr. Opin. Cell Biol. 13 (2), 139–145. 10.1016/s0955-0674(00)00190-3 11248546

[B257] Miller W. E. McDonald P. H. Cai S. F. Field M. E. Davis R. J. Lefkowitz R. J. (2001). Identification of a motif in the carboxyl terminus of beta-arrestin2 responsible for activation of JNK3. J. Biol. Chem. 276 (30), 27770–27777. 10.1074/jbc.M102264200 11356842

[B258] Miller E. Yang J. DeRan M. Wu C. Su A. I. Bonamy G. M. (2012). Identification of serum-derived sphingosine-1-phosphate as a small molecule regulator of YAP. Chem. Biol. 19 (8), 955–962. 10.1016/j.chembiol.2012.07.005 22884261

[B259] Mo J. S. Yu F. X. Gong R. Brown J. H. Guan K. L. (2012). Regulation of the Hippo-YAP pathway by protease-activated receptors (PARs). Genes Dev. 26 (19), 2138–2143. 10.1101/gad.197582.112 22972936 PMC3465735

[B260] Modrich P. (2016). Mechanisms in eukaryotic mismatch repair. J. Biol. Chem. 281, 30305–30309. 10.1074/jbc.R600022200 16905530 PMC2234602

[B261] Moya E. A. Powell F. L. (2018). Serotonin and Adenosine G-protein Coupled Receptor Signaling for Ventilatory Acclimatization to Sustained Hypoxia. Front. Physiol. 9, 860. 10.3389/fphys.2018.00860 30072908 PMC6059110

[B262] Müller R. U. Schermer B. (2020). Hippo signaling-a central player in cystic kidney disease? Pediatr. Nephrol. 35 (7), 1143–1152. 10.1007/s00467-019-04299-3 31297585

[B263] Murthy V. L. Yu B. Wang W. Zhang X. Alkis T. Pico A. R. (2020). Molecular Signature of Multisystem Cardiometabolic Stress and Its Association With Prognosis. JAMA Cardiol. 5 (10), 1144–1153. 10.1001/jamacardio.2020.2686 32717046 PMC7376474

[B264] Musi C. A. Marchini G. Giani A. Tomaselli G. Priori E. C. Colnaghi L. (2022). Colocalization and Interaction Study of Neuronal JNK3, JIP1, and β-Arrestin2 Together with PSD95. Int. J. Mol. Sci. 23 (8), 4113. 10.3390/ijms23084113 35456931 PMC9024448

[B265] Myerson R. (1991). Game Theory: Analysis of Conflict. Cambridge, MA. London England: Harvard University Press.

[B266] Naderi Yeganeh P. Richardson C. Saule E. Loraine A. Taghi Mostafavi M. (2020). Revisiting the use of graph centrality models in biological pathway analysis. BioData Min. 13, 5. 10.1186/s13040-020-00214-x 32549913 PMC7296696

[B267] Nair R. R. Madiwale S. V. Saini D. K. (2018). Clampdown of inflammation in aging and anticancer therapies by limiting upregulation and activation of GPCR. CXCR4. NPJ Aging Mech. Dis. 4, 9. 10.1038/s41514-018-0028-0 30181898 PMC6117261

[B268] Nakamura S. Miwa M. Morita Y. Ohkura S. Yamamura T. Wakabayashi Y. (2021). Neurokinin 3 receptor-selective agonist, senktide, decreases core temperature in Japanese Black cattle. Domest. Anim. Endocrinol. 74, 106522. 10.1016/j.domaniend.2020.106522 32841888

[B269] Nestler E. J. Russo S. J. (2024). Neurobiological basis of stress resilience. Neuron 112 (12), 1911–1929. 10.1016/j.neuron.2024.05.001 38795707 PMC11189737

[B270] Nieto A. Hara M. R. Quereda V. Grant W. Saunders V. Xiao K. (2020). βarrestin-1 regulates DNA repair by acting as an E3-ubiquitin ligase adaptor for 53BP1. Cell Death Differ. 27 (4), 1200–1213. 10.1038/s41418-019-0406-6 31506606 PMC7206116

[B271] Nii T. Prabhu V. V. Ruvolo V. Madhukar N. Zhao R. Mu H. (2019). Imipridone ONC212 activates orphan G protein-coupled receptor GPR132 and integrated stress response in acute myeloid leukemia. Leukemia 33 (12), 2805–2816. 10.1038/s41375-019-0491-z 31127149 PMC6874902

[B272] Nijboer C. H. Kavelaars A. Vroon A. Groenendaal F. van Bel F. Heijnen C. J. (2008). Low endogenous G-protein-coupled receptor kinase 2 sensitizes the immature brain to hypoxia-ischemia-induced gray and white matter damage. J. Neurosci. 28 (13), 3324–3332. 10.1523/JNEUROSCI.4769-07.2008 18367599 PMC6670601

[B273] Novoa I. Zeng H. Harding H. P. Ron D. (2001). Feedback inhibition of the unfolded protein response by GADD34-mediated dephosphorylation of eIF2alpha. J. Cell Biol. 153 (5), 1011–1022. 10.1083/jcb.153.5.1011 11381086 PMC2174339

[B274] Okamoto M. Okamoto M. Yamada K. Yoshimasa Y. Kosaki A. Kono S. (1992). Insulin resistance in Werner's syndrome. Mech. Ageing Dev. 63 (1), 11–25. 10.1016/0047-6374(92)90013-4 1602838

[B275] Olsson M. G. Nilsson E. J. Rutardóttir S. Paczesny J. Pallon J. Akerström B. (2010). Bystander cell death and stress response is inhibited by the radical scavenger α(1)-microglobulin in irradiated cell cultures. Radiat. Res. 174 (5), 590–600. 10.1667/RR2213.1 20954860

[B276] Ondaro J. Hernandez-Eguiazu H. Garciandia-Arcelus M. Loera-Valencia R. Rodriguez-Gómez L. Jiménez-Zúñiga A. (2022). Defects of Nutrient Signaling and Autophagy in Neurodegeneration. Front. Cell Dev. Biol. 10, 836196. 10.3389/fcell.2022.836196 35419363 PMC8996160

[B277] Ortillon J. Le Bail J. C. Villard E. Léger B. Poirier B. Girardot C. (2021). High Glucose Activates YAP Signaling to Promote Vascular Inflammation. Front. Physiol. 12, 665994. 10.3389/fphys.2021.665994 34149446 PMC8213390

[B278] Pakos-Zebrucka K. Koryga I. Mnich K. Ljujic M. Samali A. Gorman A. M. (2016). The integrated stress response. EMBO Rep. 17 (10), 1374–1395. 10.15252/embr.201642195 27629041 PMC5048378

[B279] Pandey S. Kumari P. Baidya M. Kise R. Cao Y. Dwivedi-Agnihotri H. (2021). Intrinsic bias at non-canonical, β-arrestin-coupled seven transmembrane receptors. Mol. Cell 81 (22), 4605–4621. 10.1016/j.molcel.2021.09.007 34582793 PMC7612807

[B280] Papaconstantinou J. (2019). The Role of Signaling Pathways of Inflammation and Oxidative Stress in Development of Senescence and Aging Phenotypes in Cardiovascular Disease. Cells 8 (11), 1383. 10.3390/cells8111383 31689891 PMC6912541

[B281] Pauwels P. J. Tardif S. Wurch T. Colpaert F. C. (2000). Facilitation of constitutive alpha(2A)-adrenoceptor activity by both single amino acid mutation (Thr(373)Lys) and g(alphao) protein coexpression: evidence for inverse agonism. J. Pharmacol. Exp. Ther. 292 (2), 654–663. 10.1016/S0022-3565(24)35336-4 10640303

[B282] Pierce K. L. Maudsley S. Daaka Y. Luttrell L. M. Lefkowitz R. J. (2000). Role of endocytosis in the activation of the extracellular signal-regulated kinase cascade by sequestering and nonsequestering G protein-coupled receptors. Proc. Natl. Acad. Sci. U. S. A. 97 (4), 1489–1494. 10.1073/pnas.97.4.1489 10677489 PMC26461

[B283] Prabhu V. V. Morrow S. Rahman Kawakibi A. Zhou L. Ralff M. Ray J. (2020). ONC201 and imipridones: Anti-cancer compounds with clinical efficacy. Neoplasia 22 (12), 725–744. 10.1016/j.neo.2020.09.005 33142238 PMC7588802

[B284] Premont R. T. Claing A. Vitale N. Freeman J. L. Pitcher J. A. Patton W. A. (1998). beta2-Adrenergic receptor regulation by GIT1, a G protein-coupled receptor kinase-associated ADP ribosylation factor GTPase-activating protein. Proc. Natl. Acad. Sci. U. S. A. 95 (24), 14082–14087. 10.1073/pnas.95.24.14082 9826657 PMC24330

[B285] Premont R. T. Claing A. Vitale N. Perry S. J. Lefkowitz R. J. (2000). The GIT family of ADP-ribosylation factor GTPase-activating proteins. Functional diversity of GIT2 through alternative splicing. J. Biol. Chem. 275 (29), 22373–22380. 10.1074/jbc.275.29.22373 10896954

[B286] Pydi S. P. Barella L. F. Zhu L. Meister J. Rossi M. Wess J. (2022). β-Arrestins as Important Regulators of Glucose and Energy Homeostasis. Annu. Rev. Physiol. 84, 17–40. 10.1146/annurev-physiol-060721-092948 34705480

[B287] Rada C. C. Mejia-Pena H. Grimsey N. J. Canto Cordova I. Olson J. Wozniak J. M. (2021). Heat shock protein 27 activity is linked to endothelial barrier recovery after proinflammatory GPCR-induced disruption. Sci. Signal 14 (698), eabc1044. 10.1126/scisignal.abc1044 34516752 PMC8538426

[B288] Randazzo P. A. Terui T. Sturch S. Kahn R. A. (1994). The amino terminus of ADP-ribosylation factor (ARF) 1 is essential for interaction with Gs and ARF GTPase-activating protein. J. Biol. Chem. 269 (47), 29490–29498. 10.1016/S0021-9258(18)43906-3 7961931

[B289] Recchia A. G. De Francesco E. M. Vivacqua A. Sisci D. Panno M. L. Andò S. (2011). The G protein-coupled receptor 30 is up-regulated by hypoxia-inducible factor-1alpha (HIF-1alpha) in breast cancer cells and cardiomyocytes. J. Biol. Chem 286 (12), 10773–10782. 10.1074/jbc.M110.172247 21266576 PMC3060528

[B290] Reichert S. Stier A. (2017). Does oxidative stress shorten telomeres *in vivo*? A review. Biol. Lett. 13 (12), 20170463. 10.1098/rsbl.2017.0463 29212750 PMC5746531

[B291] Ricoult S. J. Manning B. D. (2013). The multifaceted role of mTORC1 in the control of lipid metabolism. EMBO Rep. 14 (3), 242–251. 10.1038/embor.2013.5 23399656 PMC3589096

[B292] Rodriguez-Rocha H. Garcia-Garcia A. Panayiotidis M. I. Franco R. (2011). DNA damage and autophagy. Mutat. Res. 711 (1-2), 158–166. 10.1016/j.mrfmmm.2011.03.007 21419786 PMC3105359

[B293] Rojanathammanee L. Harmon E. B. Grisanti L. A. Govitrapong P. Ebadi M. Grove B. D. (2009). The 27-kDa heat shock protein confers cytoprotective effects through a beta 2-adrenergic receptor agonist-initiated complex with beta-arrestin. Mol. Pharmacol. 75 (4), 855–865. 10.1124/mol.108.053397 19176359 PMC2684928

[B294] Rosanò L. Cianfrocca R. Tocci P. Spinella F. Di Castro V. Spadaro F. (2013). β-arrestin-1 is a nuclear transcriptional regulator of endothelin-1-induced β-catenin signaling. Oncogene 32 (42), 5066–5077. 10.1038/onc.2012.527 23208497

[B295] Ross E. M. Wilkie T. M. (2000). GTPase-activating proteins for heterotrimeric G proteins: regulators of G protein signaling (RGS) and RGS-like proteins. Annu. Rev. Biochem. 69, 795–827. 10.1146/annurev.biochem.69.1.795 10966476

[B296] Sagan S. Chassaing G. Pradier L. Lavielle S. (1996). Tachykinin peptides affect differently the second messenger pathways after binding to CHO-expressed human NK-1 receptors. J. Pharmacol. Exp. Ther. 276 (3), 1039–1048. 10.1016/S0022-3565(25)12378-1 8786533

[B297] Sancar A. Lindsey-Boltz L. A. Unsal-Kaçmaz K. Linn S. (2004). Molecular mechanisms of mammalian DNA repair and the DNA damage checkpoints. Annu. Rev. Biochem. 73, 39–85. 10.1146/annurev.biochem.73.011303.073723 15189136

[B298] Sankar P. L. Parker L. S. (2017). The Precision Medicine Initiative's All of Us Research Program: an agenda for research on its ethical, legal, and social issues. Genet. Med. 19 (7), 743–750. 10.1038/gim.2016.183 27929525

[B299] Santoro M. G. (2000). Heat shock factors and the control of the stress response. Biochem. Pharmacol. 59 (1), 55–63. 10.1016/s0006-2952(99)00299-3 10605935

[B300] Santos-Otte P. Leysen H. van Gastel J. Hendrickx J. O. Martin B. Maudsley S. (2019). G Protein-Coupled Receptor Systems and Their Role in Cellular Senescence. Comput. Struct. Biotechnol. J. 17, 1265–1277. 10.1016/j.csbj.2019.08.005 31921393 PMC6944711

[B301] Saponaro C. Sergio S. Coluccia A. De Luca M. La Regina G. Mologni L. (2018). β-catenin knockdown promotes NHERF1-mediated survival of colorectal cancer cells: implications for a double-targeted therapy. Oncogene 37 (24), 3301–3316. 10.1038/s41388-018-0170-y 29551770 PMC6002344

[B302] Schattauer S. S. Bedini A. Summers F. Reilly-Treat A. Andrews M. M. Land B. B. (2019). Reactive oxygen species (ROS) generation is stimulated by κ opioid receptor activation through phosphorylated c-Jun N-terminal kinase and inhibited by p38 mitogen-activated protein kinase (MAPK) activation. J. Biol. Chem. 294 (45), 16884–16896. 10.1074/jbc.RA119.009592 31575661 PMC6851317

[B303] Schmalzigaug R. Rodriguiz R. M. Phillips L. E. Davidson C. E. Wetsel W. C. Premont R. T. (2009). Anxiety-like behaviors in mice lacking GIT2. Neurosci. Lett. 451 (2), 156–161. 10.1016/j.neulet.2008.12.034 19114090 PMC2648396

[B304] Schuermann D. Mevissen M. (2021). Manmade Electromagnetic Fields and Oxidative Stress-Biological Effects and Consequences for Health. Int. J. Mol. Sci. 22 (7), 3772. 10.3390/ijms22073772 33917298 PMC8038719

[B305] Sedivy R. (1999). Chaodynamic loss of complexity and self-similarity in cancer. Med. Hypotheses 52 (4), 271–274. 10.1054/mehy.1997.0653 10465661

[B306] Senft D. Ronai Z. A. (2015). UPR, autophagy, and mitochondria crosstalk underlies the ER stress response. Trends Biochem. Sci. 40 (3), 141–148. 10.1016/j.tibs.2015.01.002 25656104 PMC4340752

[B307] Seo S. B. Lee J. J. Yun H. H. Im C. N. Kim Y. S. Ko J. H. (2018a). 14-3-3β Depletion Drives a Senescence Program in Glioblastoma Cells Through the ERK/SKP2/p27 Pathway. Mol. Neurobiol. 55 (2), 1259–1270. 10.1007/s12035-017-0407-8 28116547

[B308] Seo S. K. Kim N. Lee J. H. Kim S. M. Lee S. Y. Bae J. W. (2018b). β-arrestin2 Affects Cardiac Progenitor Cell Survival through Cell Mobility and Tube Formation in Severe Hypoxia. Korean Circ. J. 48 (4), 296–309. 10.4070/kcj.2017.0119 29625512 PMC5889979

[B309] Sharma V. K. Yang X. Kim S. K. Mafi A. Saiz-Sanchez D. Villanueva-Anguita P. (2021). Novel interaction between neurotrophic factor-α1/carboxypeptidase E and serotonin receptor, 5-HTR1E, protects human neurons against oxidative/neuroexcitotoxic stress via β-arrestin/ERK signaling. Cell Mol. Life Sci. 79 (1), 24. 10.1007/s00018-021-04021-3 34966948 PMC8732845

[B310] Shen Z. Nickoloff J. A. (2007). “DNA Repair, Genetic Instability, and Cancer,” in World Scientific. River Edge, NJ, USA, 119–156.

[B311] Shirazi-Beechey S. P. Daly K. Al-Rammahi M. Moran A. W. Bravo D. (2014). Role of nutrient-sensing taste 1 receptor (T1R) family members in gastrointestinal chemosensing. Br. J. Nutr. 111 (Suppl. 1), S8–15. 10.1017/S0007114513002286 24382171

[B312] Shrivastav M. De Haro L. P. Nickoloff J. A. (2008). Regulation of DNA double-strand break repair pathway choice. Cell Res. 18 (1), 134–147. 10.1038/cr.2007.111 18157161

[B313] Siddiqui S. Lustig A. Carter A. Sankar M. Daimon C. M. Premont R. T. (2017). Genomic deletion of GIT2 induces a premature age-related thymic dysfunction and systemic immune system disruption. Aging (Albany NY) 9 (3), 706–740. 10.18632/aging.101185 28260693 PMC5391227

[B314] Sikora E. Bielak-Zmijewska A. Mosieniak G. (2014). Cellular senescence in ageing, age-related disease and longevity. Curr. Vasc. Pharmacol. 12 (5), 698–706. 10.2174/1570161111666131219094045 24350932

[B315] Silva L. E. Souza R. C. Kitano E. S. Monteiro L. F. Iwai L. K. Forti F. L. (2019). Proteomic and Interactome Approaches Reveal PAK4, PHB-2, and 14-3-3η as Targets of Overactivated Cdc42 in Cellular Responses to Genomic Instability. J. Proteome Res. 18 (10), 3597–3614. 10.1021/acs.jproteome.9b00260 31478661

[B316] Sistonen L. Sarge K. D. Morimoto R. I. (1994). Human heat shock factors 1 and 2 are differentially activated and can synergistically induce hsp70 gene transcription. Mol. Cell Biol. 14 (3), 2087–2099. 10.1128/mcb.14.3.2087 8114740 PMC358569

[B317] Sleimen-Malkoun R. Temprado J. J. Hong S. L. (2014). Aging induced loss of complexity and dedifferentiation: consequences for coordination dynamics within and between brain, muscular and behavioral levels. Front. Aging Neurosci. 6, 140. 10.3389/fnagi.2014.00140 25018731 PMC4073624

[B318] Smith J. M. (1981). Evolution and the Theory of Games. Cambridge, United Kingdom: Cambridge University press.

[B319] Smith J. Price G. (1973). The Logic of Animal Conflict. Nature 246, 15–18. 10.1038/246015a0

[B320] Smith J. S. Lefkowitz R. J. Rajagopal S. (2018). Biased signalling: from simple switches to allosteric microprocessors. Nat. Rev. Drug Discov. 17 (4), 243–260. 10.1038/nrd.2017.229 29302067 PMC5936084

[B321] Smith A. M. Walsh J. R. Long J. Davis C. B. Henstock P. Hodge M. R. (2020). Standard machine learning approaches outperform deep representation learning on phenotype prediction from transcriptomics data. BMC Bioinforma. 21 (1), 119. 10.1186/s12859-020-3427-8 32197580 PMC7085143

[B322] Stelzner T. J. O'Brien R. F. Sato K. Weil J. V. (1988). Hypoxia-induced increases in pulmonary transvascular protein escape in rats. Modulation by glucocorticoids. J. Clin. Invest 82 (6), 1840–1847. 10.1172/JCI113800 3198758 PMC442762

[B323] Sterling P. Eyer J. (1988). “Handbook of Life Stress,” in Cognition and Health. Editors Fisher S. Copyright J. R. (John Wiley), 8.

[B324] Stoeber M. Jullié D. Lobingier B. T. Laeremans T. Steyaert J. Schiller P. W. (2018). A Genetically Encoded Biosensor Reveals Location Bias of Opioid Drug Action. Neuron 98 (5), 963–976. 10.1016/j.neuron.2018.04.021 29754753 PMC6481295

[B325] Štorcelová M. Vicián M. Reis R. Zeman M. Herichová I. (2013). Expression of cell cycle regulatory factors hus1, gadd45a, rb1, cdkn2a and mre11a correlates with expression of clock gene per2 in human colorectal carcinoma tissue. Mol. Biol. Rep. 40 (11), 6351–6361. 10.1007/s11033-013-2749-2 24062075

[B326] Strepkos D. Markouli M. Papavassiliou K. A. Papavassiliou A. G. Piperi C. (2022). Emerging roles for the YAP/TAZ transcriptional regulators in brain tumour pathology and targeting options. Neuropathol. Appl. Neurobiol. 48 (2), e12762. 10.1111/nan.12762 34409639

[B327] Sturchler E. Feurstein D. McDonald P. Duckett D. (2010). Mechanism of oxidative stress-induced ASK1-catalyzed MKK6 phosphorylation. Biochemistry 49 (19), 4094–4102. 10.1021/bi100010j 20364819

[B328] Sturchler E. Chen W. Spicer T. Hodder P. McDonald P. Duckett D. (2014). Development of an HTS-compatible assay for the discovery of ASK1 signalosome inhibitors using alphascreen technology. Assay. Drug Dev. Technol. 12 (4), 229–237. 10.1089/adt.2013.558 24831789 PMC4025566

[B329] Syed N. Chavan S. Sahasrabuddhe N. A. Renuse S. Sathe G. Nanjappa V. (2015). Silencing of high-mobility group box 2 (HMGB2) modulates cisplatin and 5-fluorouracil sensitivity in head and neck squamous cell carcinoma. Proteomics 15 (2-3), 383–393. 10.1002/pmic.201400338 25327479 PMC4528963

[B330] Takahashi J. S. Hong H. K. Ko C. H. McDearmon E. L. (2008). The genetics of mammalian circadian order and disorder: implications for physiology and disease. Nat. Rev. Genet. 9 (10), 764–775. 10.1038/nrg2430 18802415 PMC3758473

[B331] Tan S. Li L. Chen T. Chen X. Tao L. Lin X. (2015). β-Arrestin-1 protects against endoplasmic reticulum stress/p53-upregulated modulator of apoptosis-mediated apoptosis via repressing p-p65/inducible nitric oxide synthase in portal hypertensive gastropathy. Free Radic. Biol. Med. 87, 69–83. 10.1016/j.freeradbiomed.2015.06.004 26119788

[B332] Tao C. Huang S. Wang Y. Wei G. Zhang Y. Qi D. (2015). Changes in white and brown adipose tissue microRNA expression in cold-induced mice. Biochem. Biophys. Res. Commun. 463 (3), 193–199. 10.1016/j.bbrc.2015.05.014 25983326

[B333] Thapa D. Stoner M. W. Zhang M. Xie B. Manning J. R. Guimaraes D. (2018). Adropin regulates pyruvate dehydrogenase in cardiac cells via a novel GPCR-MAPK-PDK4 signaling pathway. Redox Biol. 18, 25–32. 10.1016/j.redox.2018.06.003 29909017 PMC6008287

[B334] Theccanat T. Philip J. L. Razzaque A. M. Ludmer N. Li J. Xu X. (2016). Regulation of cellular oxidative stress and apoptosis by G protein-coupled receptor kinase-2; The role of NADPH oxidase 4. Cell Signal 28 (3), 190–203. 10.1016/j.cellsig.2015.11.013 26631573 PMC4837949

[B335] Tiwari P. Fanibunda S. E. Kapri D. Vasaya S. Pati S. Vaidya V. A. (2021). GPCR signaling: role in mediating the effects of early adversity in psychiatric disorders. FEBS J. 288 (8), 2602–2621. 10.1111/febs.15738 33523596

[B336] Tocci P. Blandino G. Bagnato A. (2021). YAP and endothelin-1 signaling: an emerging alliance in cancer. J. Exp. Clin. Cancer Res. 40 (1), 27. 10.1186/s13046-021-01827-8 33422090 PMC7797087

[B337] Torrence M. E. MacArthur M. R. Hosios A. M. Valvezan A. J. Asara J. M. Mitchell J. R. (2021). The mTORC1-mediated activation of ATF4 promotes protein and glutathione synthesis downstream of growth signals. Elife 10, e63326. 10.7554/eLife.63326 33646118 PMC7997658

[B338] Totzeck M. Hendgen-Cotta U. B. Kelm M. Rassaf T. (2014). Crosstalk between nitrite, myoglobin and reactive oxygen species to regulate vasodilation under hypoxia. PLoS One 9 (8), e105951. 10.1371/journal.pone.0105951 25148388 PMC4141839

[B339] Tripathi A. Kumar M. Kaur P. Kumar B. Sagi S. S. K. (2020). Efficacy of Quercetin as a potent sensitizer of β2-AR in combating the impairment of fluid clearance in lungs of rats under hypoxia. Respir. Physiol. Neurobiol. 273, 103334. 10.1016/j.resp.2019.103334 31689533

[B340] Trivedi R. Jurivich D. A. (2020). A molecular perspective on age-dependent changes to the heat shock axis. Exp. Gerontol. 137, 110969. 10.1016/j.exger.2020.110969 32407864

[B341] Urano F. Wang X. Bertolotti A. Zhang Y. Chung P. Harding H. P. (2000). Coupling of stress in the ER to activation of JNK protein kinases by transmembrane protein kinase IRE1. Science 287 (5453), 664–666. 10.1126/science.287.5453.664 10650002

[B342] van Gastel J. Hendrickx J. O. Leysen H. Santos-Otte P. Luttrell L. M. Martin B. (2018a). β-Arrestin Based Receptor Signaling Paradigms: Potential Therapeutic Targets for Complex Age-Related Disorders. Front. Pharmacol. 9, 1369. 10.3389/fphar.2018.01369 30546309 PMC6280185

[B343] van Gastel J. Boddaert J. Jushaj A. Premont R. T. Luttrell L. M. Janssens J. (2018b). GIT2-A keystone in ageing and age-related disease. Ageing Res. Rev. 43, 46–63. 10.1016/j.arr.2018.02.002 29452267

[B344] van Gastel J. Leysen H. Santos-Otte P. Hendrickx J. O. Azmi A. Martin B. (2019a). The RXFP3 receptor is functionally associated with cellular responses to oxidative stress and DNA damage. Aging (Albany NY) 11 (23), 11268–11313. 10.18632/aging.102528 31794429 PMC6932917

[B345] van Gastel J. Cai H. Cong W. N. Chadwick W. Daimon C. Leysen H. (2019b). Multidimensional informatic deconvolution defines gender-specific roles of hypothalamic GIT2 in aging trajectories. Mech. Ageing Dev. 184, 111150. 10.1016/j.mad.2019.111150 31574270

[B346] van Gastel J. Leysen H. Boddaert J. Vangenechten L. Luttrell L. M. Martin B. (2021). Aging-related modifications to G protein-coupled receptor signaling diversity. Pharmacol. Ther. 223, 107793. 10.1016/j.pharmthera.2020.107793 33316288

[B347] Verbeke P. Fonager J. Clark B. F. Rattan S. I. (2001). Heat shock response and ageing: mechanisms and applications. Cell Biol. Int. 25 (9), 845–857. 10.1006/cbir.2001.0789 11518492

[B348] Vitale N. Patton W. A. Moss J. Vaughan M. Lefkowitz R. J. Premont R. T. (2000). GIT proteins, A novel family of phosphatidylinositol 3,4, 5-trisphosphate-stimulated GTPase-activating proteins for ARF6. J. Biol. Chem. 275 (18), 13901–13906. 10.1074/jbc.275.18.13901 10788515

[B349] Wang C. J. Chidiac P. (2019). RGS2 promotes the translation of stress-associated proteins ATF4 and CHOP via its eIF2B-inhibitory domain. Cell Signal 59, 163–170. 10.1016/j.cellsig.2019.02.007 30826455

[B350] Wang H. M. Dong J. H. Li Q. Hu Q. Ning S. L. Zheng W. (2014a). A stress response pathway in mice upregulates somatostatin level and transcription in pancreatic delta cells through Gs and β-arrestin 1. Diabetologia 57 (9), 1899–1910. 10.1007/s00125-014-3290-0 24947582

[B351] Wang P. Xu T. Y. Wei K. Guan Y. F. Wang X. Xu H. (2014b). ARRB1/β-arrestin-1 mediates neuroprotection through coordination of BECN1-dependent autophagy in cerebral ischemia. Autophagy 10 (9), 1535–1548. 10.4161/auto.29203 24988431 PMC4206533

[B352] Wang J. Song Y. Li H. Shen Q. Shen J. An X. (2016). Exacerbated cardiac fibrosis induced by β-adrenergic activation in old mice due to decreased AMPK activity. Clin. Exp. Pharmacol. Physiol. 43 (11), 1029–1037. 10.1111/1440-1681.12622 27389807

[B353] Wang Y. Su G. F. Huang Z. X. Wang Z. G. Zhou P. J. Fan J. L. (2020a). Cepharanthine hydrochloride induces mitophagy targeting GPR30 in hepatocellular carcinoma (HCC). Expert Opin. Ther. Targets 24 (4), 389–402. 10.1080/14728222.2020.1737013 32106726

[B354] Wang J. X. Sullivan D. K. Wells A. C. Chen J. H. (2020b). ClinicNet: machine learning for personalized clinical order set recommendations. JAMIA Open 3 (2), 216–224. 10.1093/jamiaopen/ooaa021 32734162 PMC7382624

[B355] Wang Y. Feng Y. Wang T. Ma Y. Gao P. Chen J. (2021). Drug-coated balloon for vertebral artery origin stenosis: a pilot study. J. Neurointerv Surg. 13 (9), 827–830. 10.1136/neurintsurg-2020-016723 33067258

[B356] Wedegaertner H. Pan W. A. Gonzalez C. C. Gonzalez D. J. Trejo J. (2022). The α-Arrestin ARRDC3 Is an Emerging Multifunctional Adaptor Protein in Cancer. Antioxid. Redox Signal 36 (13-15), 1066–1079. 10.1089/ars.2021.0193 34465145 PMC9127825

[B357] Whitwell H. J. Bacalini M. G. Blyuss O. Chen S. Garagnani P. Gordleeva S. Y. (2020). The Human Body as a Super Network: Digital Methods to Analyze the Propagation of Aging. Front. Aging Neurosci. 12, 136. 10.3389/fnagi.2020.00136 32523526 PMC7261843

[B358] Willoughby E. A. Collins M. K. (2005). Dynamic interaction between the dual specificity phosphatase MKP7 and the JNK3 scaffold protein beta-arrestin 2. J. Biol. Chem. 280 (27), 25651–25658. 10.1074/jbc.M501926200 15888437

[B359] Wodrich A. P. K. Scott A. W. Shukla A. K. Harris B. T. Giniger E. (2022). The Unfolded Protein Responses in Health, Aging, and Neurodegeneration: Recent Advances and Future Considerations. Front. Mol. Neurosci. 15, 831116. 10.3389/fnmol.2022.831116 35283733 PMC8914544

[B360] Won H. Mah W. Kim E. Kim J. W. Hahm E. K. Kim M. H. (2011). GIT1 is associated with ADHD in humans and ADHD-like behaviors in mice. Nat. Med. 17 (5), 566–572. 10.1038/nm.2330 21499268

[B361] Xu D. L. Hu X. K. Tian Y. F. (2017). Effect of temperature and food restriction on immune function in striped hamsters (Cricetulus barabensis). J. Exp. Biol. 220 (2), 2187–2195. 10.1242/jeb.153601 28381582

[B362] Xu B. Lian S. Guo J. R. Wang J. F. Zhang L. P. Li S. Z. (2019). Activation of the MAPK signaling pathway induces upregulation of pro-apoptotic proteins in the hippocampi of cold stressed adolescent mice. Neurosci. Lett. 699, 97–102. 10.1016/j.neulet.2018.12.028 30711527

[B363] Xu Y. Zhi F. Mao J. Peng Y. Shao N. Balboni G. (2020). δ-opioid receptor activation protects against Parkinson's disease-related mitochondrial dysfunction by enhancing PINK1/Parkin-dependent mitophagy. Aging (Albany NY) 12 (24), 25035–25059. 10.18632/aging.103970 33197884 PMC7803568

[B364] Yegorov Y. E. Poznyak A. V. Nikiforov N. G. Sobenin I. A. Orekhov A. N. (2020). The Link between Chronic Stress and Accelerated Aging. Biomedicines 8 (7), 198. 10.3390/biomedicines8070198 32645916 PMC7400286

[B365] Yeung Y. T. Guerrero-Castilla A. Cano M. Muñoz M. F. Ayala A. Argüelles S. (2019). Dysregulation of the Hippo pathway signaling in aging and cancer. Pharmacol. Res. 143, 151–165. 10.1016/j.phrs.2019.03.018 30910741

[B366] Yoris A. Legaz A. Abrevaya S. Alarco S. López Peláez J. Sánchez R. (2020). Multicentric evidence of emotional impairments in hypertensive heart disease. Sci. Rep. 10 (1), 14131. 10.1038/s41598-020-70451-x 32839479 PMC7445248

[B367] Zhang Z. Hao J. Zhao Z. Ben P. Fang F. Shi L. (2009). beta-Arrestins facilitate ubiquitin-dependent degradation of apoptosis signal-regulating kinase 1 (ASK1) and attenuate H2O2-induced apoptosis. Cell Signal 21 (7), 1195–1206. 10.1016/j.cellsig.2009.03.010 19306926

[B368] Zhang Q. S. Eaton G. J. Diallo C. Freeman T. A. (2016). Stress-Induced Activation of Apoptosis Signal-Regulating Kinase 1 Promotes Osteoarthritis. J. Cell Physiol. 231 (4), 944–953. 10.1002/jcp.25186 26405834 PMC5952048

[B369] Zhang W. Sakoda H. Nakazato Y. Islam M. N. Pattou F. Kerr-Conte J. (2021a). Neuromedin U uses Gαi2 and Gαo to suppress glucose-stimulated Ca2+ signaling and insulin secretion in pancreatic β cells. PLoS One 16 (4), e0250232. 10.1371/journal.pone.0250232 33857254 PMC8049253

[B370] Zhang Q. Wang Z. Zhang W. Wen Q. Li X. Zhou J. (2021b). The memory of neuronal mitochondrial stress is inherited transgenerationally via elevated mitochondrial DNA levels. Nat. Cell Biol. 23 (8), 870–880. 10.1038/s41556-021-00724-8 34341532

[B371] Zhang B. Pan C. Feng C. Yan C. Yu Y. Chen Z. (2022a). Role of mitochondrial reactive oxygen species in homeostasis regulation. Redox Rep. 27 (1), 45–52. 10.1080/13510002.2022.2046423 35213291 PMC8890532

[B372] Zhang H. Gong W. Wu S. Perrett S. (2022b). Hsp70 in Redox Homeostasis. Cells 11 (5), 829. 10.3390/cells11050829 35269451 PMC8909019

[B373] Zhao Y. Wu J. Jia H. Wang X. Zheng R. Jiang F. (2019). PROKR2 mutations in idiopathic hypogonadotropic hypogonadism: selective disruption of the binding to a Gα-protein leads to biased signaling. FASEB J. 33 (3), 4538–4546. 10.1096/fj.201801575R 30576231

[B374] Zhao M. Cao N. Gu H. Xu J. Xu W. Zhang D. (2024). AMPK Attenuation of β-Adrenergic Receptor-Induced Cardiac Injury via Phosphorylation of β-Arrestin-1-ser330. Circ. Res. 135 (6), 651–667. 10.1161/CIRCRESAHA.124.324762 39082138

[B375] Zheng Y. Xu L. (2025). Bidirectional crosstalk between microglia and serotonin signaling in neuroinflammation and CNS disorders. Front. Immunol. 16, 1646740. 10.3389/fimmu.2025.1646740 40934003 PMC12417188

[B376] Zheng X. Jia Y. Qiu L. Zeng X. Xu L. Wei M. (2020). A potential target for liver cancer management, lysophosphatidic acid receptor 6 (LPAR6), is transcriptionally up-regulated by the NCOA3 coactivator. J. Biol. Chem. 295 (6), 1474–1488. 10.1074/jbc.RA119.009899 31914406 PMC7008366

[B377] Zhou X. Wang Z. Huang W. Lei Q. Y. (2015). G protein-coupled receptors: bridging the gap from the extracellular signals to the Hippo pathway. Acta Biochim. Biophys. Sin. (Shanghai) 47 (1), 10–15. 10.1093/abbs/gmu108 25491506

[B378] Zhu Z. Hu J. Chen Z. Feng J. Yang X. Liang W. (2022). Transition of acute kidney injury to chronic kidney disease: role of metabolic reprogramming. Metabolism 131, 155194. 10.1016/j.metabol.2022.155194 35346693

[B379] Zhuang L. Ding W. Zhang Q. Ding W. Xu X. Yu X. (2021). TGR5 Attenuated Liver Ischemia-Reperfusion Injury by Activating the Keap1-Nrf2 Signaling Pathway in Mice. Inflammation 44 (3), 859–872. 10.1007/s10753-020-01382-y 33169298

